# The Cell Wall Proteome of *Marchantia polymorpha* Reveals Specificities Compared to Those of Flowering Plants

**DOI:** 10.3389/fpls.2021.765846

**Published:** 2022-01-13

**Authors:** Hasan Kolkas, Thierry Balliau, Josiane Chourré, Michel Zivy, Hervé Canut, Elisabeth Jamet

**Affiliations:** ^1^Laboratoire de Recherche en Sciences Végétales, Université de Toulouse, CNRS, UPS, Auzeville-Tolosane, France; ^2^Université Paris-Saclay, INRAE, CNRS, AgroParisTech, GQE-Le Moulon, PAPPSO, Gif-sur-Yvette, France

**Keywords:** cell wall, *Marchantia polymorpha*, *N*-glycoproteome, phenolics, proteome

## Abstract

Primary plant cell walls are composite extracellular structures composed of three major classes of polysaccharides (pectins, hemicelluloses, and cellulose) and of proteins. The cell wall proteins (CWPs) play multiple roles during plant development and in response to environmental stresses by remodeling the polysaccharide and protein networks and acting in signaling processes. To date, the cell wall proteome has been mostly described in flowering plants and has revealed the diversity of the CWP families. In this article, we describe the cell wall proteome of an early divergent plant, *Marchantia polymorpha*, a Bryophyte which belong to one of the first plant species colonizing lands. It has been possible to identify 410 different CWPs from three development stages of the haploid gametophyte and they could be classified in the same functional classes as the CWPs of flowering plants. This result underlied the ability of *M. polymorpha* to sustain cell wall dynamics. However, some specificities of the *M. polymorpha* cell wall proteome could be highlighted, in particular the importance of oxido-reductases such as class III peroxidases and polyphenol oxidases, D-mannose binding lectins, and dirigent-like proteins. These proteins families could be related to the presence of specific compounds in the *M. polymorpha* cell walls, like mannans or phenolics. This work paves the way for functional studies to unravel the role of CWPs during *M. polymorpha* development and in response to environmental cues.

## Introduction

Plant cell walls are composite structures made of polysaccharidic polymers like pectins, hemicelluloses and cellulose as well as of lignins in lignified secondary walls (Carpita and Gibeaut, 1993; Vanholme et al., 2019). The major pectic components are homogalacturonans, rhamnogalacturonans I and II (Caffall and Mohnen, 2009). Xyloglucans, mannans, and xylans are the hemicelluloses present in the cell walls of all land plants (Scheller and Ulvskov, 2010). Although present in lower amount, cell wall proteins (CWPs) play critical roles in polymer remodeling and in signaling during plant development and in response to environmental clues (Franková and Fry, 2013; Le Gall et al., 2015; Cosgrove, 2016; Novaković et al., 2018). In the recent years, dedicated proteomics approaches have greatly contributed to a better knowledge of the CWPs content of plant cell walls (Albenne et al., 2013). Different flowering plant species have been studied among which *Arabidopsis thaliana* and *Brachypodium distachyon*, which are model plants for dicot and monocot species, respectively (Albenne et al., 2013; Calderan-Rodrigues et al., 2019). The same CWP families could be identified in all the cell wall proteomes described so far (see *WallProtDB*)^1^ and could be grouped in functional classes according to the presence of predicted functional domains and experimental work (Jamet et al., 2008): proteins acting on carbohydrates (PAC), oxido-reductases (OR), proteases (P), proteins possibly related to lipid metabolism (LM), proteins with interaction domains with polysaccharides or proteins (ID), proteins possibly involved in signaling (S), structural proteins (SP), miscellaneous proteins (M), and proteins of yet unknown function (UF).

Thanks to their polymer diversity and to the large battery of CWPs, the plant cell walls have played critical roles in the adaptation of plants to terrestrial habitats (Sørensen et al., 2010). Terrestrialization was enabled by the capacity to synthesize cell walls able to regulate water exchanges, to provide protection against UV radiation and mechanical support. The appearance of a cuticle barrier made of lipid compounds at the epidermal surface has allowed limiting water evaporation and controlling water absorption (Philippe et al., 2020). The terrestrial plants were also exposed to increased levels of UV radiation. The presence of phenolic compounds in cell walls could have provided efficient protection for sessile organisms (Popper et al., 2011; Soriano et al., 2019). Such compounds have also provided (i) mechanical strength to cell walls thus allowing vertical growth and (ii) waterproofing for vessels permitting vertical and long-distance transport of sieve (Popper et al., 2011). The polysaccharide composition of several early divergent plant cell walls as well as their content in aromatic compounds have been analyzed (Peña et al., 2008; Popper, 2008; Soriano et al., 2019). The presence of arabinogalactan proteins (AGPs) in the cell walls of *Marchantia polymorpha* has been recently described (Happ and Classen, 2019). They have been shown to exhibit specific types of *O*-glycosylation, but no precise identification of the protein cores has been reported. A secretome of 45 proteins of *Physcomitrella patens* rhizoids has revealed the presence of 18 proteins predicted to be secreted including three germins, one dirigent protein and one laccase (Martínez-Cortésa et al., 2014). Altogether, to date, the information regarding the cell wall proteomes of early divergent plants like Bryophytes is very limited.

*Marchantia polymorpha* has recently become an attractive model plant for several reasons. It is an early divergent plant and its study should contribute to a better understanding of the evolution of the green lineage (Shimamura, 2016; Delwiche et al., 2017). Its genome has been recently sequenced and is moderately small (∼226 Mb, around 20,000 annotated genes) (Bowman et al., 2017). *M. polymorpha* is easy to grow in laboratory conditions, has a short life cycle, and the haploid gametophyte is the dominant phase (Shimamura, 2016). It can also be easily transformed and genetically modified by gene editing (Delwiche et al., 2017).

The aim of this work was to identify CWPs of *M. polymorpha* at three different stages of its vegetative development. The proteins have been extracted by two complementary methods: (i) extraction of proteins with salt solutions from purified cell walls; (ii) affinity chromatography on Concanavalin A (ConA) to purify *N*-glycoproteins, since secreted proteins go through the secretion pathways where most of them are *N*-glycosylated. The resulting proteomes were combined and compared together and to the proteomes of flowering plants already described. Major protein families could be identified which characterize the *M. polymorpha* cell wall proteome as a particular one. Its specificities are discussed with regard to the cell wall composition.

## Materials and Methods

### Plant Material

*M. polymorpha* of male accession Takaragaike-1 (Tak-1*)* was cultivated through thallus fragmentation on Jiffy pellets (Jiffy Products International AS, Stange, Norway) or maintained asexually through gemma growth on half-strength Gamborg’s B-5 basal medium (GB_1/2_) with minimal organics medium (Sigma-Aldrich, Merck, Darmstadt, Germany) supplemented with 1% sucrose and 1.4% agar with a pH of 5.5. The plants were placed in growth chamber at 22°C under a 8 h dark/16 h light photoperiod and at 100 μE m^–2^ s^–1^ light intensity. The 2 week-old thalli were only grown *in vitro*. The 3 week-old thalli were grown for 2 weeks *in vitro*, and then for 1 week on Jiffy pellets. The 5 week-old thalli were grown for 4 weeks *in vitro*, and then for 1 week on Jiffy pellets. Only the tips of the thalli, i.e., the actively growing tissues, were used for the experiments.

### Extraction of Proteins From Cell Walls

About 3.5–4 g of fresh material were used for 2 week-old thalli, 5–7 g for 3 week-old thalli and 35–40 g for 5 week-old thalli. Extraction of CWPs started with purification of cell walls as described with slight modifications (Feiz et al., 2006). The plant material was placed on nylon mesh (25 μm pore size) and washed by 2 L of sodium acetate buffer 5 mM pH 4.6, then with 1 L of sodium acetate buffer 5 mM pH 4.6 containing 0.4 M sucrose. The plant material was ground at 4°C for 30 s in a blender containing 400 mL/g fresh material sodium acetate buffer 5 mM pH 4.6 containing 0.4 M sucrose supplemented with 20 mM DTT, 10 mM TCEP [Tris (1-carboxyethyl) phosphine hydrochloride] (Sigma-Aldrich), protease inhibitors cocktail (Sigma-Aldrich) and poly (vinylpolypyrrolidone) (PVPP, Sigma-Aldrich). This step was followed by centrifugation at 30,000 *g* for 15 min at 4°C. It was repeated twice, but successively with sodium acetate buffer 5 mM pH 4.6 containing 0.6 M sucrose and 1 M sucrose. The resulting pellet was washed on nylon mesh (pore size: 25 μm^2^) with 3 L sodium acetate buffer 5 mM pH 4.6. It was finally ground in liquid nitrogen using a mortar with pestle until obtaining a homogeneous fine powder. This fraction, so-called “purified cell walls,” was lyophilized until complete desiccation.

Extraction of proteins was done as described (Irshad et al., 2008). Twelve milliliter of 5 mM sodium acetate buffer at pH 4.6 containing 0.2 M CaCl_2_ were added to 1 g lyophilized purified cell walls together with 9 μL/g protease inhibitors cocktail (Sigma-Aldrich). After 20 min of agitation, the mixture was centrifuged at 45,000 *g* for 30 min at 4°C and the supernatant was collected. The proteins were successively extracted with 5 mM sodium acetate buffer pH 4.6 containing (i) 0.2 M CaCl_2_ (6 mL/g purified cell walls), (ii) 2 M LiCl (10 mL/g purified cell walls) in the presence of 10 μL of protease inhibitors cocktail, and (iii) 2 M LiCl (6 mL/g purified cell walls). All the supernatants were pooled and subjected to a last centrifugation step to eliminate cellular residues. The protein solution was desalted on Econo-Pack^®^ 10 DG columns (BIO-RAD, Hercules, CA, United States) equilibrated with 100 mM ammonium formate buffer. The protein solution was lyophilized and resuspended in deionized water. The proteins were quantified using the BCA method (INTERCHIM UPTIMA, Montluçon, France) and separated by 1 D-SDS PAGE.

### Isolation of *N*-Glycoproteins

This procedure was realized as described (Minic et al., 2007) with some modifications. The thalli (5 g) were frozen in liquid nitrogen and ground in a mortar with a pestle to a fine powder. The resulting powder was suspended in 50 mL of ice-cold extraction buffer (25 mM potassium phosphate buffer pH 7.4, 0.5 M NaCl, 20 mM DTT, 5 mM TCEP (Sigma-Aldrich), 1 g PVPP, 250 μL plant protease inhibitors cocktail (Sigma-Aldrich) and were ground in a mortar with pestle for 5 min on ice. The material was centrifuged at 40.000 *g* for 15 min at 4°C and the proteins were precipitated with ammonium sulfate (80% saturation) to concentrate the protein extract. The pellet was resuspended with 3 mL of ConA buffer (20 mM Tris-HCl pH 7.4, 1 mM CaCl_2_/MnCl_2_/MgCl_2_, 0.5 M NaCl). The proteins were desalted on Econo-Pack 10 DG (BIO-RAD) columns. Then, the protein concentration was measured using the BCA method (INTERCHIM UPTIMA). Five mg of proteins were incubated with 1.5 mL ConA resin (20 mg/mL agarose conjugate) (Sigma-Aldrich) for at least 3 h at 4°C. Then, the ConA suspension was poured into a small plastic disposable column (Thermo Fisher Scientific, Rockford, IL, United States). The column was washed thrice with the ConA buffer, followed by elution with 0.2 M methyl-α-glucopyranose (Sigma-Aldrich) in the same buffer. The proteins were quantified using the BCA method (INTERCHIM UPTIMA) and separated by 1 D-SDS PAGE.

### Analysis of the Proteins by 1D-SDS PAGE

Sixty microgram of each protein extract were loaded on 12% polyacrylamide gel and migrated for 1 h at 35 V. Gels were stained overnight with Coomassie blue PageBlue™ Protein Staining Solution (Thermo Fisher Scientific). Prior to silver staining, this step was followed by washing of gels with UHQ water until the complete disappearance of the blue color. Silver staining was performed as described (Shevchenko et al., 1996).

### Determination of Total Phenolics Content

The extraction procedure was designed after (Wang et al., 2016). One g of lyophilized powdered thalli was extracted with 25 mL 80% ethanol using an ultrasonicator (Elmasonic S ultrasonic, Elma Schmidbauer GmbH, Singen, Germany) for 30 min at 50°C. This step was repeated twice. After each extraction, the solution was vortexed for 10 min in dark. Both extracts were combined and filtered through a 0.45 μm membrane (Merck Millipore, Darmstadt, Germany). The filtrate was evaporated using a rotary evaporator at 55°C for 20 min. The dry residue (crude extract) was dissolved in 80% ethanol. The total phenolics content was determined according to the Folin and Ciocalteu method (Kaur and Kapoor, 2002) with some modifications. Twenty microliter from each sample were added to 1.58 mL deionized water and 100 μL Folin and Ciocalteu’s phenol reagent (Sigma-Aldrich). After 2 min of mixing, 300 μL of sodium carbonate solution (0.2 g/L) were added and the mixture was homogenized. The samples were incubated at 40°C for 30 min. Finally, the absorbance was measured at 765 nm. The total phenolics content expressed as mg gallic acid/g dry mass was determined by comparison to a standard curve determined using various concentrations of gallic acid (0–500 mg/mL).

### Identification of Proteins by Mass Spectrometry

Sixty μg of each protein extract were solubilized in 30 μL Laemmli buffer (Laemmli, 1970). They were vortexed during 3 min prior being heated at 70°C during 5 min. They were shortly separated by 1D-SDS-PAGE at a constant voltage of 200 V during 12 min. The gels were stained with Coomassie blue (BioSafe™, BIO-RAD) according to the purchaser’s instructions except that a step of fixation in 10% acetic acid, 40% ethanol was added before the rinsing step. The gel was cut into three or six pieces for the proteins extracted from purified cell walls, or the *N*-glycoproteins, respectively. Each sample was then digested according to the in-gel digestion protocol described in Supplementary Table 1A. Then, the samples were analyzed by LC-MS/MS following the procedure described in Supplementary Table 1B. The mass spectrometer was a Q-Exactive Plus (Thermo Fisher Scientific) coupled to a NanoLC 425 System (Sciex, Villebon sur Yvette, France). The raw data produced *via* the XCalibur™ 4.0.27.19 software (Thermo Fisher Scientific) were converted using msconvert (Proteowizard 3.0.9576)^2^ before the database search. The V3.1 version of the *M. polymorpha* genomic data was downloaded from Phytozome.^3^ Database search was perform using an in-house database collecting usual contaminants and X!Tandem Piledriver (2015.04.01.1) (Craig and Beavis, 2004; see Supplementary Tables 1B, 2, 3 for details). X!TandemPipeline (Release 0.4.2) (Langella et al., 2017) was used for MS data processing (see Supplementary Table 1B for details). The validation of the identification of a protein was only confirmed when at least two peptides were found with an *e-*value lower than 1.00E-02 and an overall *e*-value for the protein lower than 1.00E-05. Only proteins identified with at least two different specific peptides in the same biological replicate and found in at least two biological replicates were validated. The quantification of the CWPs extracted from the purified cell walls has been performed with MassChroQ (version 2.2.29) as described (Valot et al., 2011) using the XIC (extracted ion chromatogram) values. The MS/MS are publicly available at ProticDB (Langella et al., 2013;^4^ projects “cell_wall_marchantia_proteome” and “cell_wall_marchantia_glycoproteome”). The data related to CWPs can be found at *WallProtDB* (see text footnote 1) (San Clemente and Jamet, 2015).

### Bioinformatics

Two types of bioinformatic predictions have been performed: the sub-cellular localization of proteins and the presence of functional domains using the Pr*otAnnDB* in-house tool^5^ (San Clemente et al., 2009). Proteins having a predicted signal peptide, but lacking intracellular retention motifs or having less than two transmembrane domains have been called CWPs.

## Results

### Solving the Issue of the Presence of High Amounts of Phenolic Compounds in *M. polymorpha* Tissues

**FIGURE 1:**
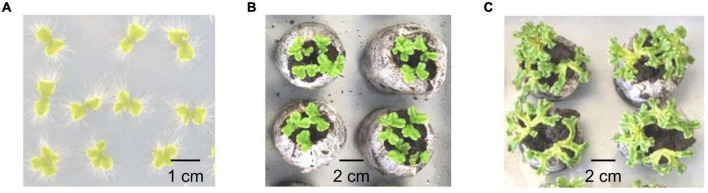
Different stages of development of the *M. polymorpha* thalli used for the proteomics analyses. **(A)** After *in vitro* culture for 2 weeks (2 week-old thalli). **(B)** After *in vitro* culture for 2 weeks, followed by 1 week of culture on soil (3 week-old thalli). **(C)** After *in vitro* culture for 4 weeks, followed by 1 week of culture on soil (5 week-old thalli).

**FIGURE 2:**
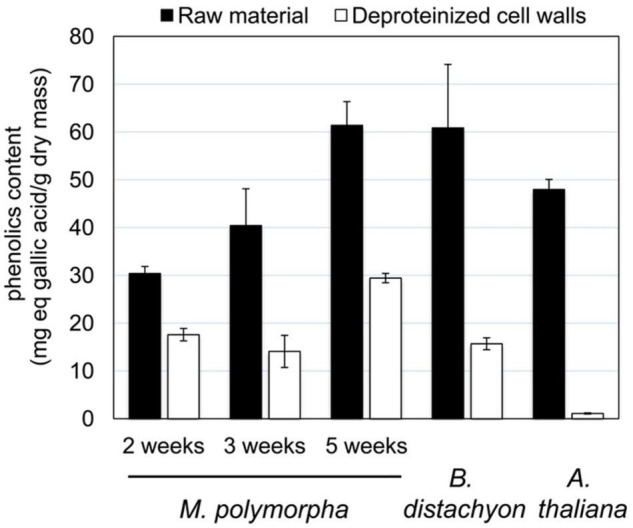
Phenolics content of *M. polymorpha* thalli at different stages of development, compared to those of *B. distachyon* mature leaves and *A. thaliana* rosettes. The phenolics content has been determined as mg eq gallic acid/g dry mass as described in Materials and Methods.

The usual procedure to isolate cell walls starts with a rough grinding of plant tissues (Feiz et al., 2006). At the beginning of our experiments with 5 week-old thalli, we have faced difficulties to avoid strong oxidation reactions which gave a dark brown to nearly black color to the extracts. It was not possible to get rid of these compounds even after gel filtration on Econo-Pack 10 DG columns. The presence of high amounts of phenolics in *M. polymorpha* tissues was previously described (Schwartner et al., 1996; Soriano et al., 2019). The risk was to favor their unspecific oxidative fixation on proteins and thus to prevent the proper extraction and the identification of proteins by MS and bioinformatics. We have quantified these compounds at the three different stages of development of *M. polymorpha* of interest (Figure 1): 2 week-old thalli undergoing active growth and exhibiting scarce rhizoids, 3 week-old thalli representing an intermediate stage of development with more rhizoids, and 5 week-old thalli carrying gemma cups. We have compared the results to those obtained with aerial organs of *A. thaliana* and *B. distachyon* which have both provided easy material for protein preparation as shown in previous studies (Douché et al., 2013; Hervé et al., 2016; Duruflé et al., 2017), The starting material (named “raw material”) was compared to purified deproteinized cell walls (Figure 2). The amount of phenolic compounds correlated with the age of *M. polymorpha* thalli showing an increase by 100% between 5 week-old thalli compared and 2 week-old ones, in both types of materials. By comparison, this amount was similar to that found in the leaves of *B. distachyon* and a little bit higher than that found in *A. thaliana* rosettes. The difference remained high between the values obtained with the purified deproteinized cell walls, especially with those of *A. thaliana*. Since our difficulties appeared to be specific to *M. polymorpha*, we assumed that (i) the phenolics compounds were less easily extracted during the cell wall purification procedure and remained at the subsequent steps of protein extraction and (ii) the oxidation reactions were more important in *M. polymorpha* cell wall extracts. Then, we have modified the cell wall purification procedure by adding an antioxidant, 10 mM TCEP, at the very beginning of the experiment. It should be mentioned that the addition of DTT alone did not allow solving this issue. Although the amount of phenolic compounds could not be reduced, the oxidative reactions could be prevented. The protein extracts then appeared in a light brown color.

### Yield of Protein Extraction and Quality Control

We have used a two-step protocol to prepare the protein extracts from purified cell walls. It includes the preparation of a purified cell wall fraction followed by an extraction of proteins with CaCl_2_ and LiCl salt solutions (Irshad et al., 2008). This means that only proteins weakly interacting or ionically bound to cell wall components have been extracted. The extraction of covalently bound proteins would have required a different strategy such as the use of trypsin on purified cell walls (Chen et al., 2015).

The amount of purified cell walls as well as that of the extracted proteins was quantified (Table 1 and Supplementary Figure 1). The yield of cell wall preparation (mass of purified cell walls per mass of fresh material) strongly increased from 2 to 5 week-old thalli, from 8.8 ± 1.8% to 23.3 ± 5.8%. Conversely, the yield of proteins extracted from cell walls with salt solutions (CaCl_2_ 0.2 M followed by LiCl 2 M) decreased from 2 to 5 week-old thalli, from 90.9 ± 16.2 to 22.3 ± 0.3 μg proteins/g fresh material. Regarding the *N*-glycoproteins purified by affinity chromatography on ConA, the elution of the proteins was checked by 1D SDS-PAGE and three fractions (E1-E3) were retained for further analysis and protein quantification (Supplementary Figure 2). The yield of the purification procedure (mass of total proteins per mass of proteins eluted from the ConA column) was similar at all three stages of development, i.e., it ranged between 11 and 13% of the total protein amount.

**TABLE 1:** Yield of cell wall purification and of proteins extractions.

	2 week-old	3 week-old	5 week-old
Yield of cell wall purification (%) (mass lyophilized cell walls/mass fresh material)	8.8 ± 1.8	9.8 ± 0.9	23.3 ± 5.8
Yield of protein extraction from cell walls (μg proteins/g fresh material)	90.9 ± 16.2	63.8 ± 7.9	22.3 ± 0.3
Yield of total proteins extraction (mg proteins/g fresh material)	1.9 ± 0.3	2.8 ± 0.4	1.7 ± 0.2
Yield of *N*-glycoproteins extraction (μg *N*-glycoproteins/μg total proteins)	11.4%	13.1%	10.9%

**FIGURE 3:**
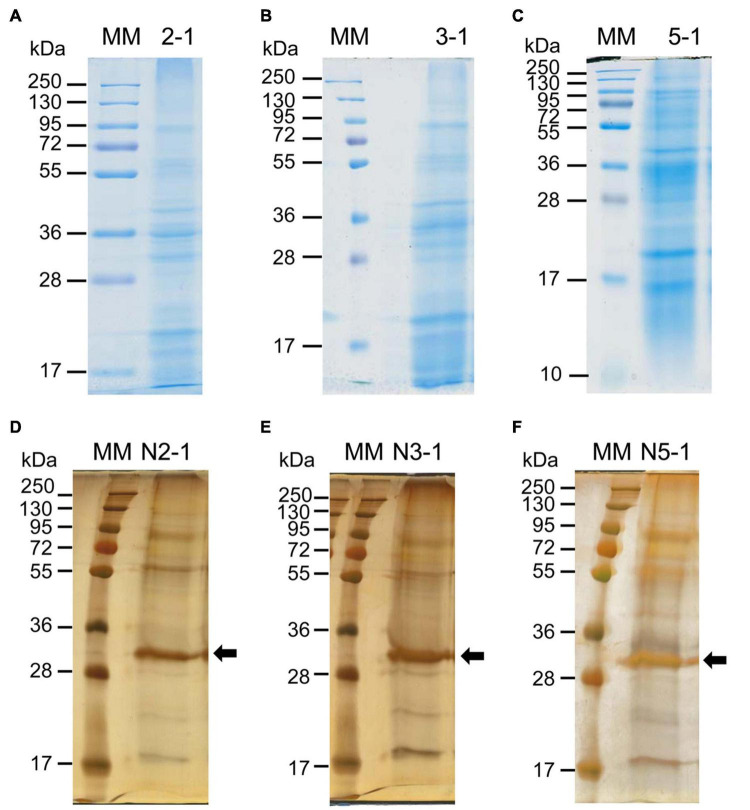
1D-E patterns of proteins eluted from purified cell walls with salt solutions **(A–C)** or of *N*-glycoproteins enriched after ConA affinity chromatography **(D–F)**. Proteins have been eluted from purified cell walls of 2 week- **(A)**, 3 week- **(B)** or 5 week-old thalli **(C)**. A representative biological replicate is shown in each case (2–1, 3–1, and 5–1) and proteins have been stained with Coomassie blue. *N*-glycoproteins have been enriched after ConA affinity chromatography from a total protein extract from 2 week- **(D)**, 3 week- **(E)** or 5 week-old thalli **(F)**. A representative biological replicate is shown in each case (N2-1, N3-1 and N5-1) and proteins have been revealed with Coomassie blue, followed by silver staining. The arrows point at ConA in **(D–F)**. The other biological replicates are shown in Supplementary Figures 2, 4.

The next step was to check the quality of the protein extracts. This was performed by analyzing equivalent amounts of proteins (60 μg) by 1D SDS-PAGE (Figure 3 and Supplementary Figures 3, 4). The patterns showed distinct protein bands, indicating no protein degradation, and they were similar for each set of biological replicates (proteins extracted from purified cell walls and *N*-glycoproteins), indicating a good reproducibility of the experiments (Supplementary Figures 3, 4). Note that it was necessary to stain the *N*-glycoproteins with silver nitrate, probably because the Coomassie blue staining was not efficient due to the *N*-glycosylations. The comparison of the different stages of development showed little variations between the protein patterns (Figure 3). Then, the protein extracts were ready for MS analysis and protein identification. After protein identification, all the identified proteins have been annotated to predict their sub-cellular localization (see section “Materials and Methods,” Supplementary Tables 2, 3). Only the proteins predicted to be secreted and not retained in an intracellular compartment were considered as CWPs. Based on XIC values, 191 out of the 385 CWPs extracted from purified cell walls could be quantified. A principal component analysis showed the good grouping of the three biological replicates for each developmental stage (Supplementary Figure 5).

The number of predicted *N*-glycosylation sites was determined for each set of CWPs to assess the efficiency of the ConA affinity chromatography (Supplementary Figure 6). The percentage of proteins with no predicted *N*-glycosylation sites was much higher for the CWPs extracted from purified cell walls (17 vs. 4%), than for the CWPs purified by ConA affinity chromatography. In addition, 36% of the CWPs extracted from cell walls contained one or two predicted *N*-glycosylated sites, whereas 35% of the CWPs purified by affinity chromatography on ConA contained two or three predicted *N*-glycosylated sites. These results showed a clear enrichment in *N*-glycosylated proteins for the CWPs purified by ConA affinity chromatography.

### Comparison Between the Proteins Extracted From the Purified Cell Walls and the *N*-Glycoproteins Purified by ConA Affinity Chromatography

Altogether, 1239 different proteins were identified in the protein extracts from purified cell walls among which 376 (30.3%) were predicted to be CWPs (Table 2). The total number of identified proteins was the highest in the 2 and 3 week-old samples (966 and 942, respectively) and the lowest in the 5 week-old sample (764). Conversely, the proportion of CWPs was the lowest in the 2 and 3- week-old samples (29.5 and 28.3%, respectively) and the highest in the 5 week-old sample (43.1%).

**TABLE 2:** Number of proteins identified in *M. polymorpha* thalli at three stages of development (2, 3, or 5 week-old thalli).

	2 week-old	3 week-old	5 week-old	Altogether*^a^*
Proteins extracted from cell walls	966	942	764	1,239
Intracellular proteins	684	675	434	863
CWPs*^b^*	282 (29.2%)	267 (28.3%)	330 (43.2%)	376 (30.3%)
*N*-glycoproteins	246	322	279	427
Intracellular proteins	75	160	112	206
CWPs*^b^*	171 (69.5%)	162 (50.3%)	167 (59.8%)	221 (51.7%)

*The detailed data are available in Supplementary Tables 2, 3.*

*^a^The numbers correspond to those of unique proteins.*

*^b^The numbers between brackets correspond to the percentage of CWPs among the identified proteins.*

The number of identified *N*-glycoproteins was lower with 427 different proteins, but the proportion of CWPs was higher (51.7%). The comparison between both proteomes showed that 189 proteins were identified in both of them whereas 35 CWPs were only identified in the *N*-glycoproteome (Supplementary Figure 6). Overall, 410 different CWPs were identified.

### The Main Cell Wall Proteins Families of the *M. polymorpha* Cell Wall Proteome

**FIGURE 4:**
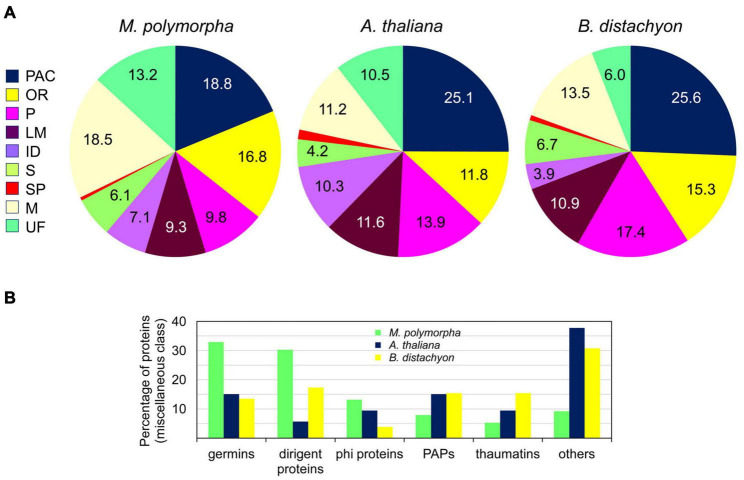
Comparison between the cell wall proteomes of *M. polymorpha* thalli (this study) and the previously published leaves cell wall proteomes of *A. thaliana* and *B. distachyon* (http://www.polebio.lrsv.ups-tlse.fr/WallProtDB/). Overview showing the distribution in functional classes **(A)**. A focus on the miscellaneous class of proteins pointing at a few protein families well represented in the *M. polymorpha* proteome **(B)**. PAC stands for proteins acting on carbohydrates, OR for oxido-reductases, P for proteases, PAPs for purple acid phosphatases, phi proteins for phosphate-induced proteins, LM for proteins possibly involved in lipid metabolism, ID for proteins with interaction domains, S for signaling, SP for structural proteins, M for miscellaneous and UF for proteins of yet unknown function. Detailed data are provided in Supplementary Tables 2, 3.

The 410 CWPs were distributed into the nine functional classes described in the Introduction according to the presence of predicted functional domains (Jamet et al., 2008; see section “Materials and Methods,” Supplementary Tables 2, 3). Considering the weak contribution of rhizoids and scales to the material analyzed compared to thalli, we have made comparisons with the already described cell wall proteomes of *A. thaliana* and *B. distachyon* leaves which have be obtained using the same protocol (see *WallProtDB*; see text footnote 1). Several features could be highlighted (Figure 4A): (i) the proportion of PAC was lower in *M. polymorpha* thalli than in *A. thaliana* and *B. distachyon* leaves (18.8 vs. 25.1 and 25.6%); the proportion of oxido-reductases (OR) was close to that found in *B. distachyon*, but higher than in *A. thaliana* (16.8 and 15.3 vs. 11.8%); the proportion of proteases (P) was lower than in *A. thaliana* and *B. distachyon* (9.8 vs. 13.9 and 17.4%); the proportion of proteins of unknown function (UF) was higher in *M. polymorpha* (13.2 vs. 10.5 and 6.0%); as well as that of M (18.5 vs. 11.2 and 13.5%).

In the PAC functional class, the major proteins families were: (i) the glycoside hydrolases (GH) with five GH16 (endoxyloglucan transferases), 15 GH17 (b-1,3-glucosidases), five GH18 (chitinases), seven GH19 (chitinases/lyzozymes), three GH5 (endo-b-1,4-glucanase), and two GH28 (polygalacturonases); (ii) the expansins (7); and (iii) the carbohydrate esterases (CEs) with six pectin methylesterases (PMEs, CE8) and two pectin acylesterases (PAEs, CE13). Among the OR, class III peroxidases (CIII Prxs) were predominant with 39 identifications, whereas 12 polyphenol oxidases were identified. The major proteins of the proteins related to lipid metabolism (LM) functional class were the lipases acylhydrolases of the GDSL family (23) whereas the non-specific lipid transfer proteins (nsLTPs) were less represented (4). Besides, most of the identified lectins were predicted to bind D-mannose (eight out of 12). Among the signaling proteins (S functional class), six fasciclin arabinogalactan proteins were identified. A closer look at the miscellaneous (M) functional class revealed the presence of a large proportion of germins (25), dirigent-like proteins (23) and phosphate-induced 1 proteins (phi-1/EXORDIUM-like) (10) compared to *A. thaliana* and *B. distachyon*, but a lower proportion of purple acid phosphatases (PAPs) (6) and thaumatins (4) (Figure 4B). Finally, 52 proteins with yet unknown functions were identified.

### Variations in the Cell Wall Proteome During the Time Course of the Experiment

The cell wall proteomes obtained at the three developmental stages were compared: 2 week-old young actively growing thalli exhibited scarce rhizoids and scales whereas the 5 week-old thalli showed more rhizoids, more scales and gemma cups. It should be noted that only the tips of the thalli were analyzed, i.e., the tissues undergoing active growth. The quantitative data were used to refine the pattern of accumulation whenever possible (see Supplementary Figures 7–9 for a full description, and Supplementary Figure 10 for a few protein families).

**FIGURE 5:**
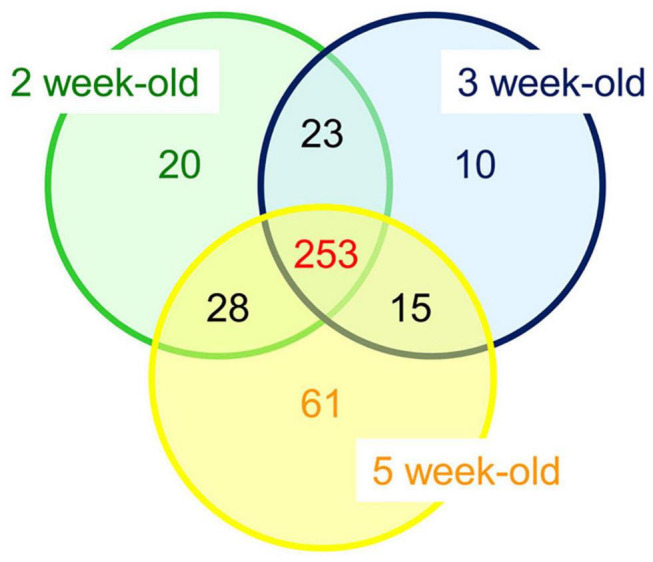
Overall comparison between the cell wall proteomes of *M. polymorpha* thalli at three different stages of development (2, 3, or 5 week-old thalli). Detailed data are provided in Supplementary Tables 2, 3.

**FIGURE 6:**
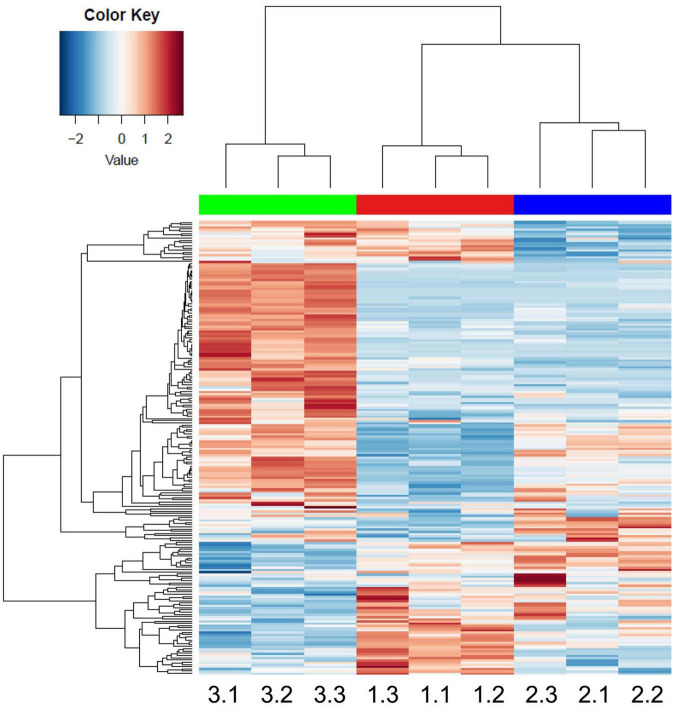
Heatmap performed with the MS XIC quantitative data. The samples are named as follows: 1.1, 1.2, and 1.3 stand for the three biological replicates of 2 week-old thalli; 2.1, 2.2, and 2.3 for the three biological replicates of 3 week-old thalli; and 3.1, 3.2, and 3.3 for the three biological replicates of 5 week-old thalli. The names of the genes can be found in Supplementary Figure 7.

Altogether, 253 CWPs were common to the three developmental stages, 20 were specific to the 2 week-old thalli (“early proteome”), 10 to the 3 week-old, and 61 to the 5 week-old (“late proteome”) (Figure 5). Overall, the distribution of the CWPs in functional classes was not modified between the three stages of development (not shown). To refine this comparison, a quantitative analysis was performed using the three biological replicates corresponding to CWPs. A heatmap calculated with the 191 CWPs which were quantified clearly showed the grouping of the biological replicates for each developmental stage (Figure 6 and Supplementary Figure 7). For each of the three developmental stages, a specific pattern of CWPs accumulation could be found. The cell wall proteomes of 2 and 3 week-old thalli segregated drastically from that of 5 week-old thalli. The latter broadly exhibited an opposite profile compared to that of 2 week-old thalli with the CWPs accumulated at a low level becoming accumulated at a high level, and vice versa. Six clusters of CWPs could be identified depending of their kinetics of accumulation: increasing or decreasing level of accumulation, transient increase or decrease (Supplementary Figure 8).

**FIGURE 7:**
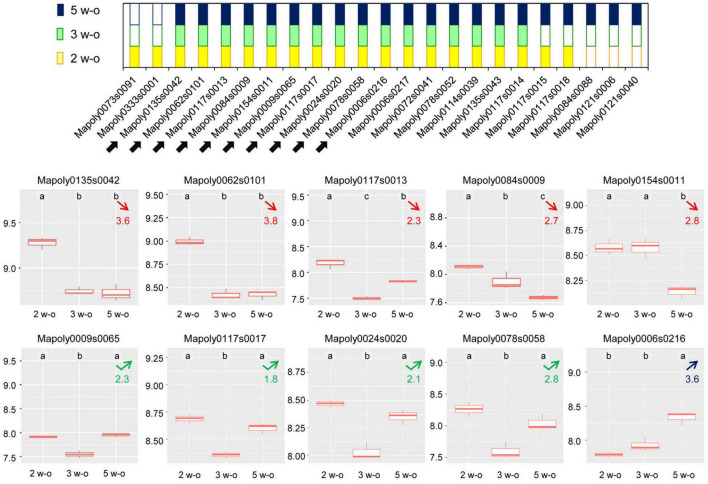
Distribution of the dirigent-like proteins between the three stages of development (2, 3, or 5 week-old thalli). The upper panel shows the developmental stages in which the proteins have been identified: yellow squares correspond to 2 week-old thalli (2 w-o), green squares to 3 week-old thalli (3 w-o) and blue squares to 5 week-old thalli (5 w-o). The lower panels provide the quantitative data for the ten dirigent proteins which could be quantified (highlighted by arrows in the upper panel). The calculations have been done using MS XIC (extracted ion chromatogram) quantitative data. The data are expressed as log_10_. The statistical analysis is according to Tukey’s test. Numbers in red indicate a decrease in protein abundance at the 5 week-old compared to the 2 week-old developmental stage and corresponds to the ratio of protein abundance between 2 and 5 week-old samples. The number in blue indicates an increase in protein abundance at the 5 week-old compared to the 2 week-old developmental stage and corresponds to the ratio of protein abundance between 5 and 2 week-old samples. Numbers in green correspond to a transient decrease in protein abundance at the 3 week-old developmental stage and corresponds to the ratio of protein abundance between 5 and 3 week-old samples.

For different protein families, the distribution of the members followed a similar pattern as shown in Figure 7 for the dirigent-like protein family: some members were only present at 2 weeks, or gradually disappeared; others remained present during the time course of the experiment; and others were only identified at the latest stage of development. Other examples of this typical pattern are shown in Supplementary Figure 10 for the following protein families: GH17, D-mannose-binding lectins, GDSL lipases/acylhydrolases, phi-1/EXORDIUM-like proteins, CIII Prx, polyphenol oxidases, and germins.

Based on the number of specific peptides, a short list of the most abundant CWPs at the three stages of development could be established (Table 3). It comprises the only identified GH3 (exo-acting β-D-glucosidase), three PMEs, three CIII Prxs, four polyphenol oxidases, one PAP and one protein of yet unknown function. These proteins could perform particular basic functions in *M. polymorpha* cell walls.

**TABLE 3:** Major CWPs identified in *M. polymorpha* thalli at the three stages of development.

Accession number^a^	Annotation according to functional domains^b^
Mapoly0014s0035	Glycoside hydrolase family 3—GH3
Mapoly0007s0111	Carbohydrate esterase family 8—CE8 (pectin methylesterase—PME)
Mapoly0028s0010	Carbohydrate esterase family 8—CE8 (pectin methylesterase—PME)
Mapoly0055s0109	Carbohydrate esterase family 8—CE8 (pectin methylesterase—PME)
Mapoly0064s0029	CIII peroxidase (MpPrx182)
Mapoly0008s0265	CIII peroxidase (MpPrx185)
Mapoly0048s0002	CIII peroxidase (MpPrx95)
Mapoly0071s0078	Polyphenol oxidase
Mapoly3313s0001	Polyphenol oxidase
Mapoly0032s0137	Polyphenol oxidase
Mapoly0084s0086	Polyphenol oxidase domain
Mapoly0045s0021	Berberine-bridge oxido-reductase-like (BBE-like)
Mapoly0212s0012	Lectin (D-mannose-binding)
Mapoly0013s0084	Ser peptidase (subtilisin family) (Peptidase family S8)
Mapoly0003s0101	Purple acid phosphatase
Mapoly0160s0014	Protein of yet unknown function

*The quantification data are available in Supplementary Figure 8 and Supplementary Table 2.*

*^a^The accession numbers refer to the v3.1 version of the M. polymorpha genome (https://phytozome.jgi.doe.gov/pz/portal.html#!info?alias=Org_Mpolymorpha).*

*^b^The annotation of proteins is based on the presence of functional domains as described in Materials and methods.*

### Evaluation of the Coverage of the *M. polymorpha* Cell Wall Proteome

In this study, we could identify 410 CWPs of *M. polymorpha*. To evaluate the coverage of the whole expected cell wall proteome, we have determined the size of a few gene families using the corresponding PFAM entries^6^ and we have compared it to the number of identified CWPs (Table 4). For three largest gene families (CIII Prx, germins and polyphenol oxidases, 189, 73, and 59 members respectively), the percentage of coverage was between 20 and 25%. For the other protein families, it was between 30 and 55%. Altogether, we can evaluate the coverage of the expected *M. polymorpha* cell wall proteome around 33%. The missing proteins could be (i) accumulated at other stages of development like during the reproductive cycle, (ii) present in minute amount and thus not detected by the used methods, or (iii) lost or not extracted with the used methods.

**TABLE 4:** Evaluation of the coverage of the *M. polymorpha* cell wall proteome.

Protein family^a^	Number of genes^b^	Number of identified CWPs
GH5 (PF00150)	9	3 (33%)
GH17 (PF00332)	27	15 (55%)
GH18 (PF00704)	10	5 (50%)
GH19 (PF00182)	15	7 (47%)
GH28 (PF00295)	7	2 (29%)
GH38 (PF01074, PF07748)	3	1 (33%)
CIII Prxs (PF02704)*^c^*	189	39 (21%)
PMEs (PF01095)	14	6 (43%)
PAEs (PF03283)	6	2 (33%)
Polyphenol oxidases (PF12142)	59	12 (20%)
GDSL lipases/acylhydrolases (PF00657)	49	23 (47%)
Lectins (D-mannose-binding) (PF01453)	26	8 (31%)
Dirigent-like proteins (PF03018)	54	23 (43%)
Germins (PF00190)	73	25 (33%)
EXORDIUM-like proteins (PF04674)	18	10 (54%)
PAPs (PF00149)	11	6 (55%)

*^a^The PFAM entry (http://xfam.org/) corresponding to each protein family is indicated between brackets. GH stands for glycoside hydrolase, CIII Prx for class III peroxidase, PME for pectin methylesterase, PAE for pectin acylesterase, PAP for purple acid phosphatase.*

*^b^The PFAM entries have been used to mine the Phytozome database and determine the number of genes in each protein family (https://phytozome.jgi.doe.gov/pz/portal.html#!info?alias=Org_Mpolymorpha).*

*^c^The Redoxibase was used as a reference (https://peroxibase.toulouse.inrae.fr/).*

## Discussion

This study has allowed the identification of 410 different CWPs of *M. polymorpha* by combining the results of the analysis of three development stages of the haploid gametophyte and of two different isolation methods. We are aware that the *N*-glycoproteome is probably missing proteins carrying complex or asymmetric *N*-glycans which are not recognized by ConA. However, it allows enlarging the *M. polymorpha* cell wall proteome and showing the presence of *N*-glycoproteins in its cell walls. Besides, the covalently bound proteins could not be extracted with the used protocol. Overall, this work shows the high dynamics of the cell wall proteome during the development of *M. polymorpha* thalli. All the identified CWPs could be classified in the same nine functional classes as those of flowering plants, thus showing the conservation of many biological activities in the cell wall during the evolution of the green lineage. However, some specific features could be highlighted, such as the importance of D-mannose binding lectins, GDSL lipases/acylhydrolases, germins, dirigent-like proteins, and of oxido-reductases like CIII peroxidases and polyphenol oxidases. The following discussion will be focused on these protein families as well as on some PAC.

To date, the best covered cell wall proteome is that of *A. thaliana* with about half of the predicted CWPs already identified at least in one cell wall proteome (see *WallProtDB*; see text footnote 1). An estimation of the coverage of the *M. polymorpha* cell wall proteome could be done by comparing the number of identified CWPs and that of genes predicted to encode CWPs. Depending on the family of CWPs, this coverage varies from 20 to 55% with an average of 33%. The less covered protein families are the largest ones, such as CIII Prxs (189 members), and polyphenol oxidases (59 members). Contrarily, the best covered are the GH17, GH18, GH19, GDSL lipases/acylhydrolases, phi-1/EXORDIUM-like proteins and PAPs with around half of their members identified. The different results observed between the protein families could be related to the roles they play either during the development of the gametophyte (our study), that of the sporophytes, or in response to environmental cues. They could also be due to a tissue-specific expression pattern. As mentioned, the main represented tissues in the analyzed samples are those of the thallus proper, the rhizoids and the scales being only weakly represented. By comparison, half of the *A. thaliana* CIII Prxs were identified in the root cell wall proteome (Nguyen-Kim et al., 2016).

Several families of CWPs involved in the remodeling of the cell wall polysaccharides networks (PAC) have been identified. Fifteen GH17 having a β-1,3-glucosidase activity could be involved in the hydrolysis of callose which has been found in the cell plates of all land plants (Scherp et al., 2001). The high number of GH17 could be related to the active cell divisions supporting the rapid growth of *M. polymorpha* thalli. Three GH5 and five GH16 having, respectively, a trans-β-mannanase and an endotransglucosylase activity were identified (Franková and Fry, 2013). Their substrates in cell walls are assumed to be hemicelluloses like xyloglucans or mannans and they are known to play roles in polysaccharides rearrangements during cell growth. The interactions between cellulose microfibrils and hemicelluloses also need to be relaxed during cell wall expansion thanks to the activity of expansins (Cosgrove, 2018). The identification of two GH28, six PMEs and two PAEs is consistent with the presence of galacturonic acid (GalA) in the acidic hydrolysate of *M. polymorpha* cell wall polysaccharides. Indeed, these three CWP families have as a substrate the homogalacturonan which is a polymer of α-1,4-D-GalA as a substrate (Hocq et al., 2017). One may thus expect the presence of demethylesterified homogalacturonans in *M. polymorpha* cell walls. Finally, five GH18 and seven GH19 have been identified. They have been shown to exhibit chitinase and chitinase/lysozyme activities, respectively (Tyler et al., 2010). They have anti-fungal and anti-bacterial activities and are involved in defense reactions against biotic and abiotic stresses.

Several protein families are known to be involved in oxido-reduction reactions involving aromatic compounds in *M. polymorpha* cell walls. The antagonistic enzymatic activities of CIII Prxs allow them to participate in cell wall remodeling events in two opposite ways (Francoz et al., 2015): (i) they play a role in cell wall loosening by generating reactive oxygen species (ROS) able to cut non-enzymatically the cell wall polysaccharides; or (ii) they can participate in the cell walls stiffening by oxidizing aromatic compounds such as aromatic amino acids, monolignols or cinnamic acid in the presence of H_2_O_2_, or in the cross-linking of structural proteins like extensins, thus forming covalent networks. The case of polyphenol oxidases is more puzzling. This is the first time that such proteins are identified in cell wall proteomes. Considering the presence of aromatic compounds in Bryophyte cell walls, they could be involved in their polymerization. Indeed p-coumaric acid, ferulic acid, as well as the anthocyanidins, riccionidin A and riccionidin B have been found, but no lignin (Kuntz et al., 1994; Soriano et al., 2019). In addition, lignans have been found although their precise sub-cellular localization has not been precisely determined (Cullmann and Becker, 1989; Scher et al., 2003). Lignans have been assumed to be localized in vacuoles and/or in the cell wall (Wang et al., 2013b). Besides nearly half of the expected dirigent proteins have been identified. These proteins have been assumed to be involved in lignin and in lignan biosynthesis by controlling the regio- and stereoselectivity of bimolecular phenoxy radical coupling (Paniagua et al., 2017). Finally, one laccase and several multicopper oxidases have been found. Similarly to CIII Prxs, laccases have been shown to be involved in the lignification process. An *A. thaliana* mutant impaired in the *LACCASE5* gene was shown to have a 10% reduction in lignin content, and a modification of the ratio of the syringyl-to-guaiacyl units (Wang et al., 2015). The *A. thaliana sku5* mutant impaired in the *SKEWED5* gene encoding a GPI-anchored multicopper oxidase shows directional root growth defects and *SKEWED5* was assumed to play a role in root expansion (Sedbrook et al., 2002).

The acquisition of a cuticle has been a major event in land plant colonization. Twenty-three GDSL lipases/acylhydrolases have been identified in the *M. polymorpha* thalli. A GDSL lipase/acylhydrolase has been shown to be involved in cuticle formation. In a *GDSL1*-silenced tomato plants, a significant reduction the thickness of the fruit cuticle was observed as well as a decrease in the content in cutin monomers proportional to the level of *GDSL1* silencing (Girard et al., 2002). Similar results were obtained with the tomato *cd1* (*cutin deficiency 1*) mutant impaired in the same gene and the encoded protein was shown to be extracellular and have a cutin synthase activity *in vitro* (Yeats et al., 2012). In contrast, only four nsLTPs were identified. LTPs have been assumed to play roles in the transfer of lipid molecules through the hydrophilic cell wall thanks to their hydrophobic pockets or to maintain the integrity of the interface between the cell wall proper and the cuticle (DeBono et al., 2009; Jacq et al., 2017).

Eight proteins predicted to be D-mannose binding lectins were identified in the *M. polymorpha* thalli and their number and the amount of each of them increased at the latest stage of development when gemma cups were differentiated. A few D-mannose binding lectins have been identified the cell wall proteomes of *Medicago sativa* stems (Verdonk et al., 2012; Printz et al., 2015), in the *N*-glycoproteome of the tomato fruit pericarp (Catalá et al., 2011), and in the xylem sap of *Gossypium hirsutum* (Zhang et al., 2015). The abundance of such proteins in the *M. polymorpha* cell wall proteome could be linked to the presence of high amounts of mannans as suggested by the presence of significant amount of mannose residues in pectins- and hemicelluloses-enriched cell walls extracts (Happ and Classen, 2019). The presence of mannans was also shown in several Bryophytes by paper chromatography after acid hydrolysis of cell wall alcohol insoluble residues (Popper and Fry, 2003). Mannans are the main hemicelluloses in Charophytes, but have been replaced by other hemicelluloses in spermatophytes (Scheller and Ulvskov, 2010). In flowering plants, they are assumed to play roles in carbohydrate storage and in leaf folding upon drought stress as in *Aloe* species (Scheller and Ulvskov, 2010; Ahl et al., 2019). D-mannose binding lectins could also interact with high mannose-type *N*-glycans of *N*-glycoproteins. They could also play roles in defense reaction by interacting with carbohydrates originating from cell wall polysaccharide degradation or from pathogen structures (Lannoo and Van Damme, 2014; Breitenbach Barroso Coelho et al., 2018). However, the precise role of cell wall lectins is still unknown.

Several protein families are particularly well represented among the miscellaneous CWPs and we will only focus on a few of them. The biological activity of germins and germin-like proteins is still a matter of debate. Initially identified in cereals, they have then been found in many plants (Dunnwell et al., 2008; Breen and Bellgard, 2010). They share the presence of cupin domain and of a so-called germin box with conserved amino acid domains. Three types of enzymatic activities have been associated to germins: oxalate oxidase (OXO), manganese superoxide dismutase (SOD) or ADP glucose pyrophosphatase or phosphodiesterase (AGPPase). A role of the *Craterostigma plantagineum* germin-like protein CpGLP1 in the integrity of the plant in response to drought stress has been assumed through the control of the ROS metabolism (Giarola et al., 2020). However, other activities have been suspected for germins like a role in plant defense against biotic and abiotic stresses (Dunnwell et al., 2008; Wang et al., 2013a). It is thus not possible to assign a biological activity to those identified in the *M. polymorpha* proteome without any experimental work. Besides, numerous phi-1/EXORDIUM-LIKE proteins have been identified in this work. A tobacco *phi-1* gene was first identified among those induced after recovery from phosphate-induced starvation of cell suspension cultures (Sano et al., 1999). The *phi-1* gene was later on found to be homologous to the *A. thaliana EXORDIUM* gene which is expressed in proliferating cells (Ferrar et al., 2003). A more recent study has shown that the overexpression of the *Eucalyptus globulus EgPHI-1* gene enhances the tolerance of transgenic tobacco plants to osmotic stress (Sousa et al., 2014).

## Conclusion

Altogether, the cell wall proteome of *M. polymorpha* exhibit common characteristics with those of flowering plants, but also some specificities which could be related to the cell wall composition, with for example the presence of mannans and aromatic compounds. D-mannose-binding lectins and polyphenol oxidases are among the specific CWP families identified. Many proteins of yet unpredictable function were identified, which opens new perspectives to discover new functions in the Bryophyte cell walls. Besides, the overall coverage of the *M. polymorpha* cell wall proteome was estimated to correspond to one third of the expected CWPs. Increasing this coverage will require studying the sporophyte phase of development, focusing on rhizoids and scales which are only present in minute amount in the presently analyzed samples and exploring defense reactions.

## Data Availability Statement

The original contributions presented in the study are publicly available. This data can be found here: The CWP data can be found at WallProtDB (http://www.polebio.lrsv.ups-tlse.fr/WallProtDB/index.php). The mass spectrometry data can be found at ProticDB (http://moulon.inra.fr/protic/cell_wall_marchantia_proteome; http://moulon.inra.fr/protic/cell_wall_marchantia_glycoproteome).

## Author Contributions

HK: experimental investigations. TB: mass spectrometry analyses. JC: *M. polymorpha* cultures. MZ: supervision of mass spectrometry analyses. HC: supervision of the biochemistry work. EJ: supervision, data curation, original draft preparation, and Funding. All authors have contributed to the writing of the manuscript and approved its final version.

## Conflict of Interest

The authors declare that the research was conducted in the absence of any commercial or financial relationships that could be construed as a potential conflict of interest.

## Publisher’s Note

All claims expressed in this article are solely those of the authors and do not necessarily represent those of their affiliated organizations, or those of the publisher, the editors and the reviewers. Any product that may be evaluated in this article, or claim that may be made by its manufacturer, is not guaranteed or endorsed by the publisher.

## Footnotes

1
http://www.polebio.lrsv.ups-tlse.fr/WallProtDB/

2
http://proteowizard.sourceforge.net/tools.shtml

3
https://phytozome.jgi.doe.gov/pz/portal.html#!info?alias=Org_Mpolymorpha

4
http://moulon.inra.fr/protic/

5
http://www.polebio.lrsv.ups-tlse.fr/ProtAnnDB/

6
http://xfam.org
