# Supplementary material for: The Cell Wall Proteome of Marchantia polymorpha Reveals Specificities Compared to Those of Flowering Plants
Source: Front Plant Sci. 2022 Jan 13;12:765846. doi: 10.3389/fpls.2021.765846 (PMC8792609; doi:10.3389/fpls.2021.765846)
Supplement: Supplementary file 1 [file Data_Sheet_1.PDF]

Kolkas *et al.*
Supplementary Figures 1-6, 8, 10

Figure 1. Yields of cell wall purification and of protein extraction with salt solutions from purified cell walls.

Figure 2. Enrichment in *N*-glycoproteins from a total proteins extract after ConA affinity chromatography.

Figure 3. 1D-E analysis of proteins extracted from cell walls.

Figure 4. 1D-E analysis of the *N*-glycoproteins-enriched fractions.

Figure 5. Principal component analysis (PCA) of the MS XIC (extracted ion chromatogram) quantitative data after normalization.

Figure 6. Comparison between the cell wall proteome and the *N*-glycoproteome.

Figure 8. Clustering of proteins according to variation in the MS XIC quantitative data.

Figure 10. Distribution of proteins belonging to different protein families between the three stages of development (2, 3 or 5 week-old thalli).

Supplementary Figure 1. Yields of cell wall purification and of protein extraction with salt solutions from purified cell walls.

A. Cell wall yield: mass of purified cell walls/mass of fresh material
B. Protein yield: amount of proteins (µg)/mass of fresh material

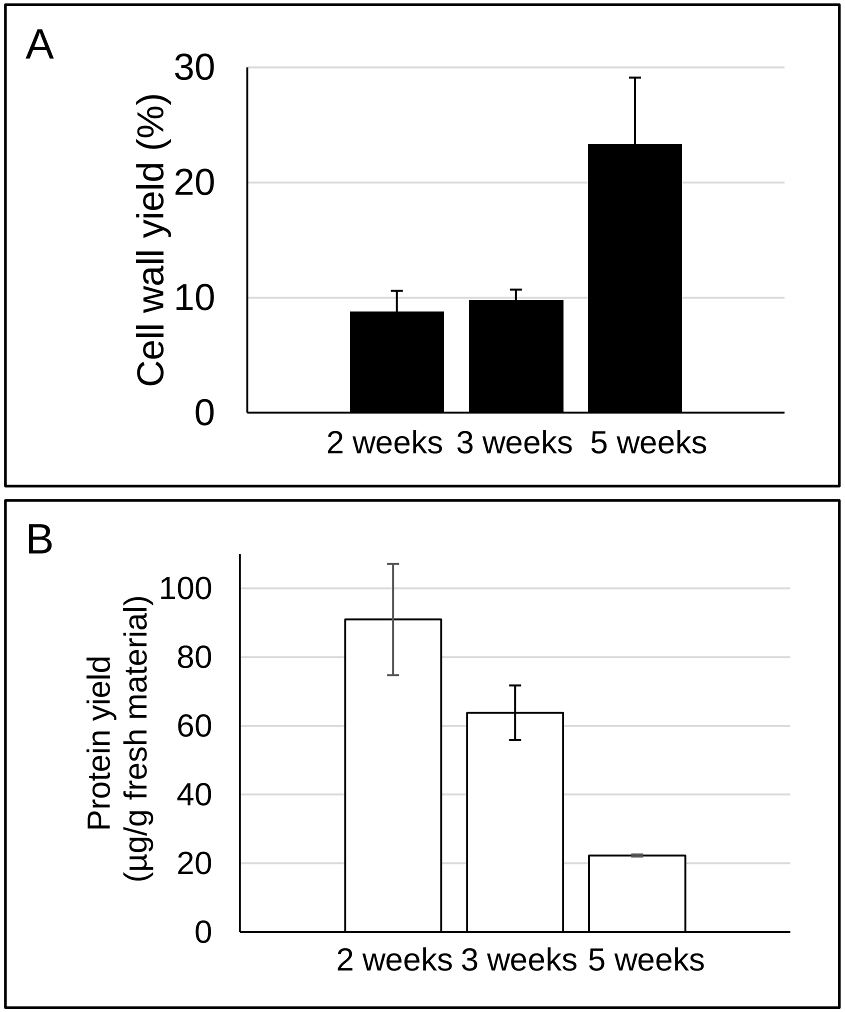

Supplementary Figure 2. Enrichment in *N*-glycoproteins from a total proteins extract after ConA affinity chromatography.

Purification of *N*-glycoproteins by affinity chromatography on Concanavalin A of proteins extracted from 2 week-old thalli. Five mg of total proteins extract (TPE) have been loaded on the ConA column. The effluent (Eff) has been collected. Three successive washes have been performed (W1-3). The *N*-glycoproteins have been eluted in three 1 mL-fractions (E1-3). The proteins have been separated by 1D-E and successively stained with Coomassie blue and silver nitrate. The arrows indicate the presence of ConA.

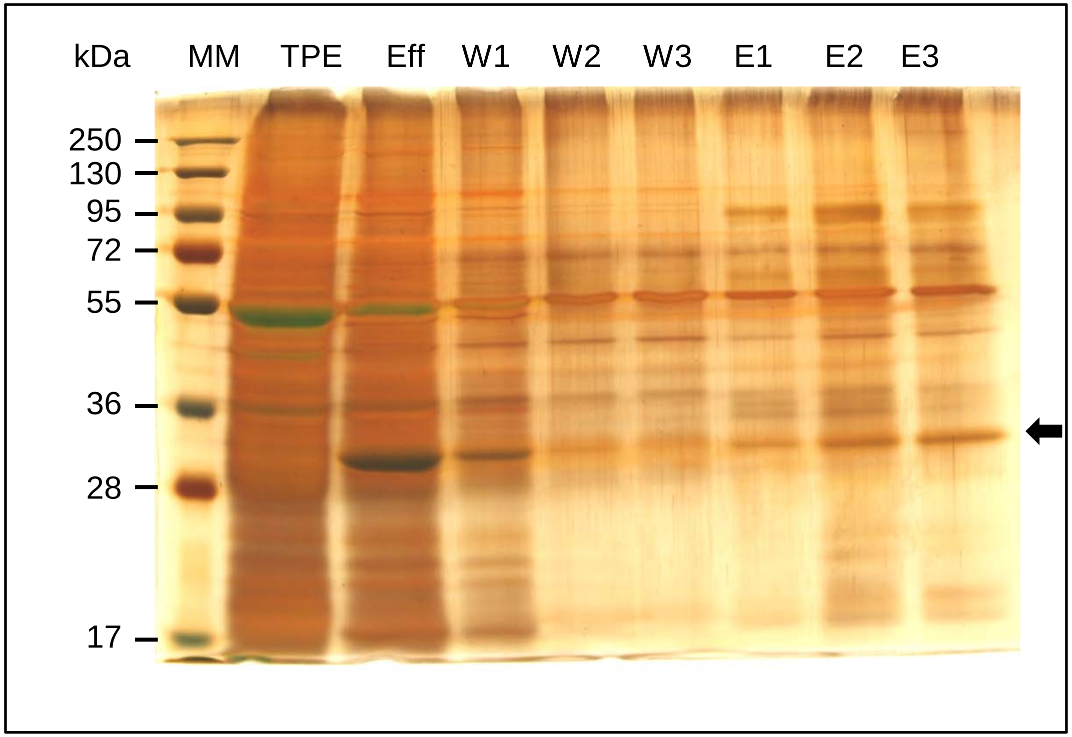

Supplementary Figure 3. 1D-E analysis of proteins extracted from cell walls.

The proteins have been extracted from purified cell walls with salt solutions (CaCl2 0.2 M and LiCl 2 M). Sixty µg of each sample have been loaded on a polyacrylamide gel and separated by 1D-E. The proteins have been stained with Coomassie blue. 2-1, 2-2 and 2-3 stand for 2 week-old biological replicates 1,2 and 3; 3-1, 3-2 and 3-3 for 3 week-old biological replicates; and 5-1, 5-2 and 5-3 for 5 week-old biological replicates.

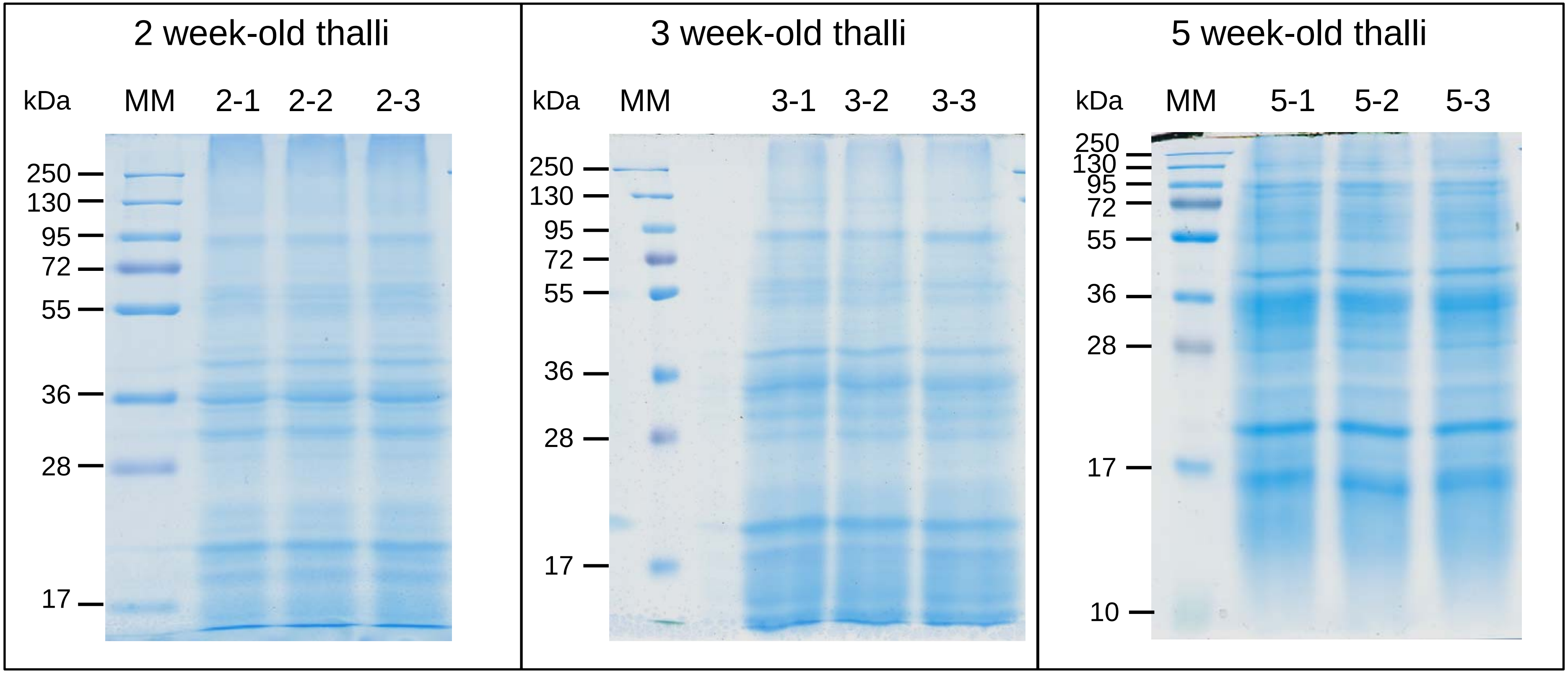

Supplementary Figure 4. 1D-E analysis of the *N*-glycoproteins-enriched fractions.

The total proteins extracts from *M. polymorpha* thalli have been enriched in *N*-glycoproteins by affinity chromatography on Con A. Sixty µg of each sample have been loaded on a polyacrylamide gel and separated by 1D-E. The proteins have been successively stained with Coomassie blue and silver nitrate. N2-1, N2-2 and N2-3 stand for 2 week-old biological replicates 1,2 and 3; N3-1, N3-2 and N3-3 for 3 week-old biological replicates; and N5-1, N5-2 and N5-3 for 5 week-old biological replicates. The arrows indicate the presence of ConA.

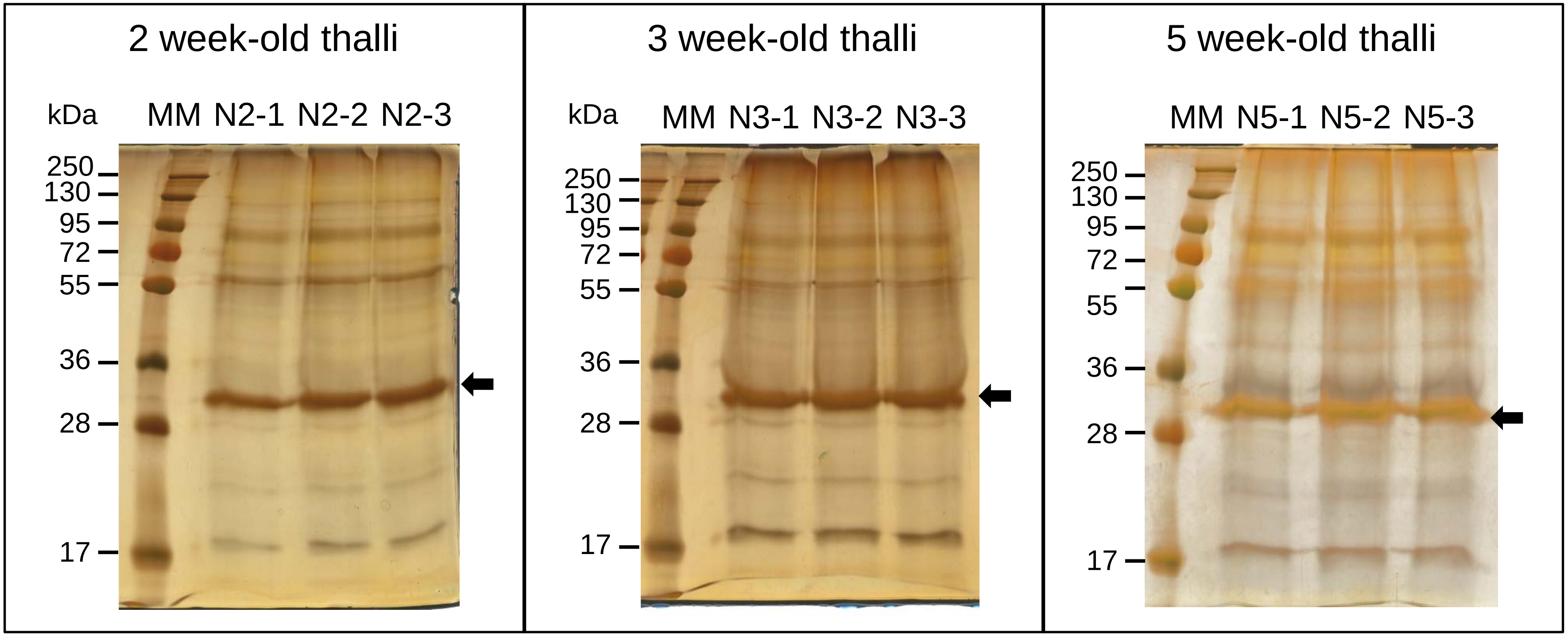

Supplementary Figure 5. Principal component analysis (PCA) of the MS XIC (extracted ion chromatogram) quantiative data after normalization.

A. PCA according to axis 1 and axis 2 explaining 48.2% and 28.4% of the variability between the samples, respectively.

B. PCA according to axis 2 and axis 3 explaining 28.4% and 8.8% of the variability between the samples, respectively.

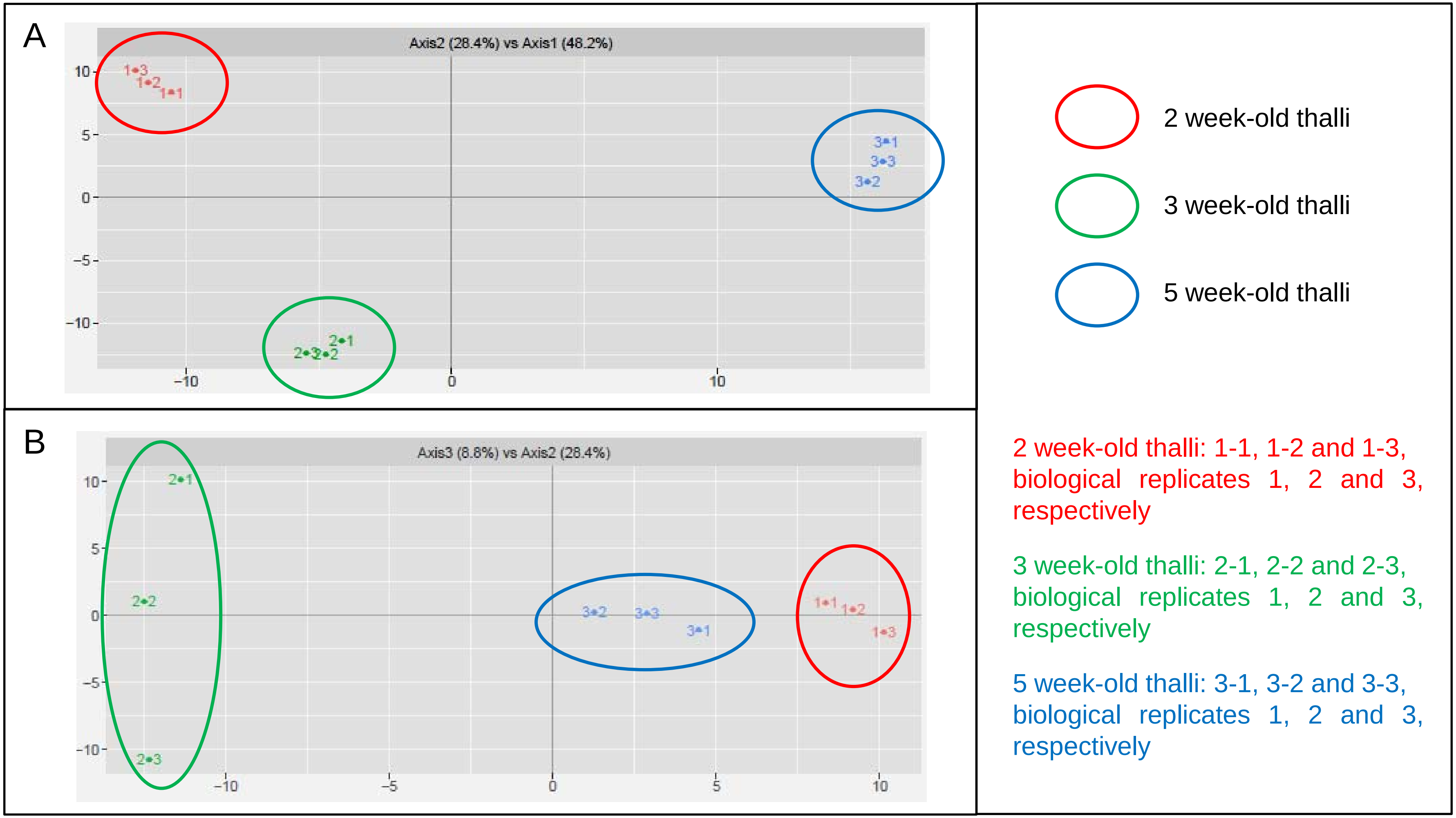

Supplementary Figure 6. Comparison between the cell wall proteome and the *N*-glycoproteome.

A. Overall comparison of the two proteomes

B. Number of *N*-glycans per protein. The arrows indicate the top of each curve.

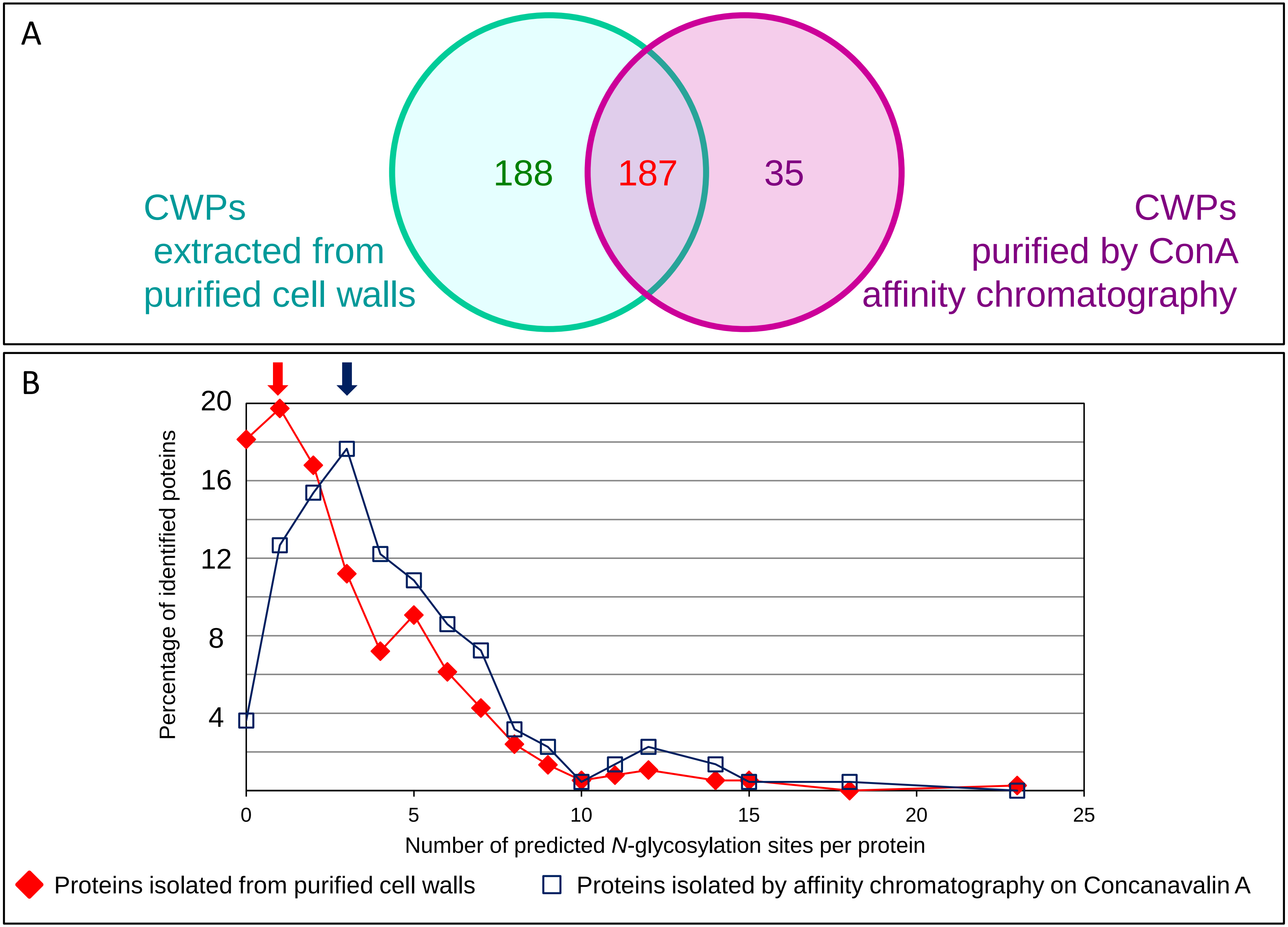

Supplementary Figure 8. Clustering of proteins according to variations in the MS XIC quantitative data.

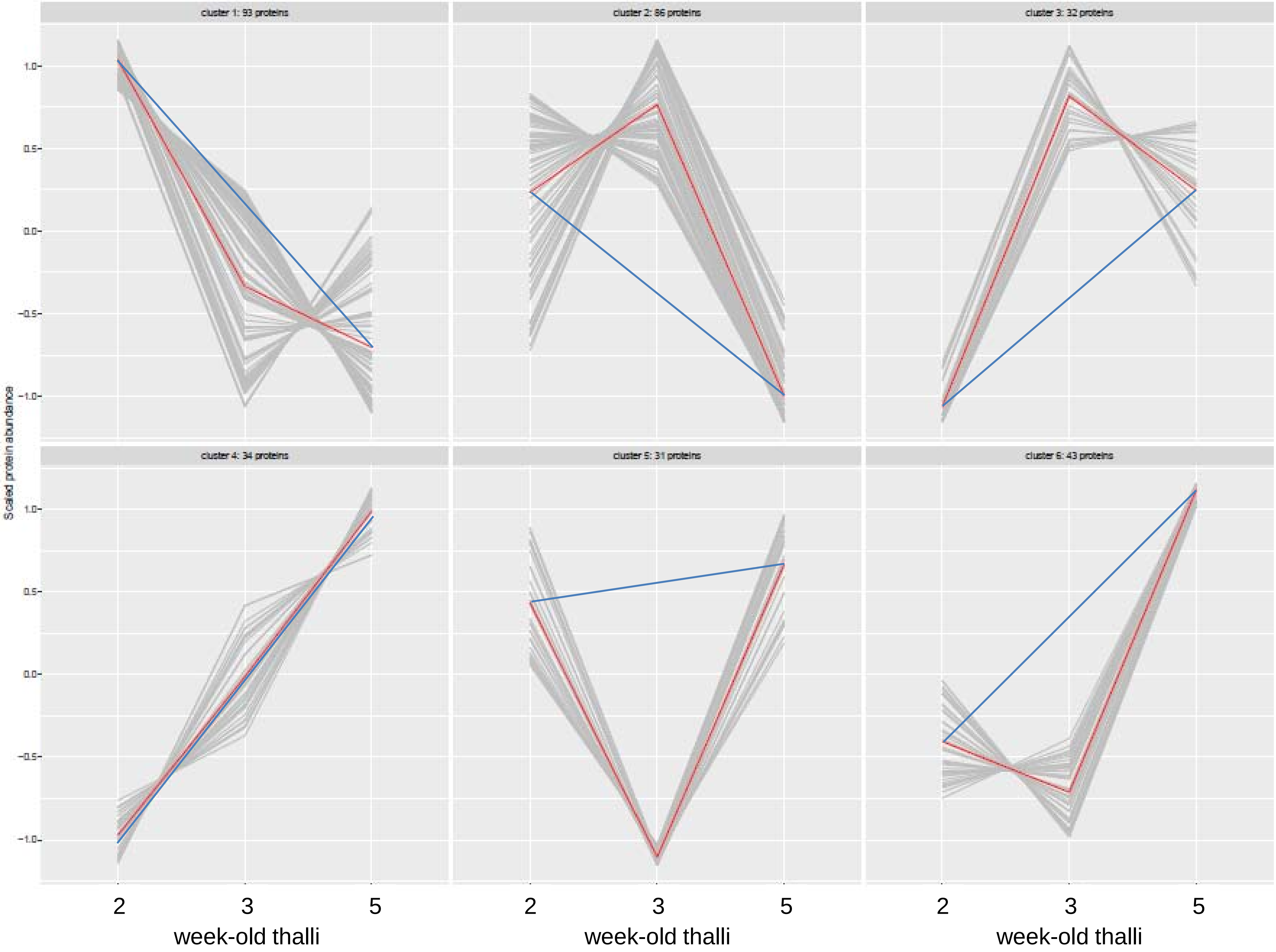

Supplementary Figure 10. Distribution of proteins belonging to different protein families between the three stages of development (2, 3 or 5 week-old thalli). Yellow squares indicate that the protein has been identified in 2 week-old thalli, green squares in 3 week-old thalli and blue squares in 5 week-old thalli. Quantitative data are indicated when available (see Supplementary Figure 8). Numbers in red correspond to the ratio of protein abundance between 2 and 5 week-old samples, whereas numbers in blue correspond to the ratio of protein abundance between 5 and 2 week-old samples. Number in green correspond to the ratio of protein abundance between 5 and 3 week-old samples.

For quantitative data:
Fold increase: 5 week-old/2 week-old ↗
Fold decrease: 2 week-old/5 week-old ↘
Transient decrease (when the ratio 2 week-old/5 week-old is close to 1): 5 week-old/ 3 week-old ↘↗
Transient increase (when the ratio 2 week-old/5 week-old is close to 1): 3 week-old/ 5 week-old ↗↘

A. GH17 / Lectins (D-mannose-binding)
B. GDSL lipases/acylhydrolases / Phosphate-induced (phi) proteins - EXORDIUM-like
C. Class III peroxidases / Polyphenol oxidases
D. Germins

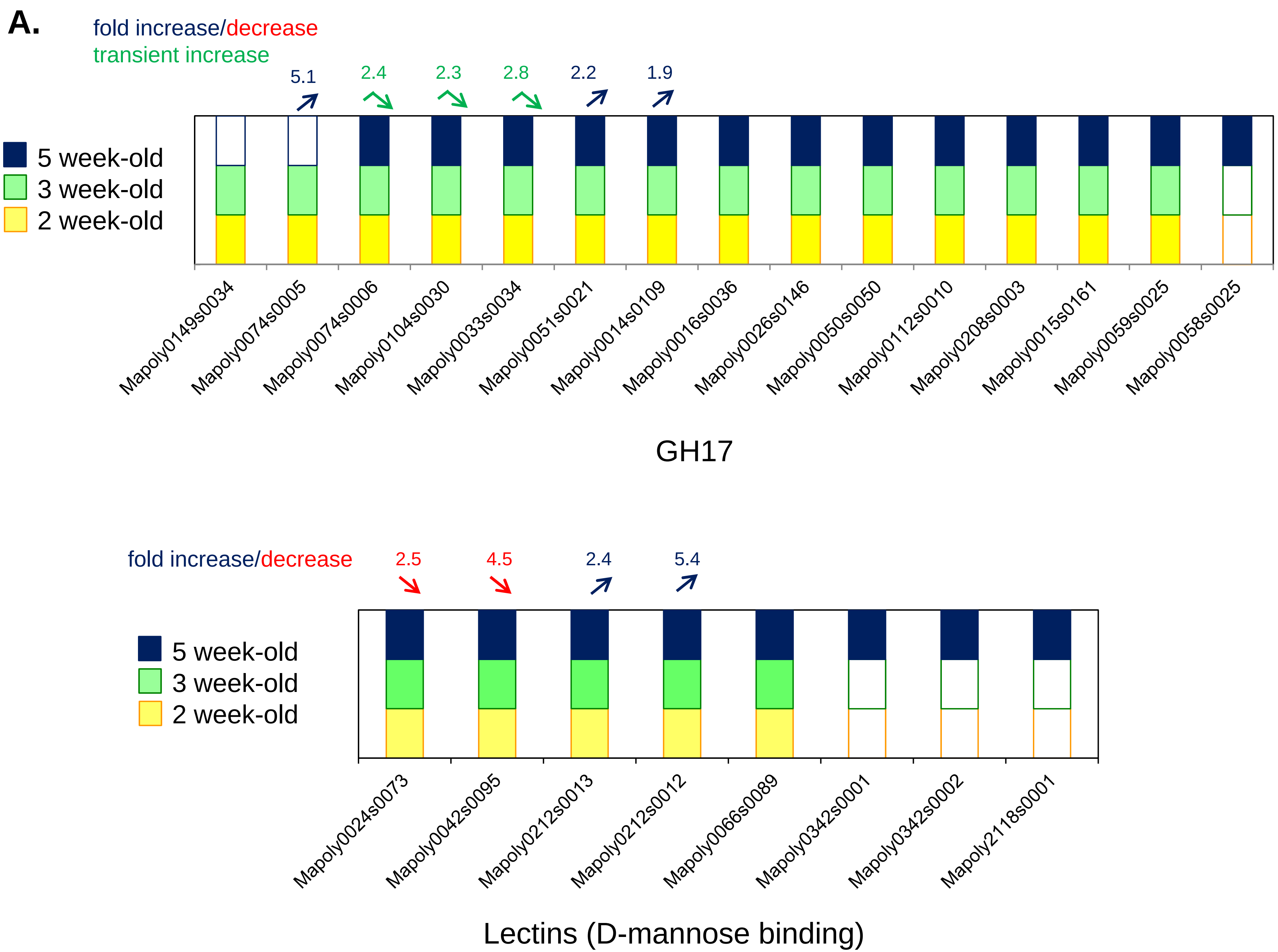
A.
fold increase/decrease
transient increase
5.1
2.4
2.3
2.8
2.2
1.9
5 week-old
3 week-old
2 week-old
Mapoly0149s0034
Mapoly0074s0005
Mapoly0074s0006
Mapoly0104s0030
Mapoly0033s0034
Mapoly0051s0021
Mapoly0014s0109
Mapoly0016s0036
Mapoly0026s0146
Mapoly0050s0050
Mapoly0112s0010
Mapoly0208s0003
Mapoly0015s0161
Mapoly0059s0025
Mapoly0058s0025
GH17
fold increase/decrease
2.5
4.5
2.4
5.4
5 week-old
3 week-old
2 week-old
Mapoly0024s0073
Mapoly0042s0095
Mapoly0212s0013
Mapoly0212s0012
Mapoly0066s0089
Mapoly0342s0001
Mapoly0342s0002
Mapoly2118s0001
Lectins (D-mannose binding)

B.

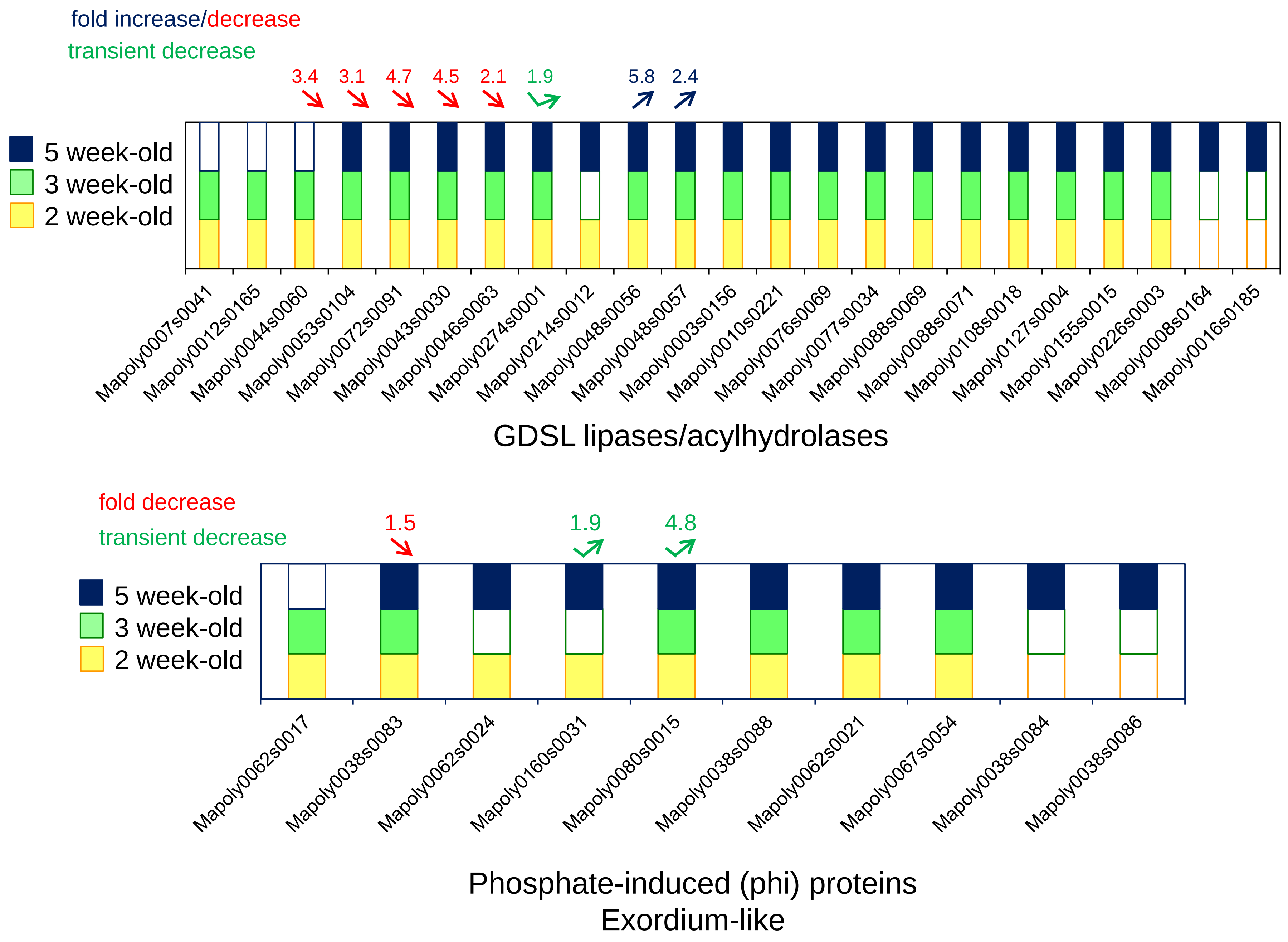
fold increase/decrease
transient decrease
3.4
3.1
4.7
4.5
2.1
1.9
5.8
2.4
5 week-old
3 week-old
2 week-old
Mapoly0007s0041
Mapoly0012s0165
Mapoly0044s0060
Mapoly0053s0104
Mapoly0072s0091
Mapoly0043s0030
Mapoly0046s0063
Mapoly0274s0001
Mapoly0214s0012
Mapoly0048s0056
Mapoly0048s0057
Mapoly0003s0156
Mapoly0010s0221
Mapoly0076s0069
Mapoly0077s0034
Mapoly0088s0069
Mapoly0088s0071
Mapoly0108s0018
Mapoly0127s0004
Mapoly0155s0015
Mapoly0226s0003
Mapoly0008s0164
Mapoly0016s0185
GDSL lipases/acylhydrolases
fold decrease
transient decrease
1.5
1.9
4.8
5 week-old
3 week-old
2 week-old
Mapoly0062s0017
Mapoly0038s0083
Mapoly0062s0024
Mapoly0160s0031
Mapoly0080s0015
Mapoly0038s0088
Mapoly0062s0021
Mapoly0067s0054
Mapoly0038s0084
Mapoly0038s0086
Phosphate-induced (phi) proteins
Exordium-like

C.

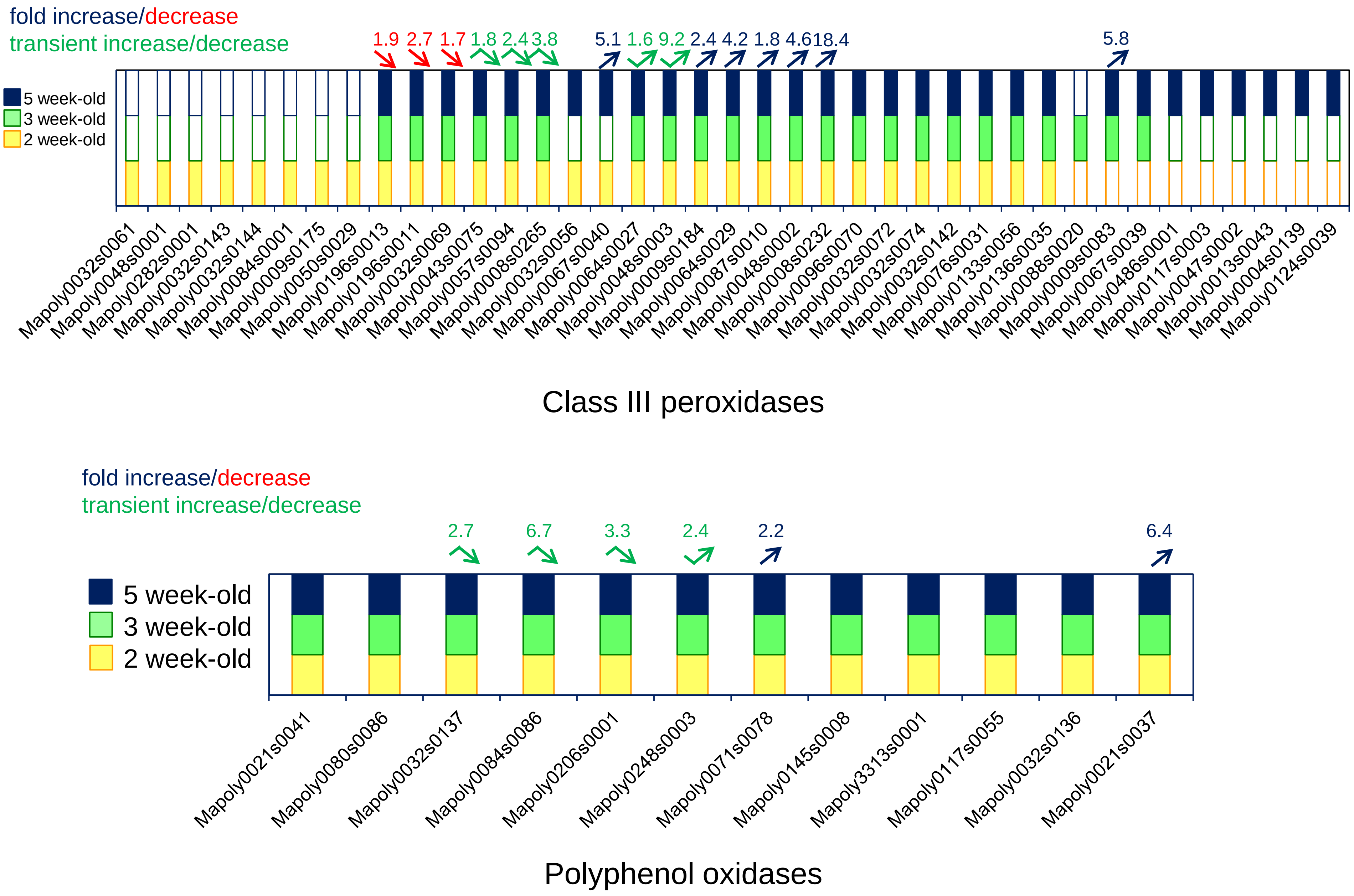
fold increase/decrease
transient increase/decrease
1.9 2.7 1.7 1.8 2.4 3.8 5.1 1.6 9.2 2.4 4.2 1.8 4.6 18.4 5.8
5 week-old
3 week-old
2 week-old
Mapoly0032s0061
Mapoly0048s0001
Mapoly0282s0001
Mapoly0032s0143
Mapoly0032s0144
Mapoly0084s0001
Mapoly0009s0175
Mapoly0050s0029
Mapoly0196s0013
Mapoly0196s0011
Mapoly0032s0069
Mapoly0043s0075
Mapoly0057s0094
Mapoly0008s0265
Mapoly0032s0056
Mapoly0067s0040
Mapoly0064s0027
Mapoly0048s0003
Mapoly0009s0184
Mapoly0064s0029
Mapoly0087s0010
Mapoly0048s0002
Mapoly0008s0232
Mapoly0096s0070
Mapoly0032s0072
Mapoly0032s0074
Mapoly0032s0142
Mapoly0076s0031
Mapoly0133s0056
Mapoly0136s0035
Mapoly0088s0020
Mapoly0009s0083
Mapoly0067s0039
Mapoly0486s0001
Mapoly0117s0003
Mapoly0047s0002
Mapoly0013s0043
Mapoly0004s0139
Mapoly0124s0039
Class III peroxidases
fold increase/decrease
transient increase/decrease
2.7 6.7 3.3 2.4 2.2 6.4
5 week-old
3 week-old
2 week-old
Mapoly0021s0041
Mapoly0080s0086
Mapoly0032s0137
Mapoly0084s0086
Mapoly0206s0001
Mapoly0248s0003
Mapoly0071s0078
Mapoly0145s0008
Mapoly3313s0001
Mapoly0117s0055
Mapoly0032s0136
Mapoly0021s0037
Polyphenol oxidases

D.

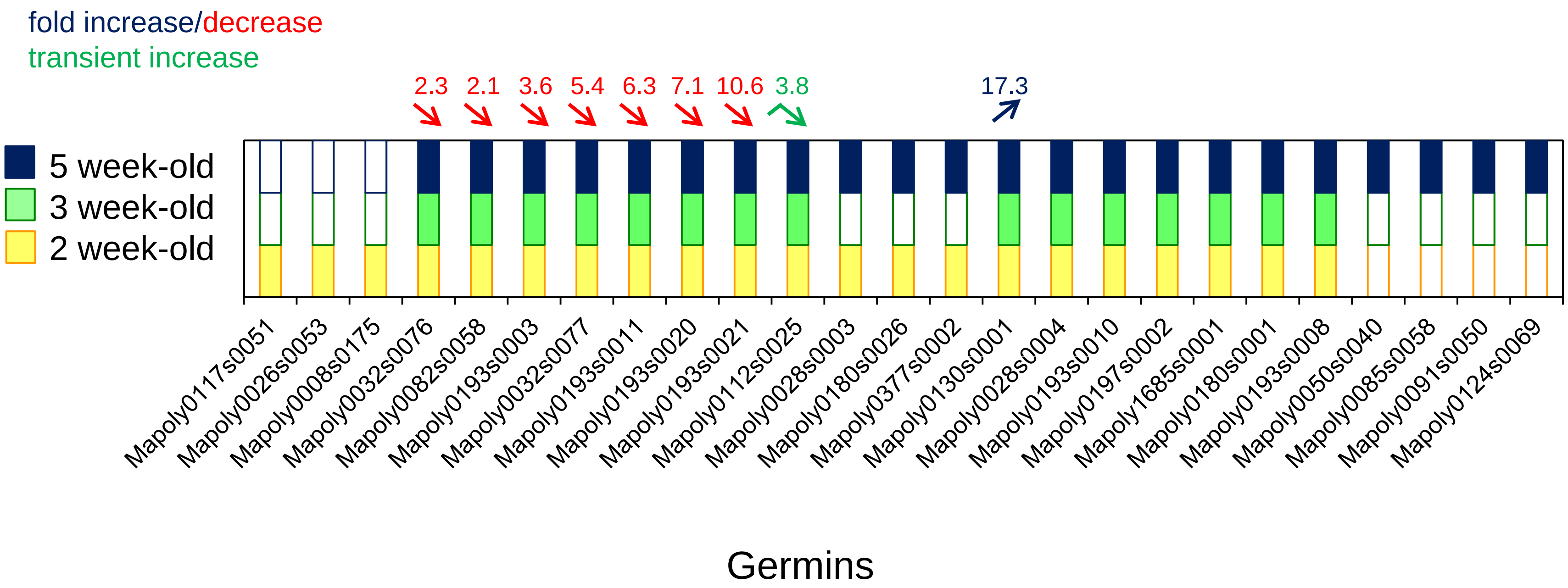

fold increase/decrease
transient increase
2.3
2.1
3.6
5.4
6.3
7.1
10.6
3.8
17.3
5 week-old
3 week-old
2 week-old
Mapoly0117s0051
Mapoly0026s0053
Mapoly0008s0175
Mapoly0032s0076
Mapoly0082s0058
Mapoly0193s0003
Mapoly0032s0077
Mapoly0193s0011
Mapoly0193s0020
Mapoly0193s0021
Mapoly0112s0025
Mapoly0028s0003
Mapoly0180s0026
Mapoly0377s0002
Mapoly0130s0001
Mapoly0028s0004
Mapoly0193s0010
Mapoly0197s0002
Mapoly1685s0001
Mapoly0180s0001
Mapoly0193s0008
Mapoly0050s0040
Mapoly0085s0058
Mapoly0091s0050
Mapoly0124s0069
Germins
