# Supplementary material for: The Cell Wall Proteome of Marchantia polymorpha Reveals Specificities Compared to Those of Flowering Plants
Source: Front Plant Sci. 2022 Jan 13;12:765846. doi: 10.3389/fpls.2021.765846 (PMC8792609; doi:10.3389/fpls.2021.765846)
Supplement: Supplementary file 2 [file Data_Sheet_2.PDF]

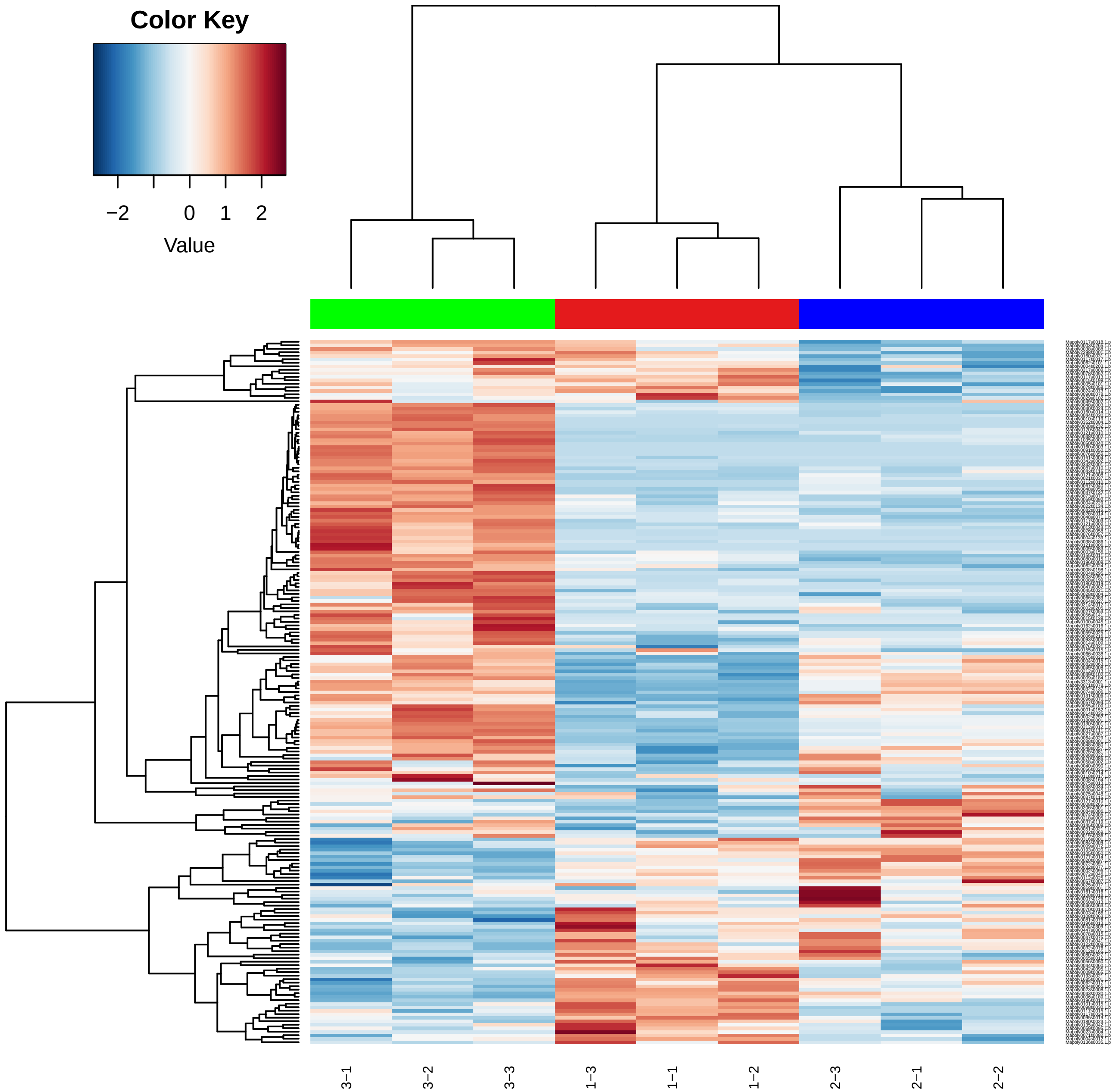

Supplementary Figure 7. Heatmap performed with the MS XIC quantitative data. This picture can be enlarged up to 600 times to read the gene names on the right.
