# Supplementary material for: The Cell Wall Proteome of Marchantia polymorpha Reveals Specificities Compared to Those of Flowering Plants
Source: Front Plant Sci. 2022 Jan 13;12:765846. doi: 10.3389/fpls.2021.765846 (PMC8792609; doi:10.3389/fpls.2021.765846)
Supplement: Supplementary file 3 [file Data_Sheet_3.PDF]

Supplementary Figure 9. Protein abundance plots for 191 CWPs. The calculations have been done using MS XIC (extracted ion chromatogram) quantitative data. The data are expressed as $\log_{10}$. The statistical analysis is according to Tukey's test.

1. Corresponds to 2 week-old thalli
2. Corresponds to 3 week-old thalli
3. Corresponds to 5 week-old thalli

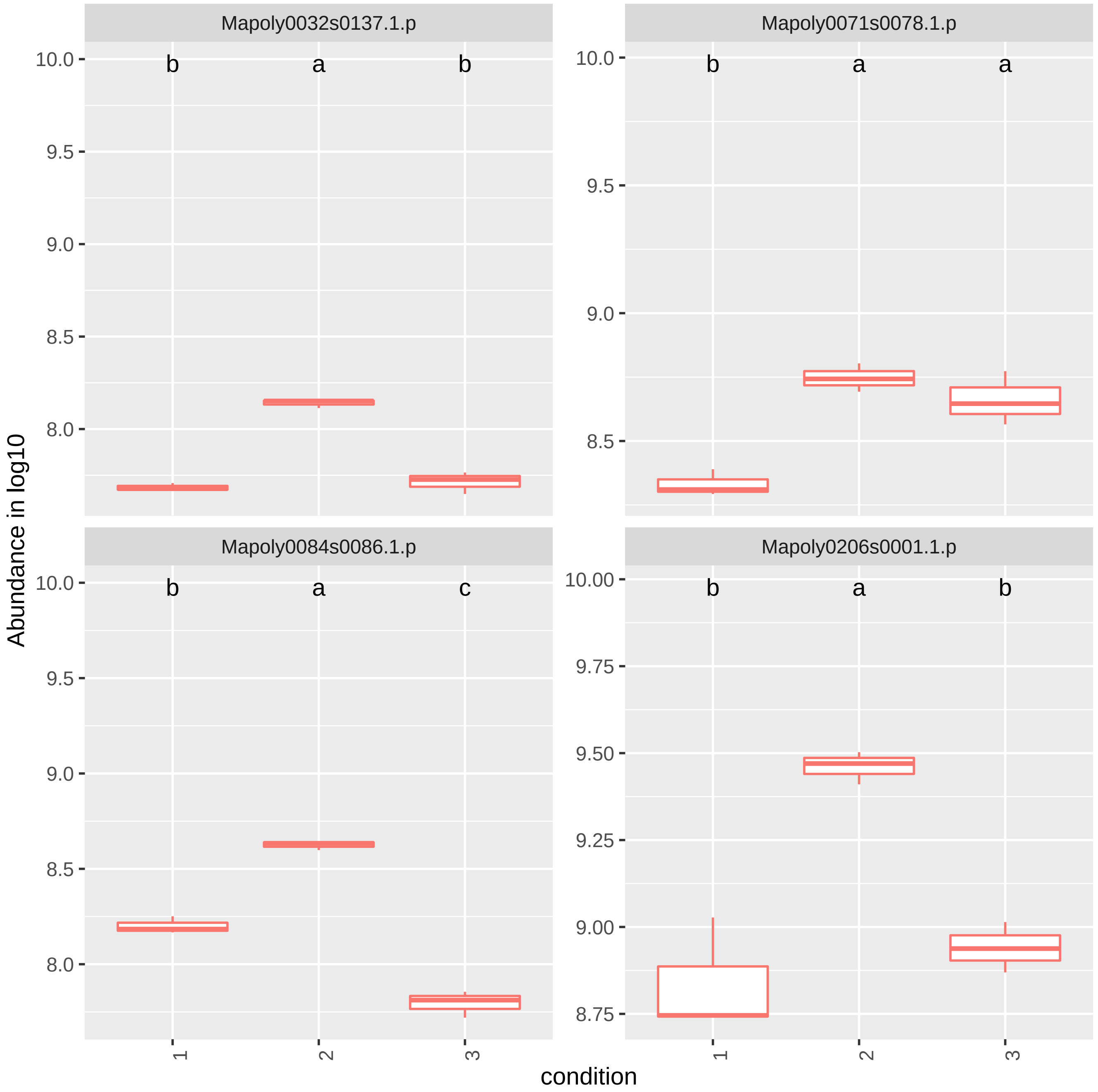

Mapoly0032s0137.1.p
Mapoly0071s0078.1.p
Mapoly0084s0086.1.p
Mapoly0206s0001.1.p
b a b
b a a
b a c
b a b
10.0
9.5
9.0
8.5
8.0
10.00
9.75
9.50
9.25
9.00
8.75
1
2
3
Abundance in log10
condition

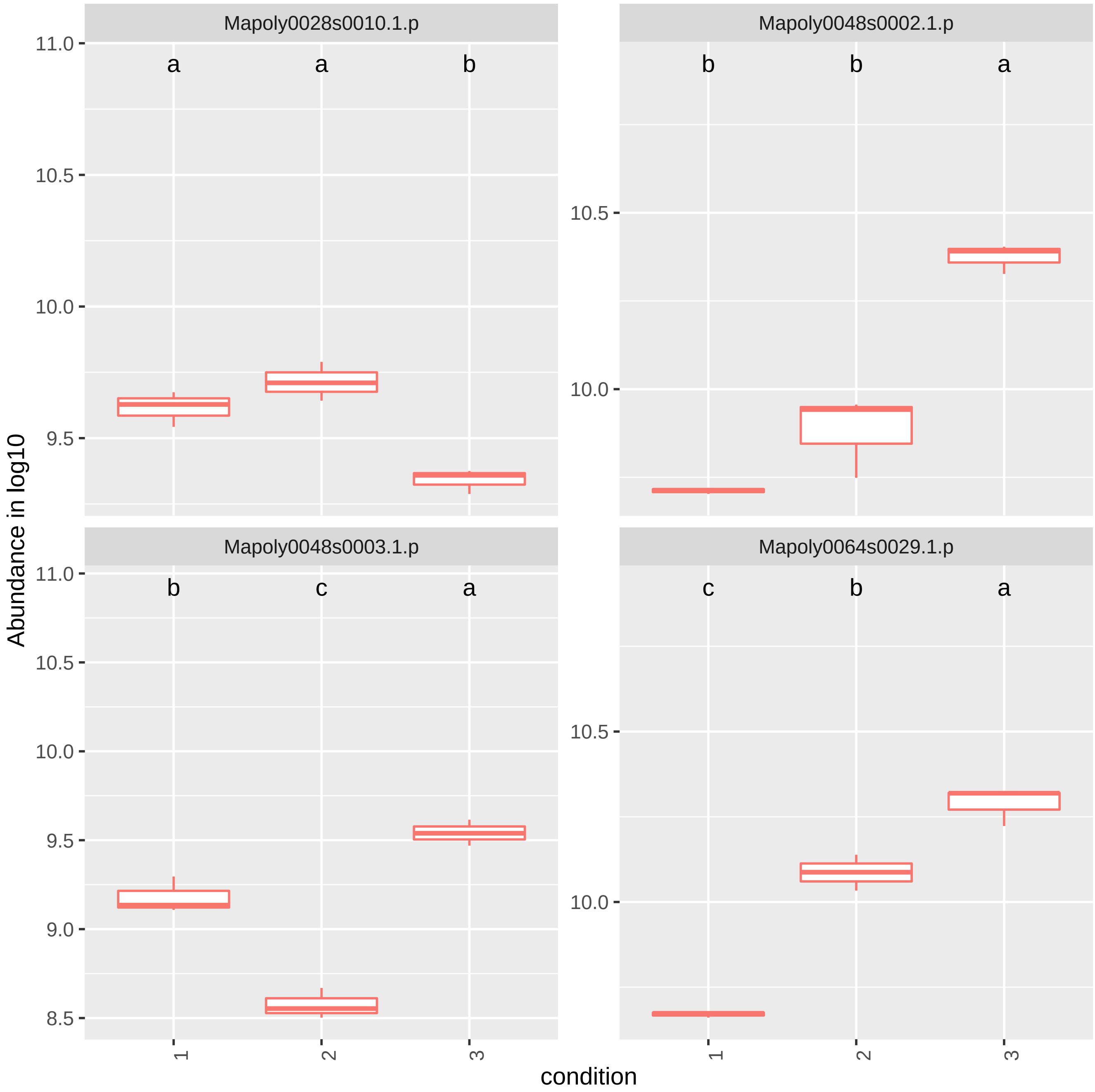

Mapoly0028s0010.1.p
Mapoly0048s0002.1.p
Mapoly0048s0003.1.p
Mapoly0064s0029.1.p
a
a
b
b
b
a
b
c
a
c
b
a
11.0
10.5
10.0
9.5
9.0
8.5
Abundance in log10
1
2
3
condition

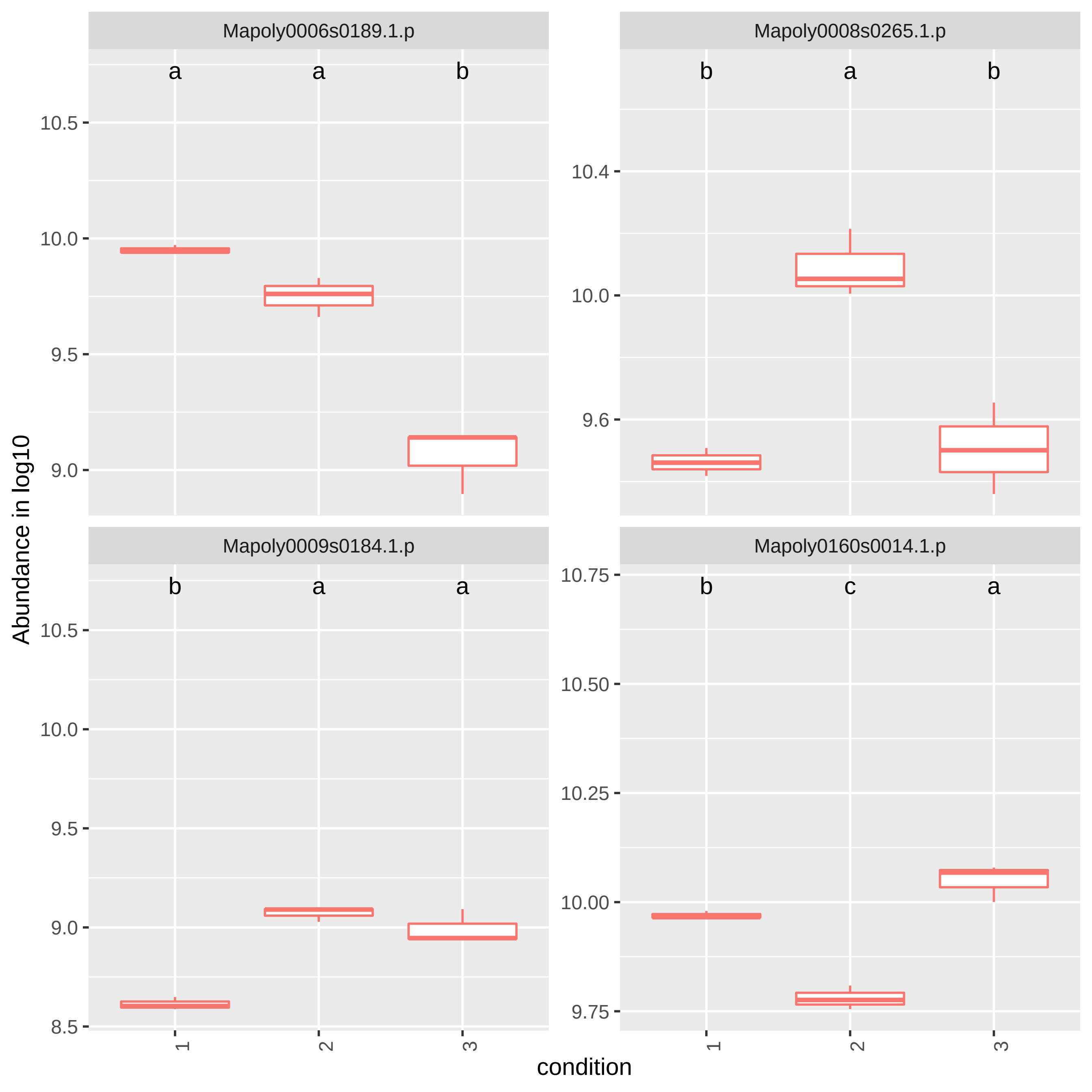

Mapoly0006s0189.1.p
Mapoly0008s0265.1.p
Mapoly0009s0184.1.p
Mapoly0160s0014.1.p
a
a
b
b
a
b
b
a
a
b
c
a
10.5
10.0
9.5
9.0
10.4
10.0
9.6
10.5
10.0
9.5
9.0
8.5
10.75
10.50
10.25
10.00
9.75
1
2
3
Abundance in log10
condition

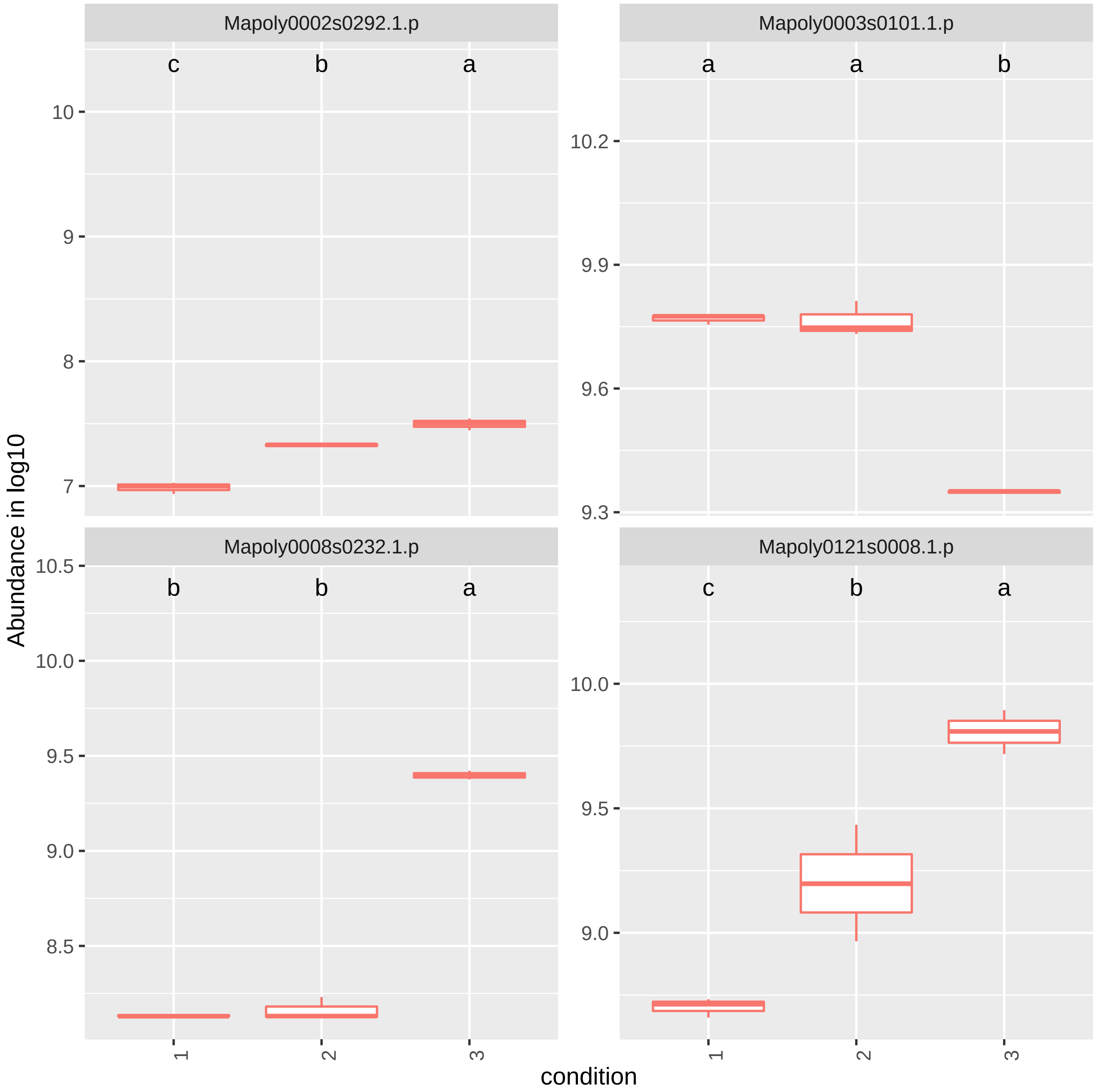

Mapoly0002s0292.1.p
c
b
a
10
9
8
7
Mapoly0003s0101.1.p
a
a
b
10.2
9.9
9.6
9.3
Mapoly0008s0232.1.p
b
b
a
10.5
10.0
9.5
9.0
8.5
Mapoly0121s0008.1.p
c
b
a
10.0
9.5
9.0
Abundance in log10
1
2
3
1
2
3
condition

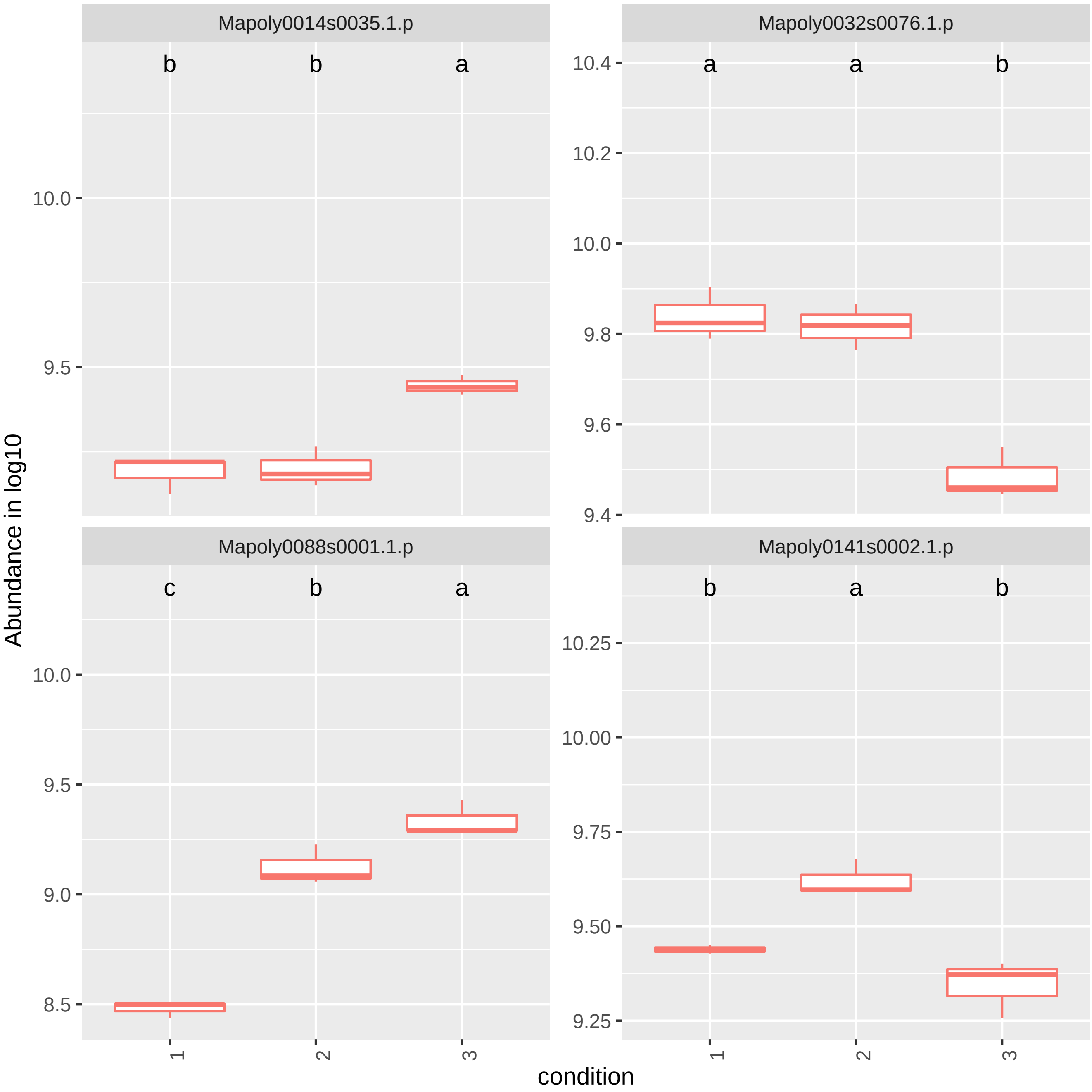

Mapoly0014s0035.1.p
Mapoly0032s0076.1.p
Mapoly0088s0001.1.p
Mapoly0141s0002.1.p
b b a
a a b
c b a
b a b
10.0
9.5
10.4
10.2
10.0
9.8
9.6
9.4
10.0
9.5
9.0
8.5
10.25
10.00
9.75
9.50
9.25
1
2
3
Abundance in log10
condition

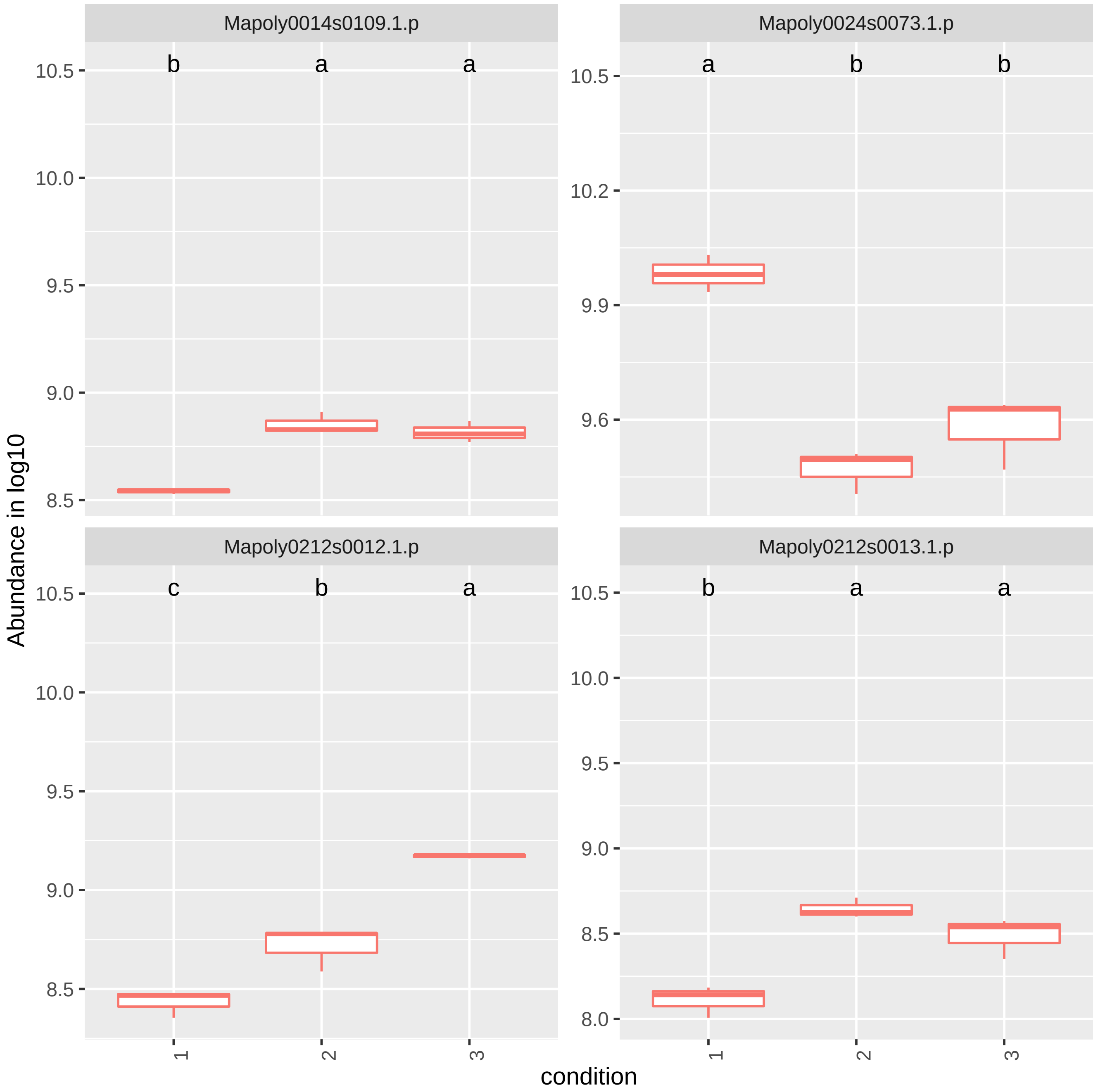

Mapoly0014s0109.1.p
Mapoly0024s0073.1.p
Mapoly0212s0012.1.p
Mapoly0212s0013.1.p
b a a
a b b
c b a
b a a
10.5
10.0
9.5
9.0
8.5
10.2
9.9
9.6
8.0
1 2 3
Abundance in log10
condition

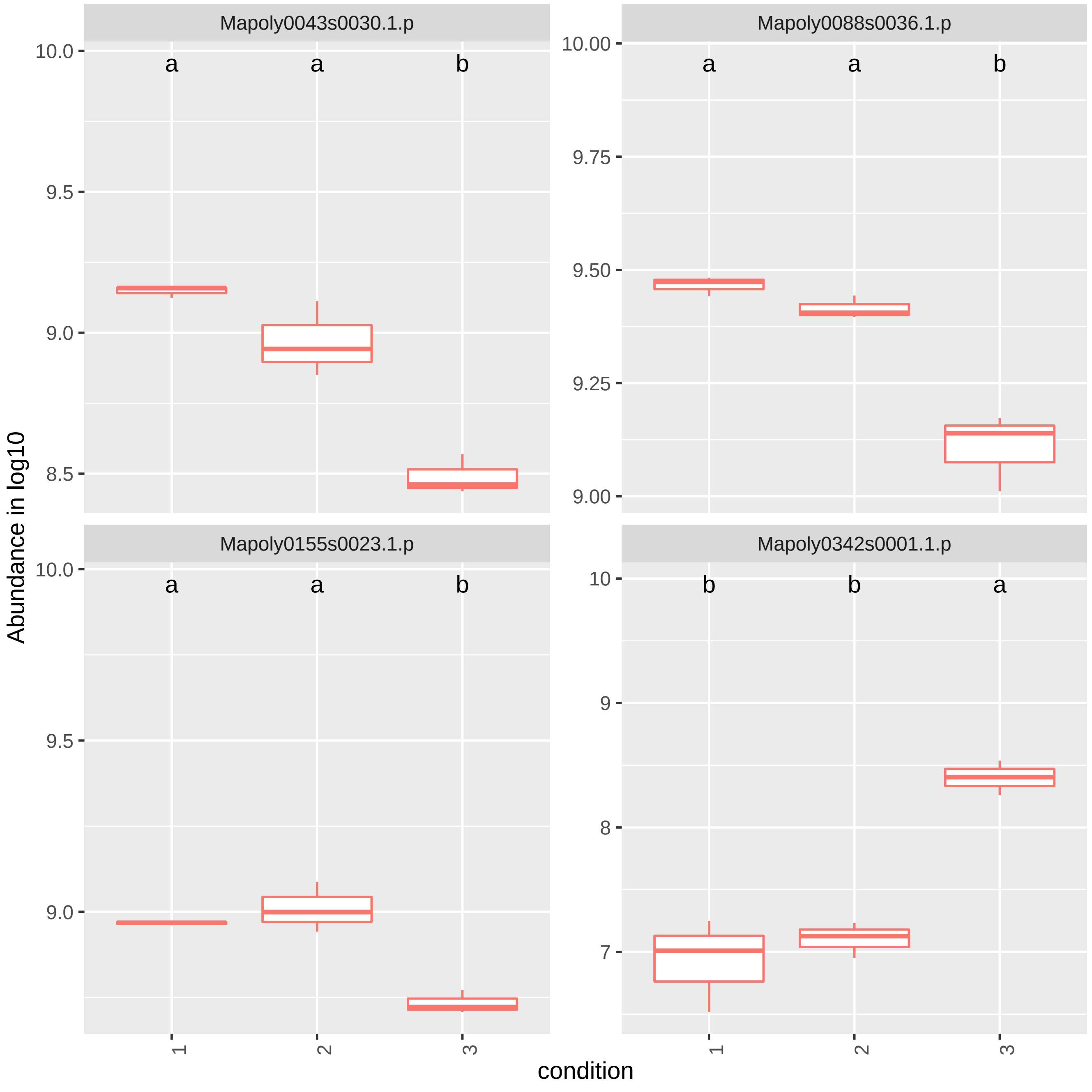
Mapoly0043s0030.1.p
Mapoly0088s0036.1.p
Mapoly0155s0023.1.p
Mapoly0342s0001.1.p
a
a
b
a
a
b
a
a
b
b
b
a
10.0
9.5
9.0
8.5
10.00
9.75
9.50
9.25
9.00
10.0
9.5
9.0
10
9
8
7
1
2
3
1
2
3
Abundance in log10
condition

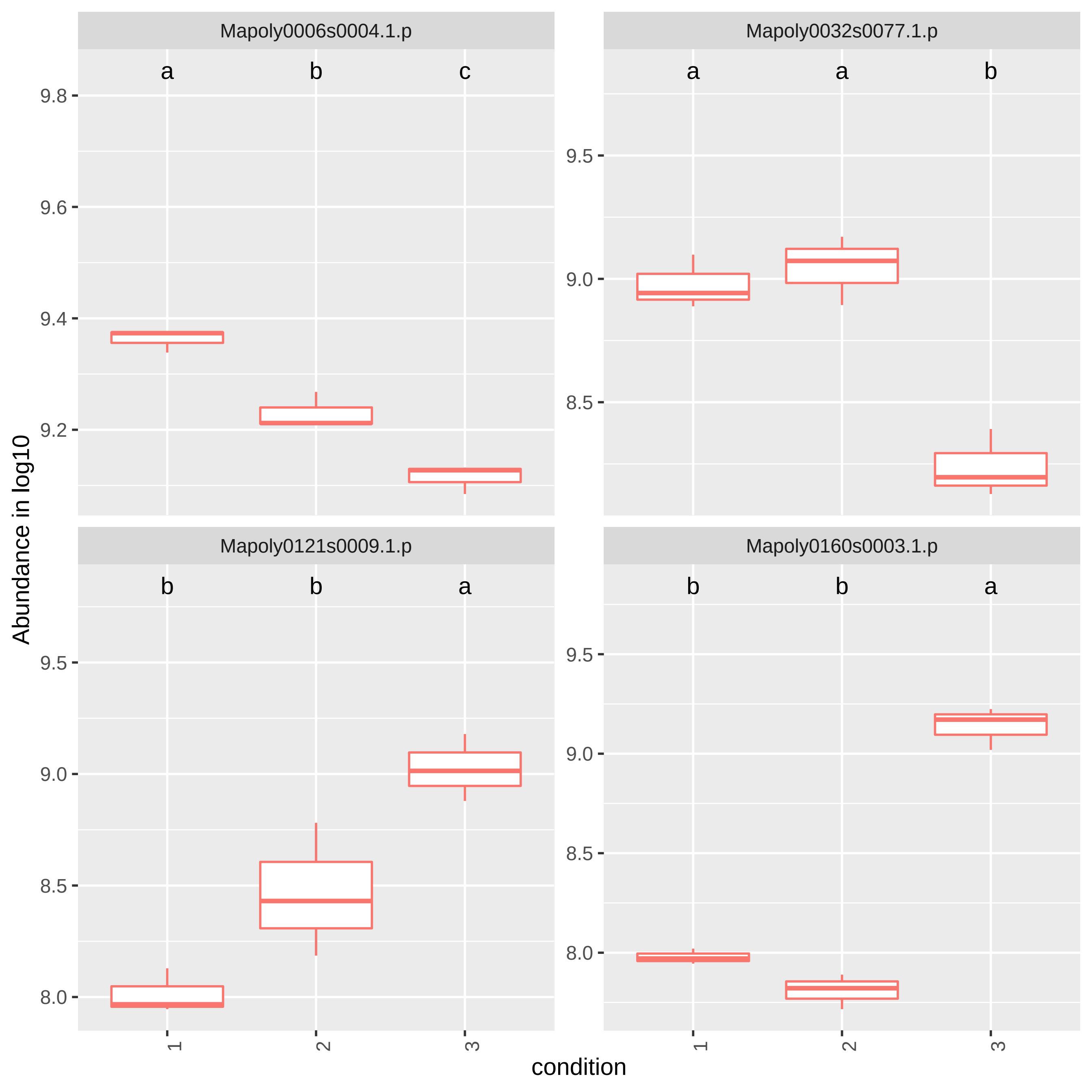

Mapoly0006s0004.1.p
Mapoly0032s0077.1.p
Mapoly0121s0009.1.p
Mapoly0160s0003.1.p
a
b
c
a
a
b
b
b
a
b
b
a
9.8
9.6
9.4
9.2
9.5
9.0
8.5
8.0
Abundance in log10
1
2
3
condition

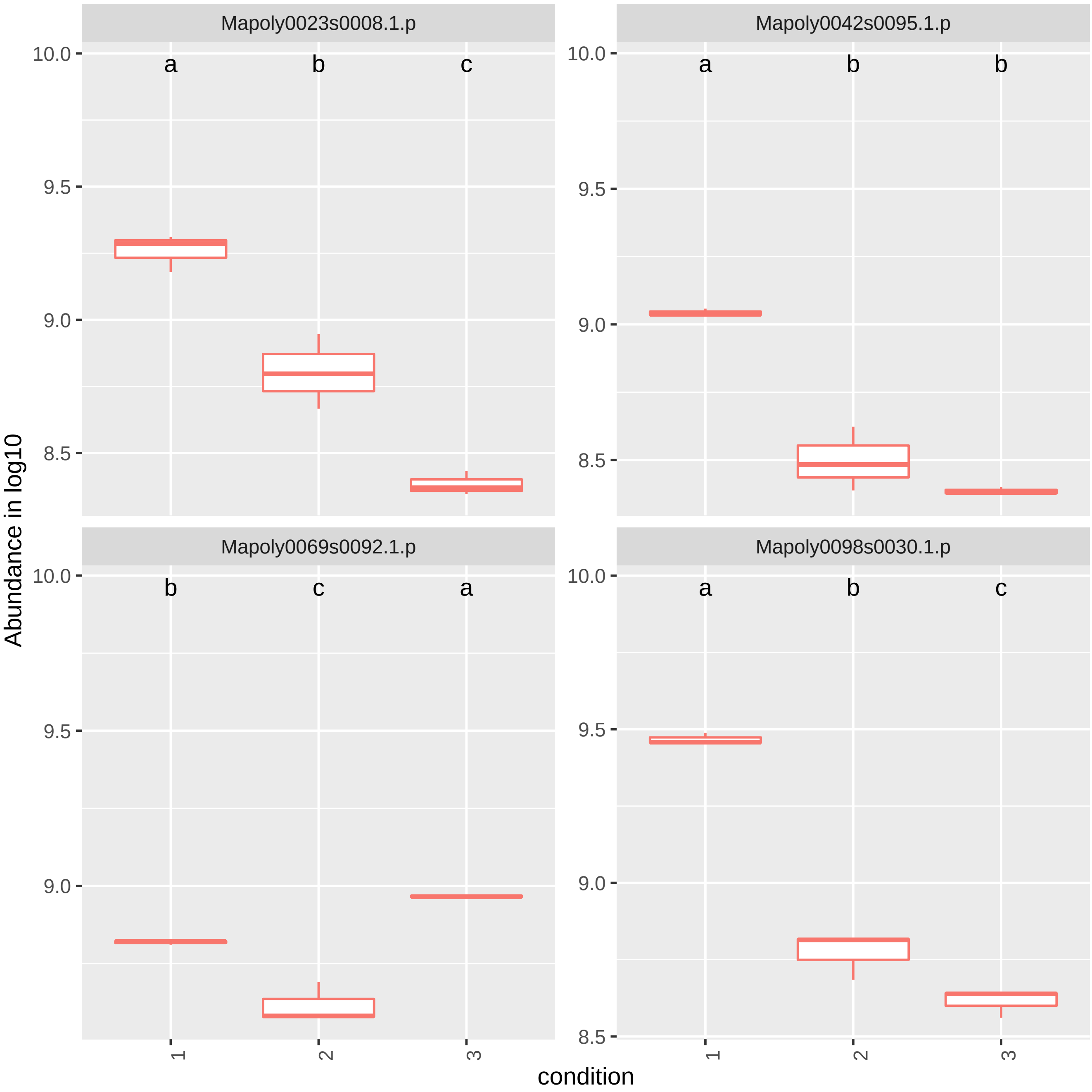
Mapoly0023s0008.1.p
Mapoly0042s0095.1.p
Mapoly0069s0092.1.p
Mapoly0098s0030.1.p
a b c
a b b
b c a
a b c
10.0
9.5
9.0
8.5
Abundance in log10
1 2 3
condition

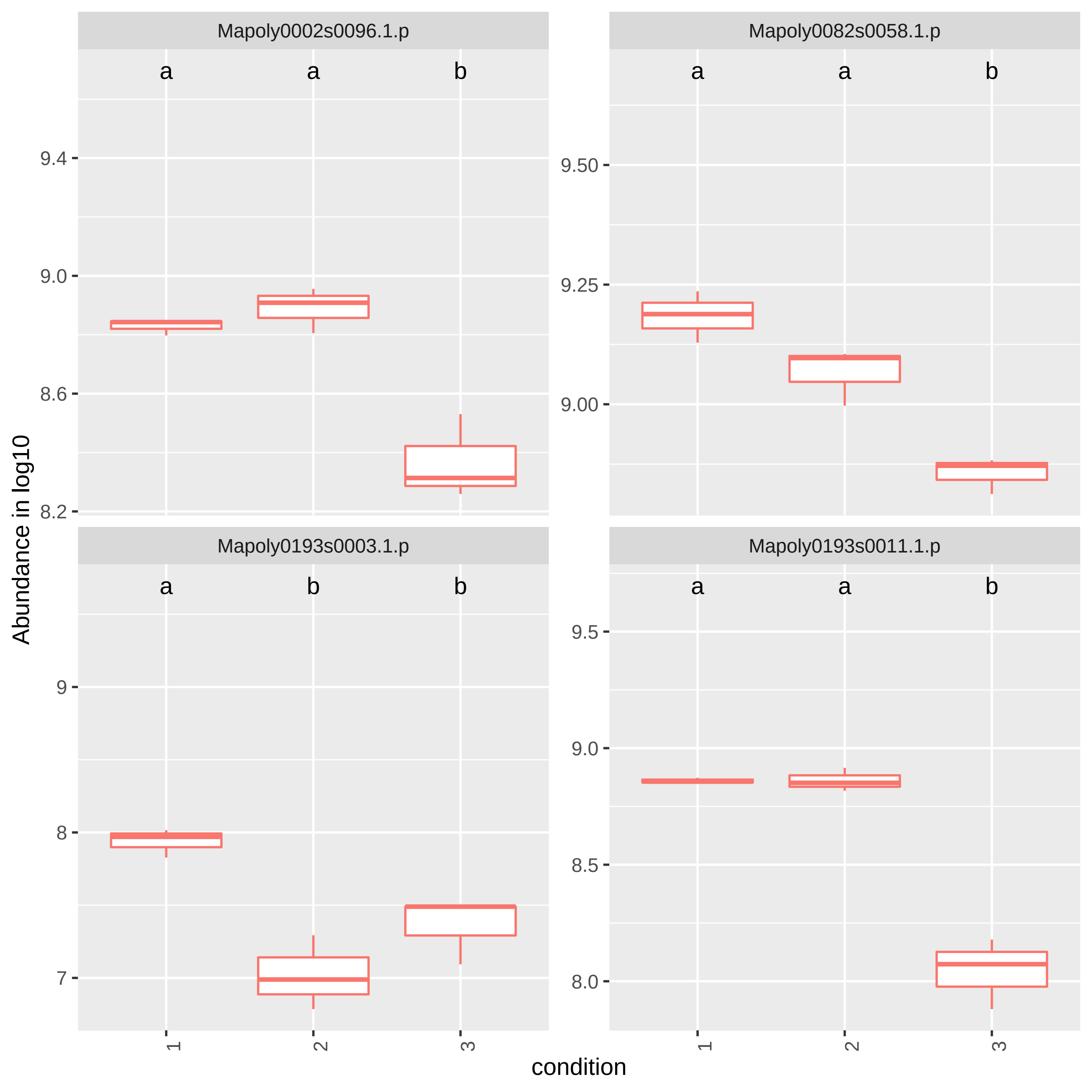

Mapoly0002s0096.1.p
Mapoly0082s0058.1.p
Mapoly0193s0003.1.p
Mapoly0193s0011.1.p
a
a
b
a
a
b
a
b
b
a
a
b
9.4
9.0
8.6
8.2
9.50
9.25
9.00
9
8
7
9.5
9.0
8.5
8.0
1
2
3
1
2
3
Abundance in log10
condition

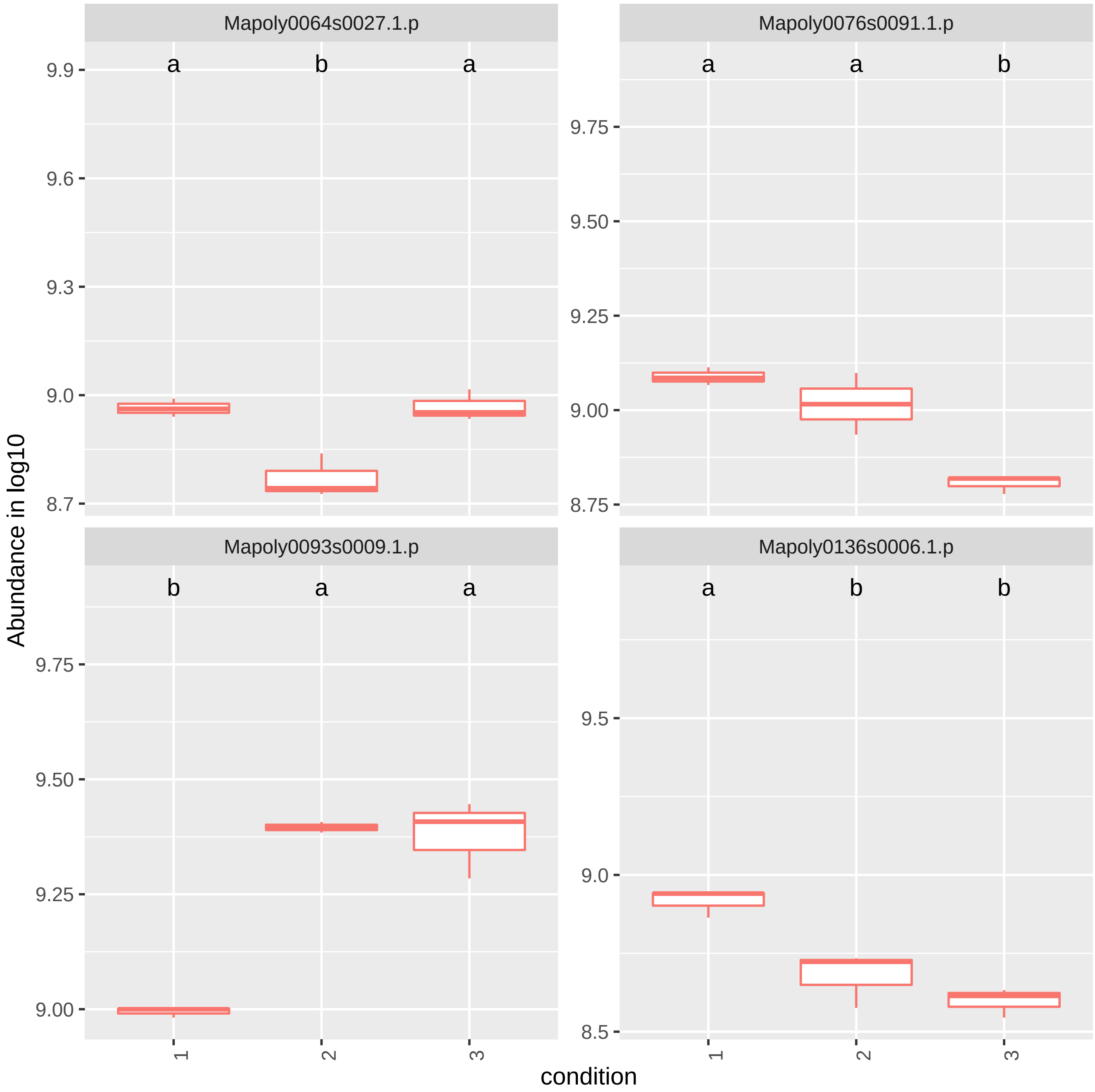

Mapoly0064s0027.1.p
Mapoly0076s0091.1.p
Mapoly0093s0009.1.p
Mapoly0136s0006.1.p
a b a
a a b
b a a
a b b
9.9
9.6
9.3
9.0
8.7
9.75
9.50
9.25
9.00
8.75
9.5
8.5
1 2 3
Abundance in log10
condition

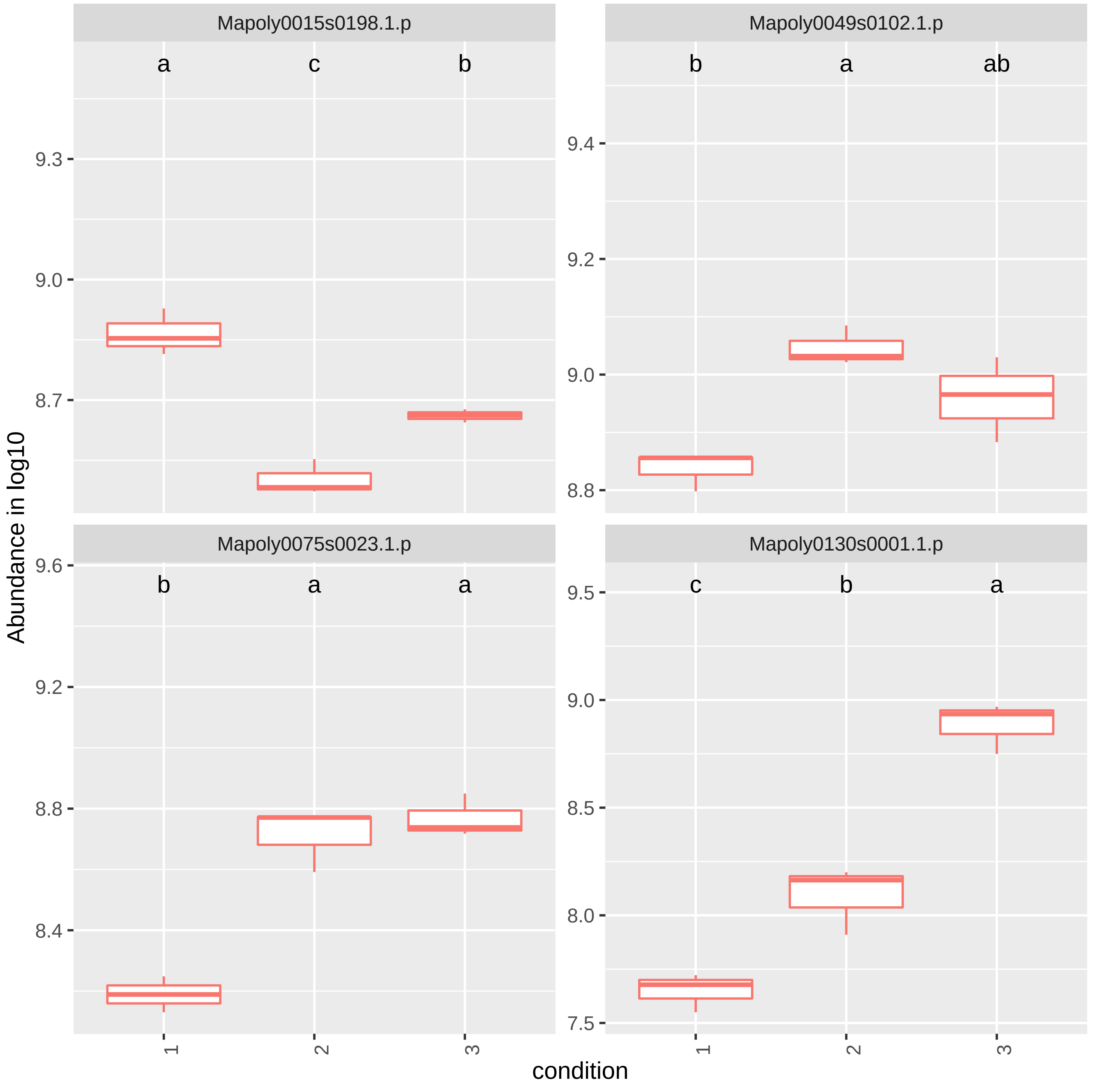
Mapoly0015s0198.1.p
Mapoly0049s0102.1.p
Mapoly0075s0023.1.p
Mapoly0130s0001.1.p
a
c
b
b
a
ab
b
a
a
c
b
a
9.3
9.0
8.7
9.4
9.2
9.0
8.8
9.6
9.2
8.8
8.4
9.5
9.0
8.5
8.0
7.5
1
2
3
Abundance in log10
condition

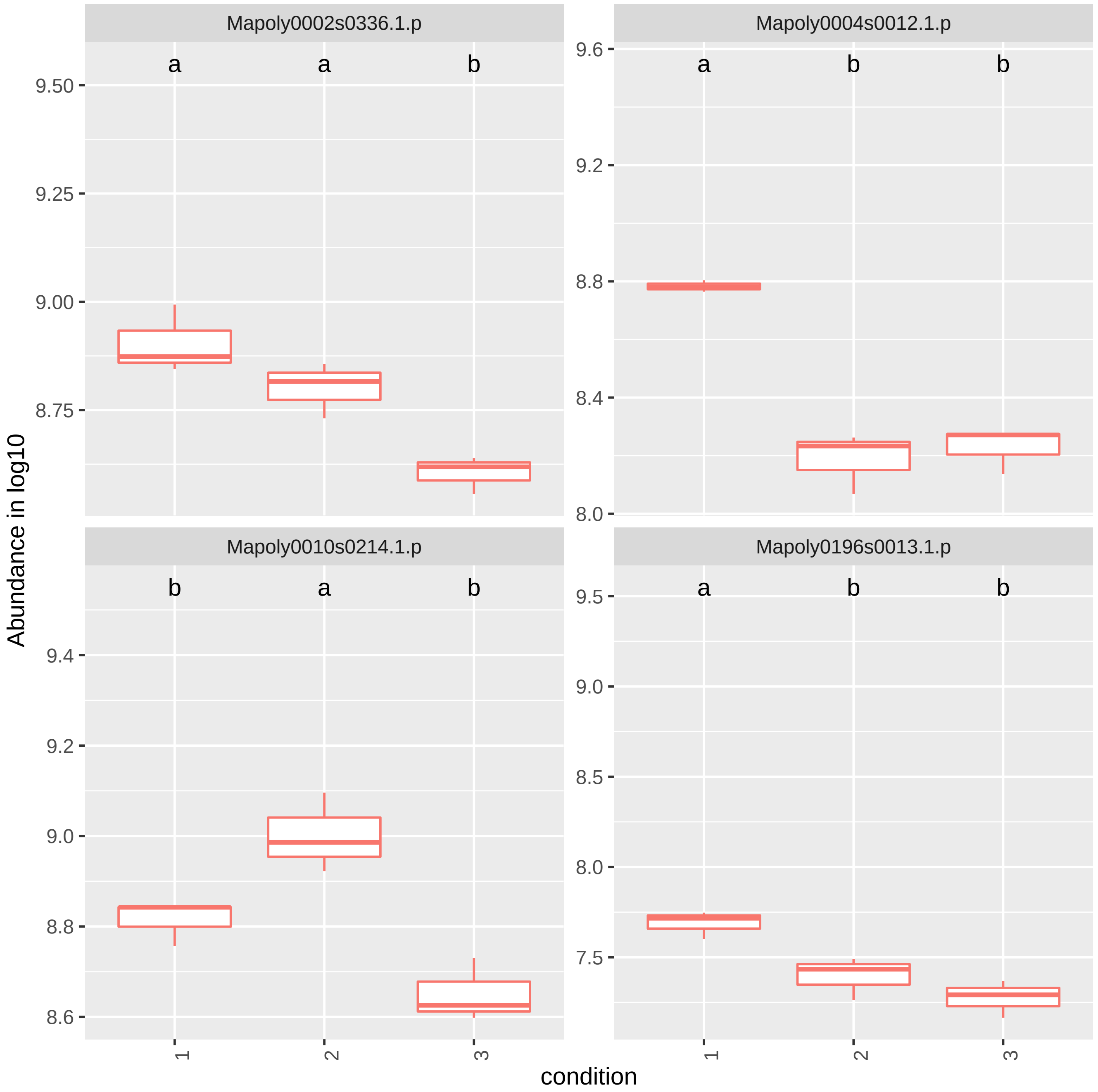

Mapoly0002s0336.1.p
Mapoly0004s0012.1.p
Mapoly0010s0214.1.p
Mapoly0196s0013.1.p
a a b
a b b
b a b
a b b
9.50
9.25
9.00
8.75
9.6
9.2
8.8
8.4
8.0
9.4
9.2
9.0
8.8
8.6
9.5
9.0
8.5
8.0
7.5
Abundance in log10
1 2 3
condition

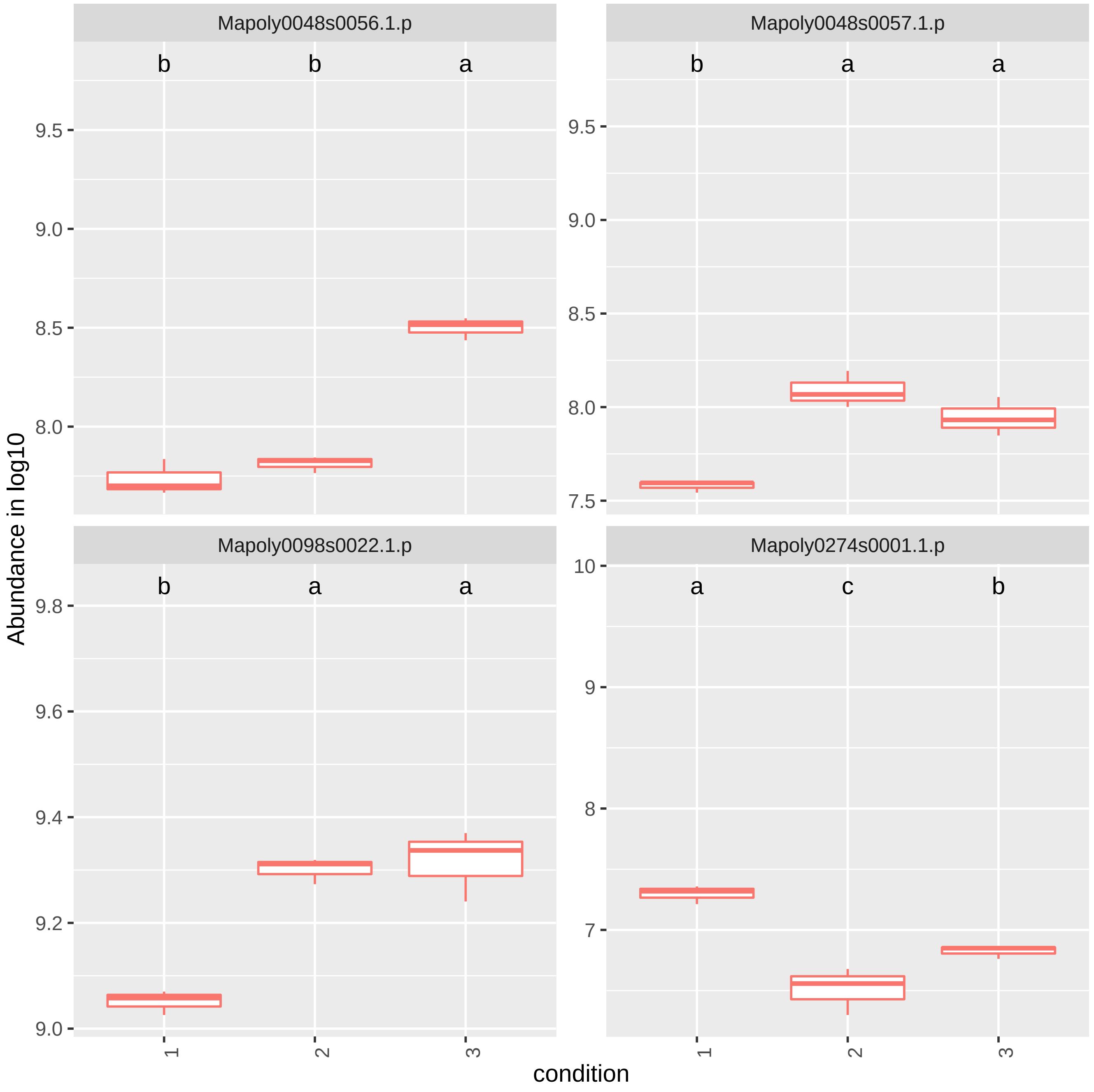

Mapoly0048s0056.1.p
b
b
a
9.5
9.0
8.5
8.0
Mapoly0048s0057.1.p
b
a
a
9.5
9.0
8.5
8.0
7.5
Mapoly0098s0022.1.p
b
a
a
9.8
9.6
9.4
9.2
9.0
Mapoly0274s0001.1.p
a
c
b
10
9
8
7
Abundance in log10
1
2
3
1
2
3
condition

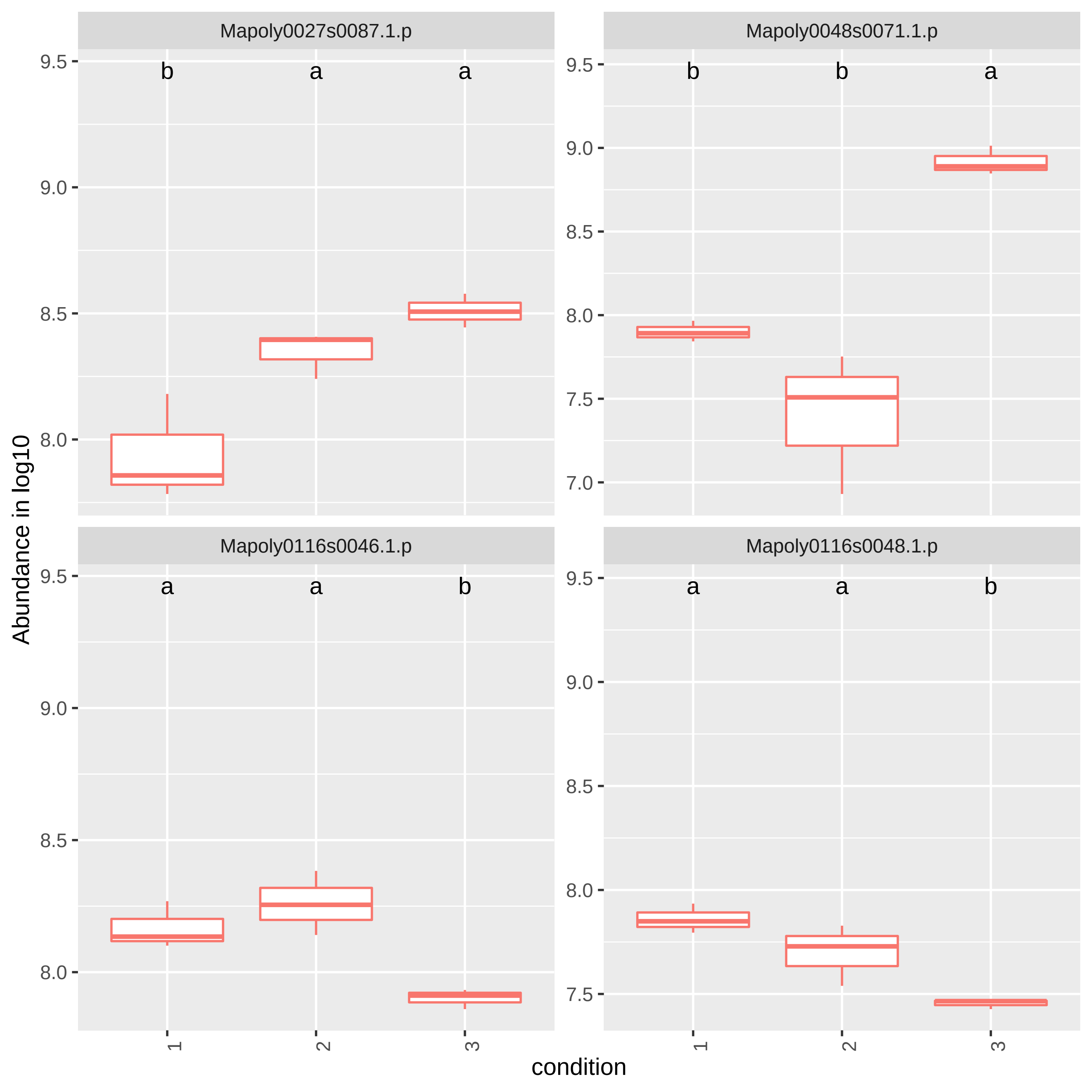
Mapoly0027s0087.1.p
Mapoly0048s0071.1.p
Mapoly0116s0046.1.p
Mapoly0116s0048.1.p
b a a
b b a
a a b
a a b
Abundance in log10
condition
1 2 3

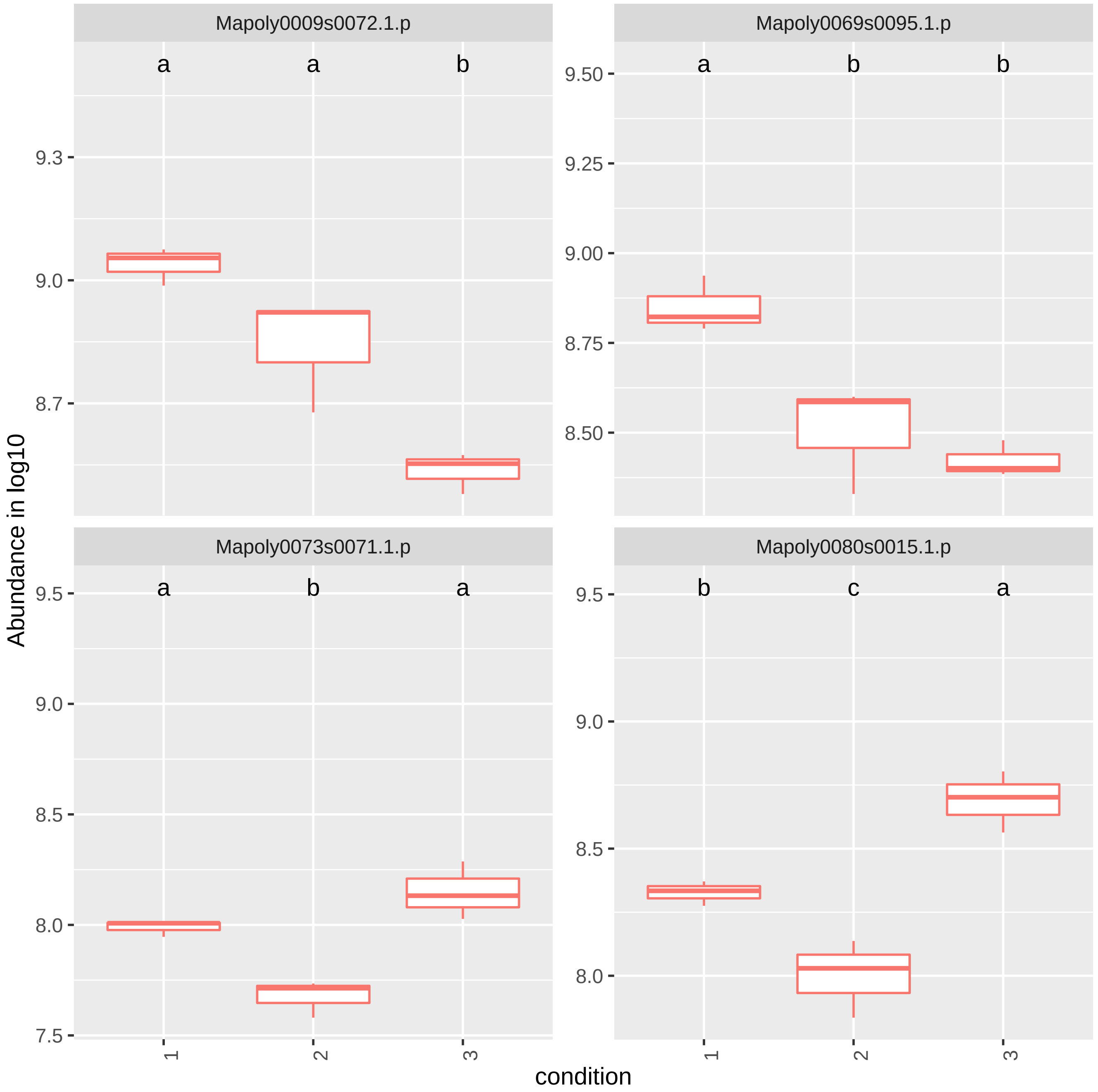

Mapoly0009s0072.1.p
Mapoly0069s0095.1.p
Mapoly0073s0071.1.p
Mapoly0080s0015.1.p
a
a
b
a
b
b
a
b
a
b
c
a
9.3
9.0
8.7
9.50
9.25
9.00
8.75
8.50
9.5
9.0
8.5
8.0
7.5
9.5
9.0
8.5
8.0
Abundance in log10
1
2
3
1
2
3
condition

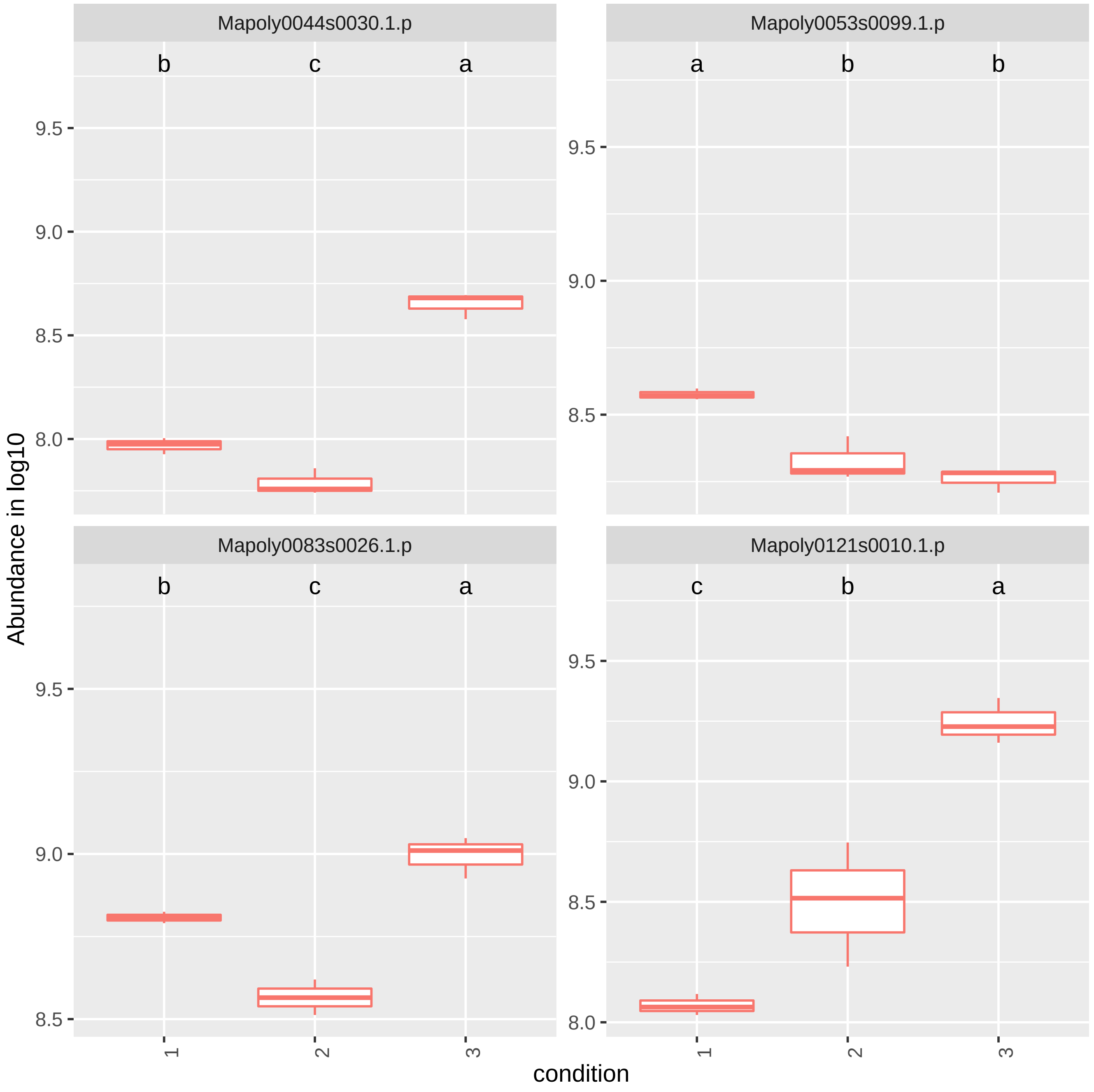

Mapoly0044s0030.1.p
b
c
a
9.5
9.0
8.5
8.0
Mapoly0053s0099.1.p
a
b
b
9.5
9.0
8.5
Mapoly0083s0026.1.p
b
c
a
9.5
9.0
8.5
Mapoly0121s0010.1.p
c
b
a
9.5
9.0
8.5
8.0
Abundance in log10
1
2
3
1
2
3
condition

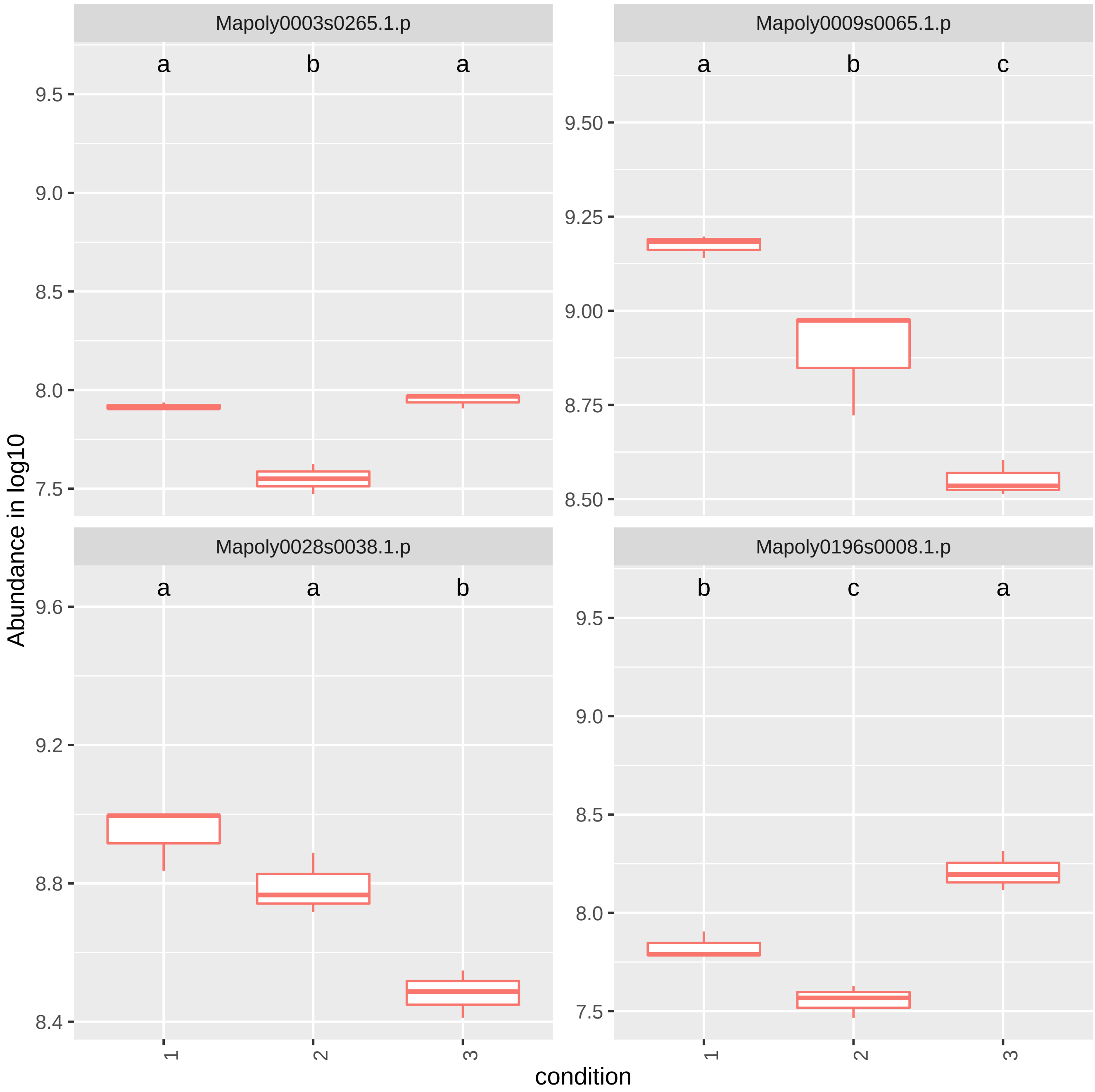
Mapoly0003s0265.1.p
Mapoly0009s0065.1.p
Mapoly0028s0038.1.p
Mapoly0196s0008.1.p
a
b
a
a
b
c
a
a
b
b
c
a
9.5
9.0
8.5
8.0
7.5
9.50
9.25
9.00
8.75
8.50
9.6
9.2
8.8
8.4
9.5
9.0
8.5
8.0
7.5
1
2
3
1
2
3
Abundance in log10
condition

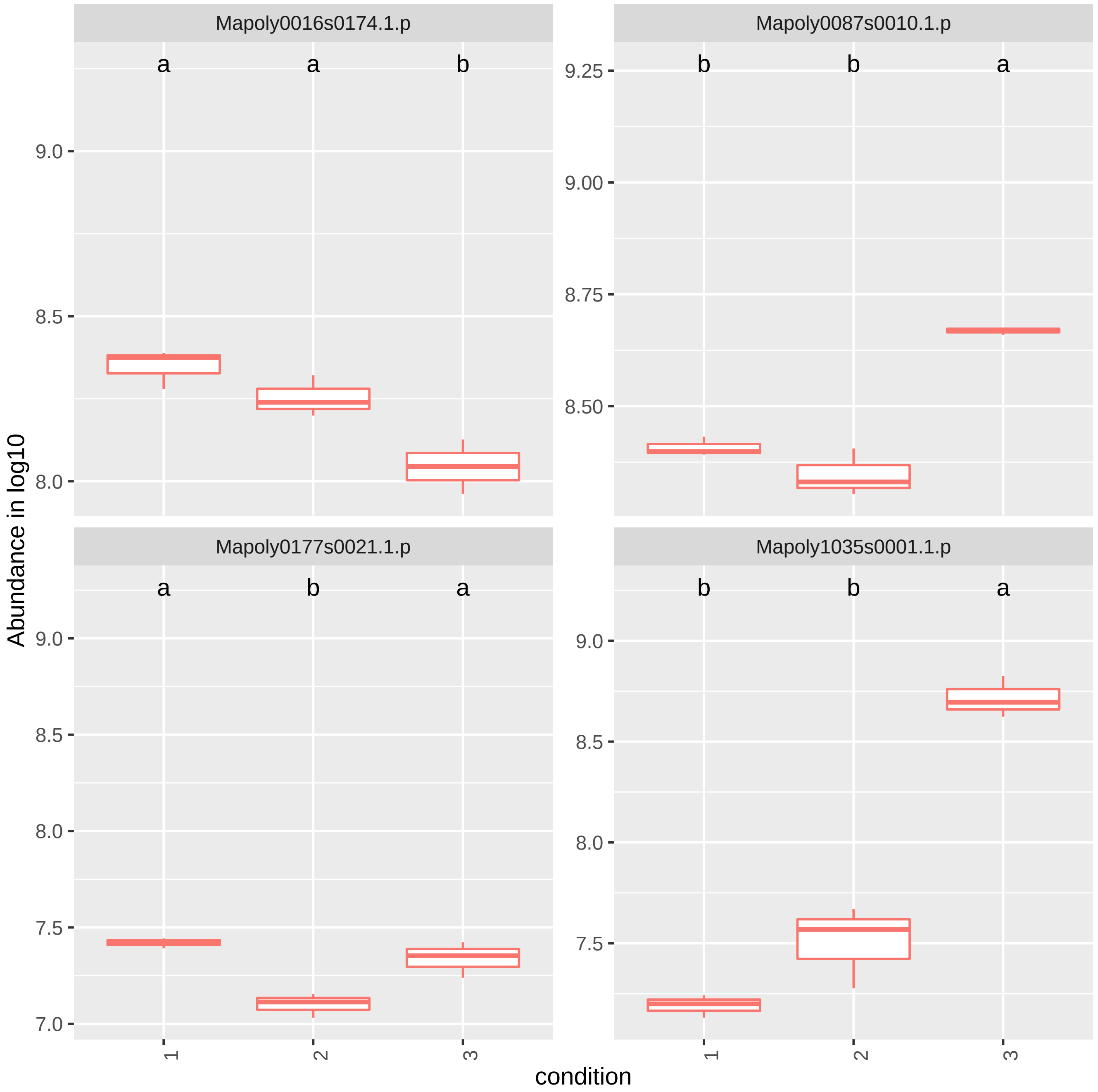

Mapoly0016s0174.1.p
Mapoly0087s0010.1.p
Mapoly0177s0021.1.p
Mapoly1035s0001.1.p
Abundance in log10
condition

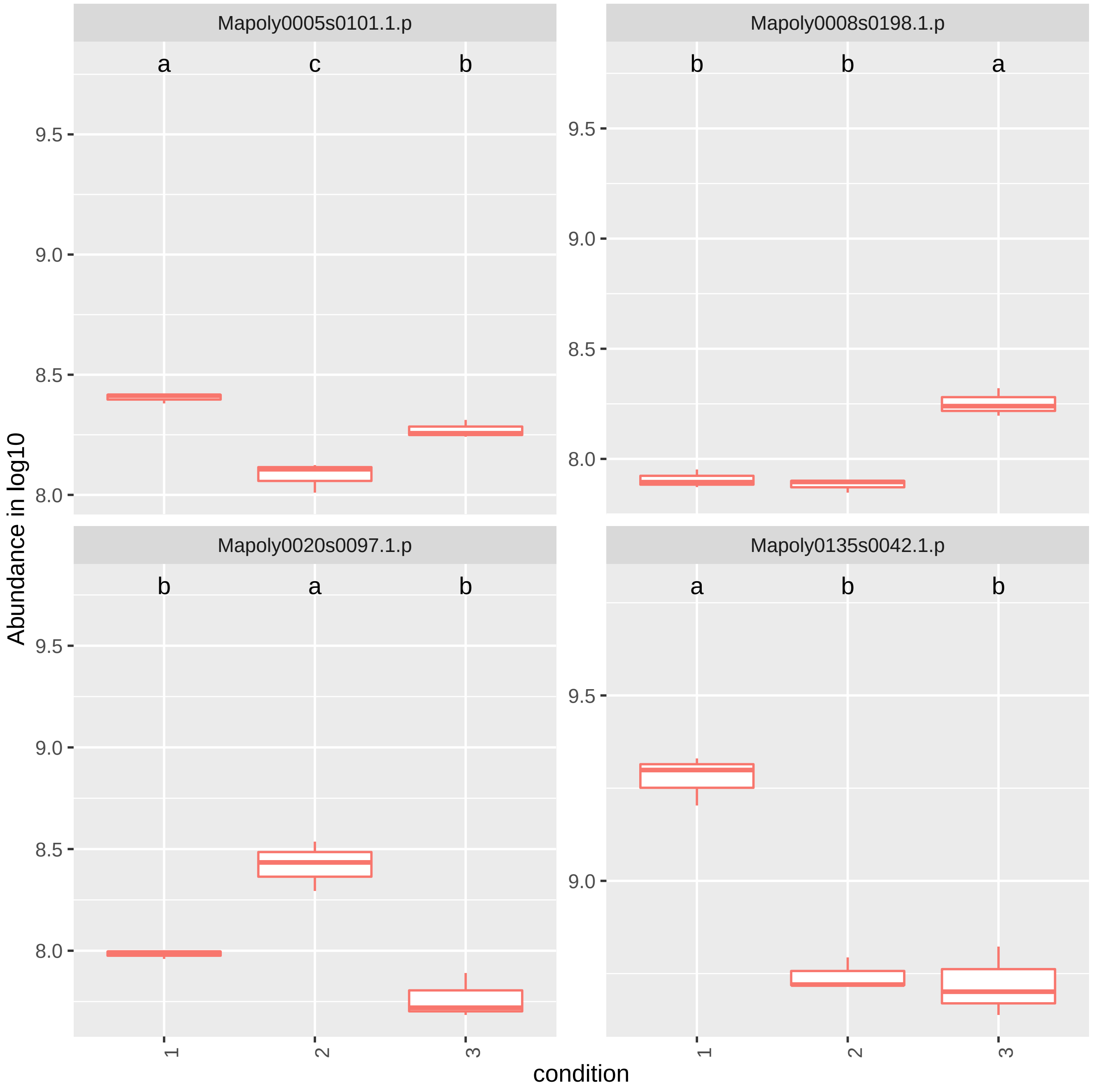
Mapoly0005s0101.1.p
Mapoly0008s0198.1.p
Mapoly0020s0097.1.p
Mapoly0135s0042.1.p
a c b
b b a
b a b
a b b
Abundance in log10
condition
9.5
9.0
8.5
8.0
1
2
3

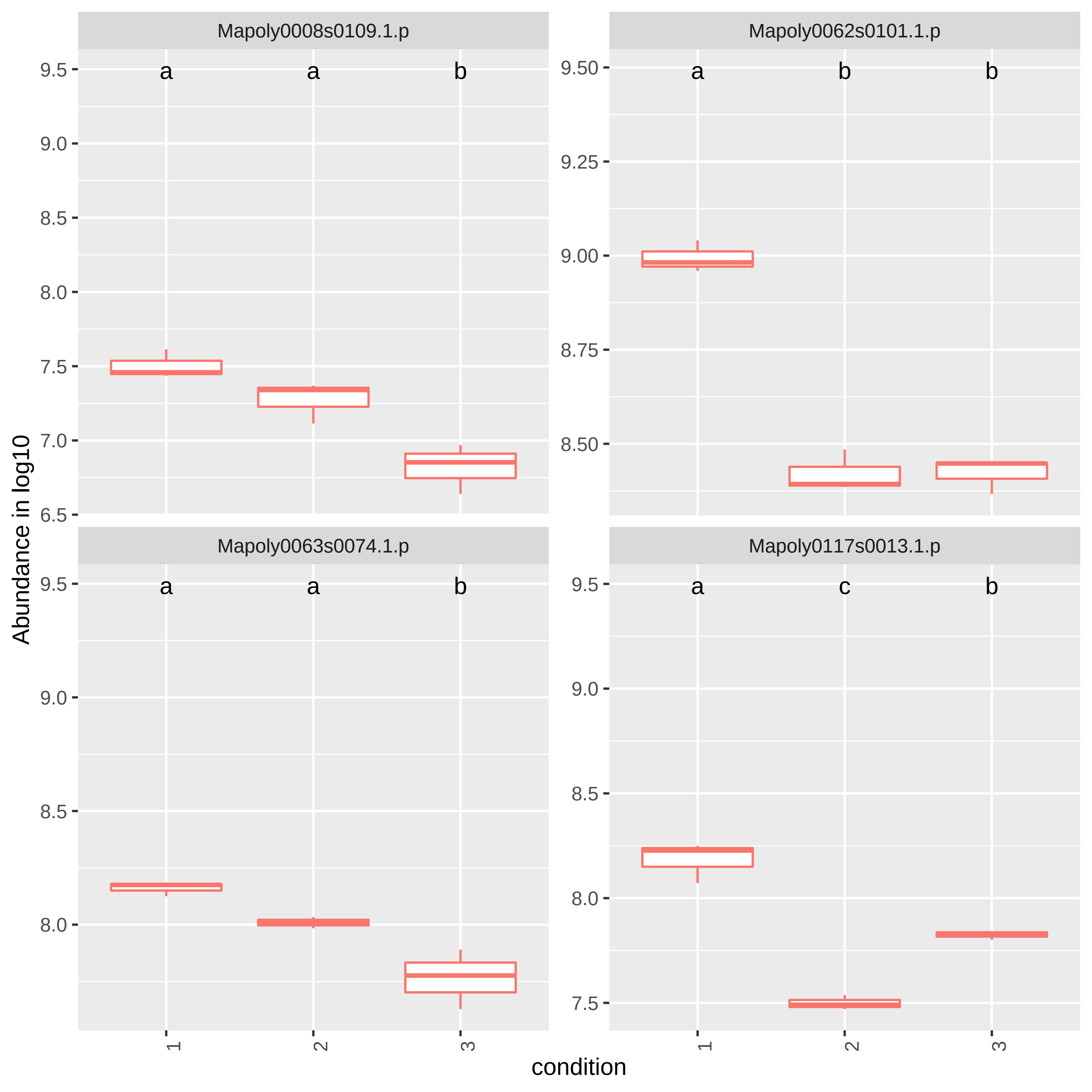

Mapoly0008s0109.1.p
Mapoly0062s0101.1.p
Mapoly0063s0074.1.p
Mapoly0117s0013.1.p
a a b
a b b
a a b
a c b
Abundance in log10
condition
1 2 3
9.5
9.0
8.5
8.0
7.5
7.0
6.5
9.50
9.25
9.00
8.75
8.50

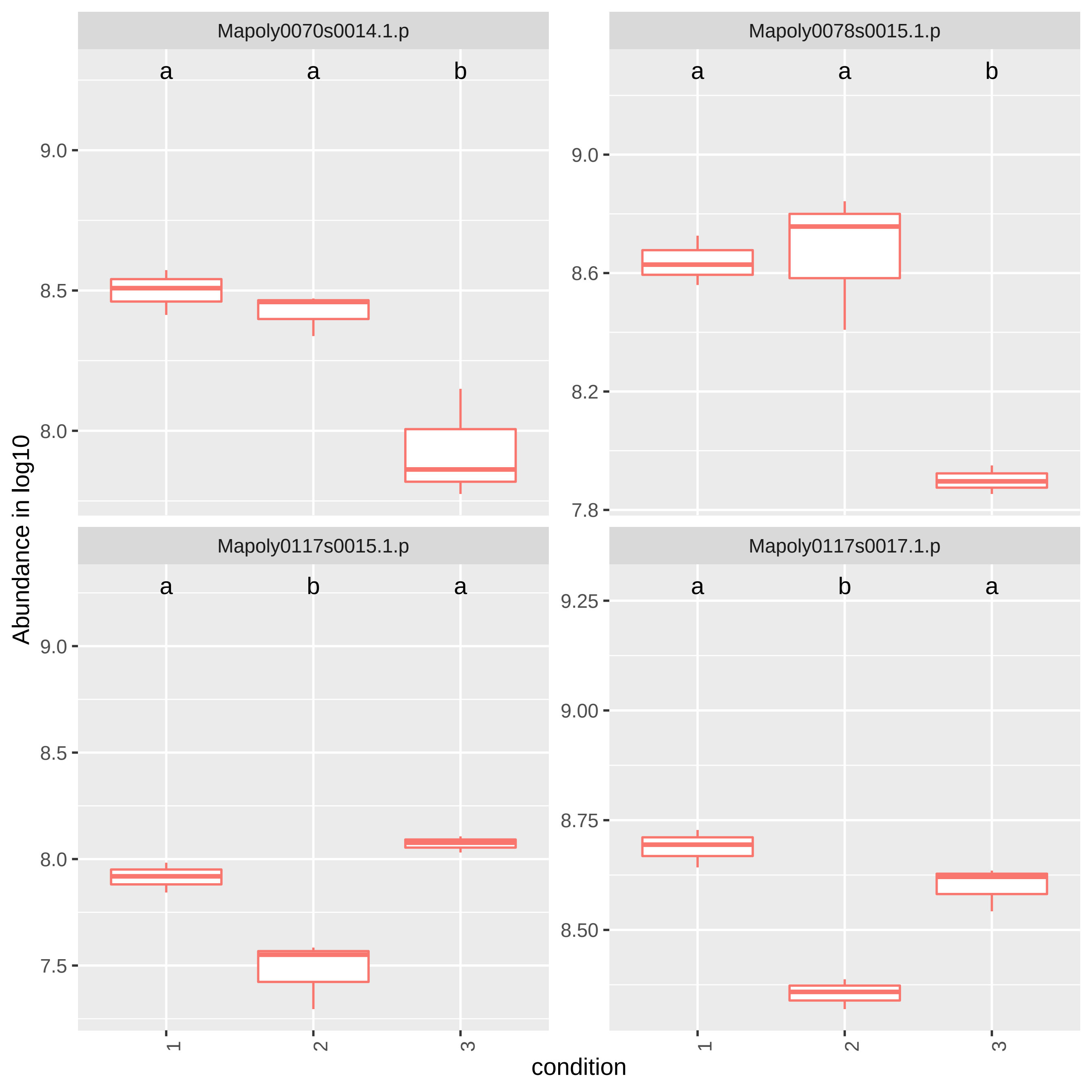

Mapoly0070s0014.1.p
Mapoly0078s0015.1.p
Mapoly0117s0015.1.p
Mapoly0117s0017.1.p
a
a
b
a
a
b
a
b
a
a
b
a
9.0
8.5
8.0
9.0
8.6
8.2
7.8
9.0
8.5
8.0
7.5
9.25
9.00
8.75
8.50
1
2
3
1
2
3
Abundance in log10
condition

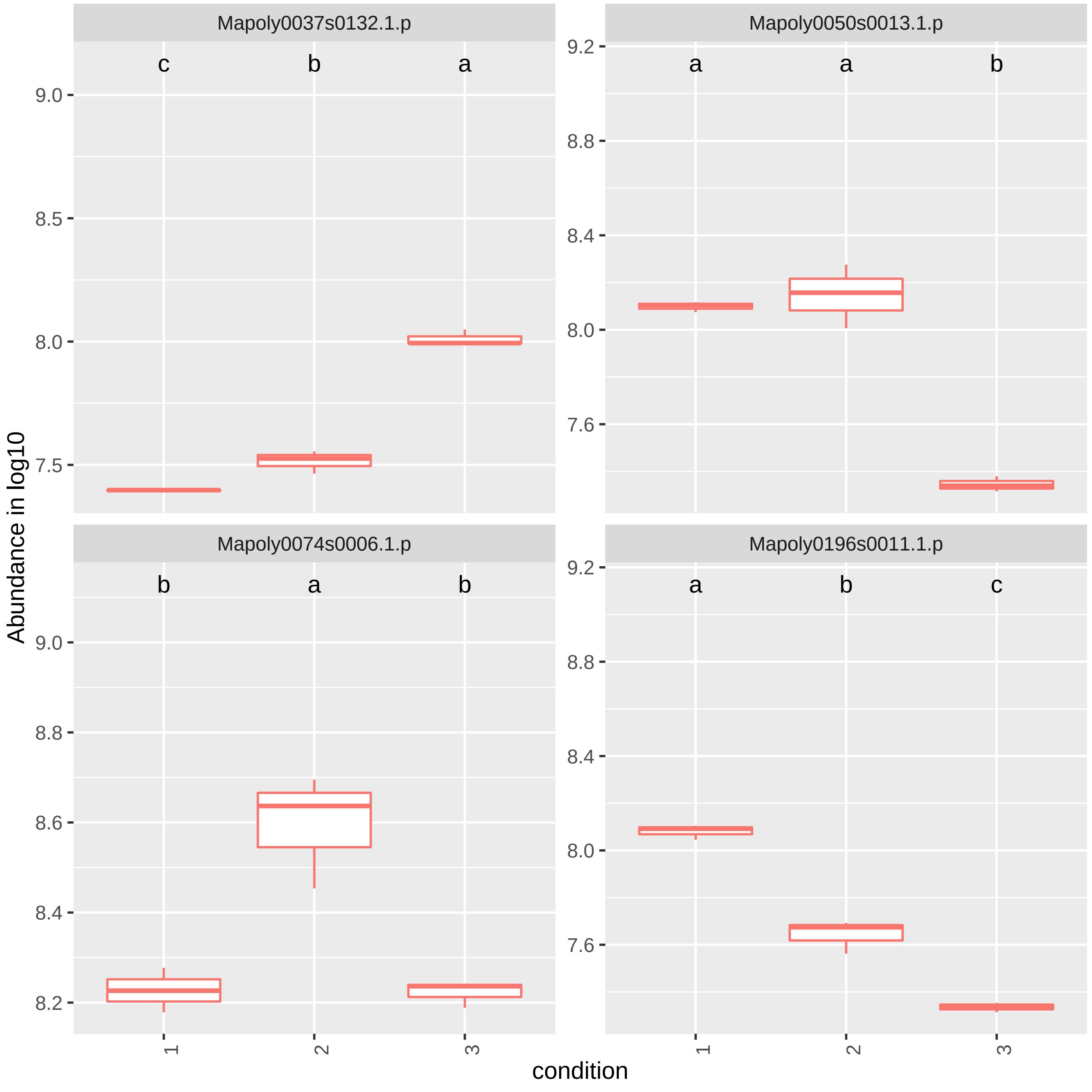

Mapoly0037s0132.1.p
Mapoly0050s0013.1.p
Mapoly0074s0006.1.p
Mapoly0196s0011.1.p
c
b
a
a
a
b
b
a
b
a
b
c
9.0
8.5
8.0
7.5
9.2
8.8
8.4
8.0
7.6
9.0
8.8
8.6
8.4
8.2
9.2
8.8
8.4
8.0
7.6
1
2
3
Abundance in log10
condition

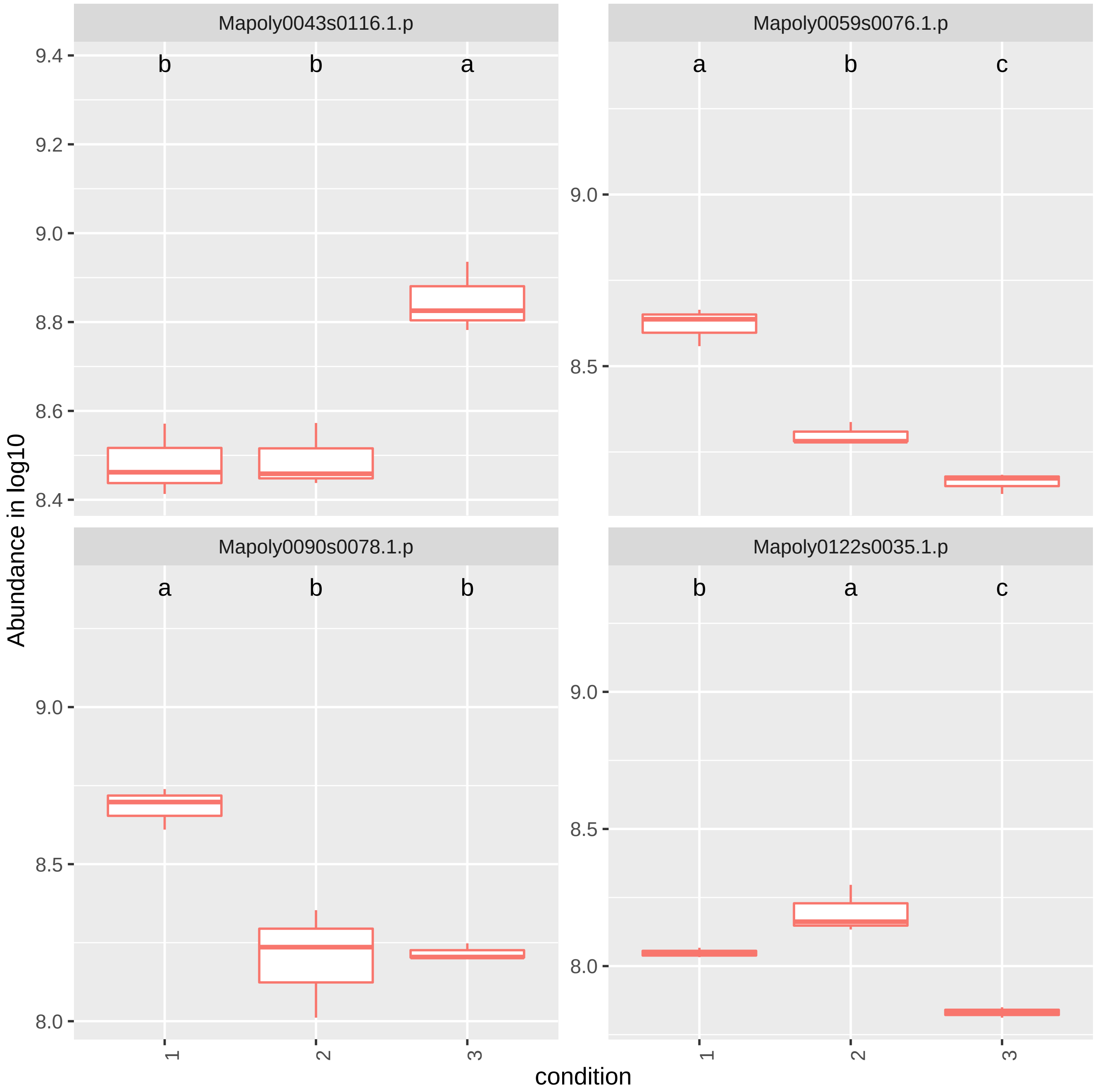

Mapoly0043s0116.1.p
Mapoly0059s0076.1.p
Mapoly0090s0078.1.p
Mapoly0122s0035.1.p
b
b
a
a
b
c
a
b
b
b
a
c
9.4
9.2
9.0
8.8
8.6
8.4
9.0
8.5
9.0
8.5
8.0
9.0
8.5
8.0
1
2
3
1
2
3
Abundance in log10
condition

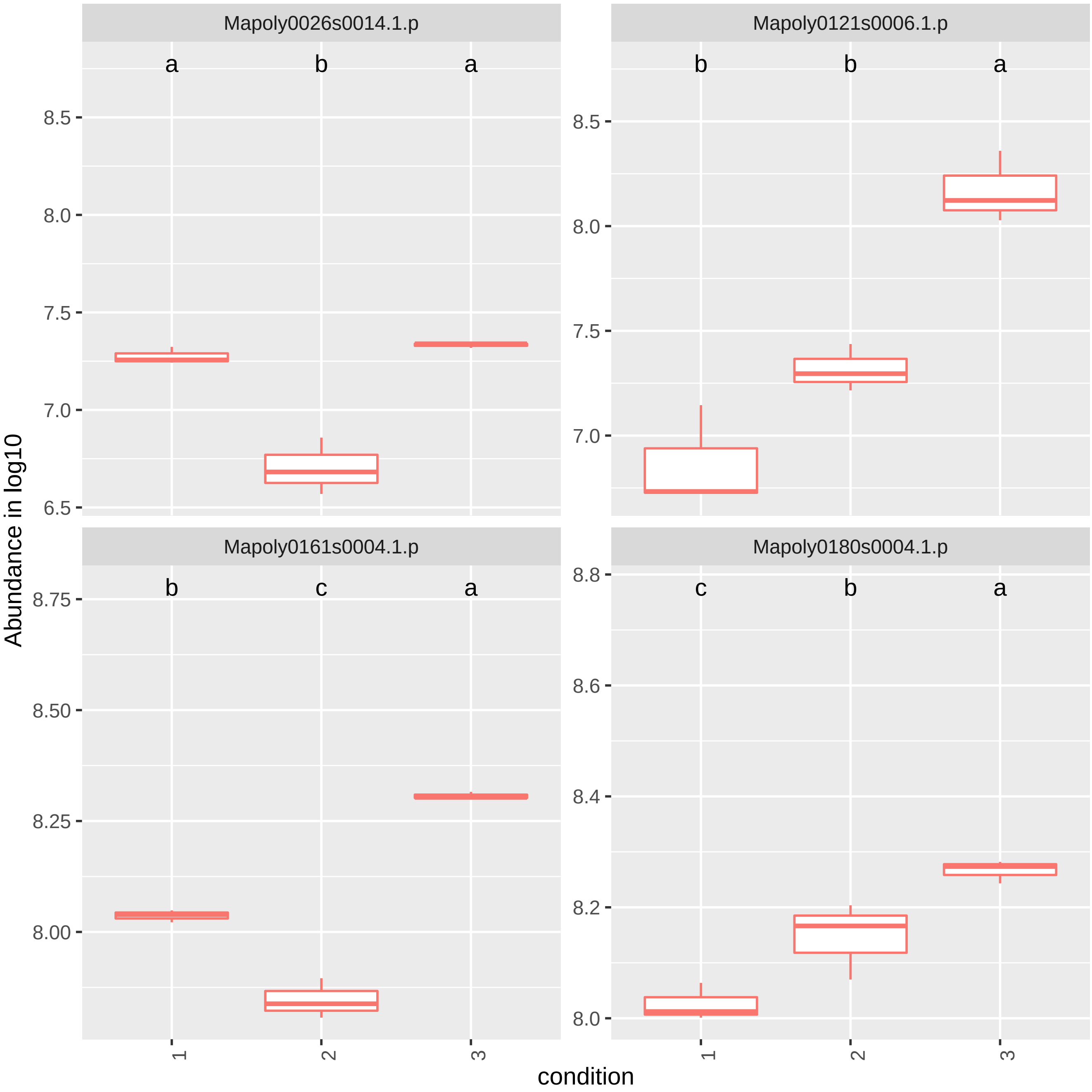
Mapoly0026s0014.1.p
Mapoly0121s0006.1.p
Mapoly0161s0004.1.p
Mapoly0180s0004.1.p
a
b
a
b
b
a
b
c
a
c
b
a
8.5
8.0
7.5
7.0
6.5
8.75
8.50
8.25
8.00
8.8
8.6
8.4
8.2
Abundance in log10
1
2
3
condition

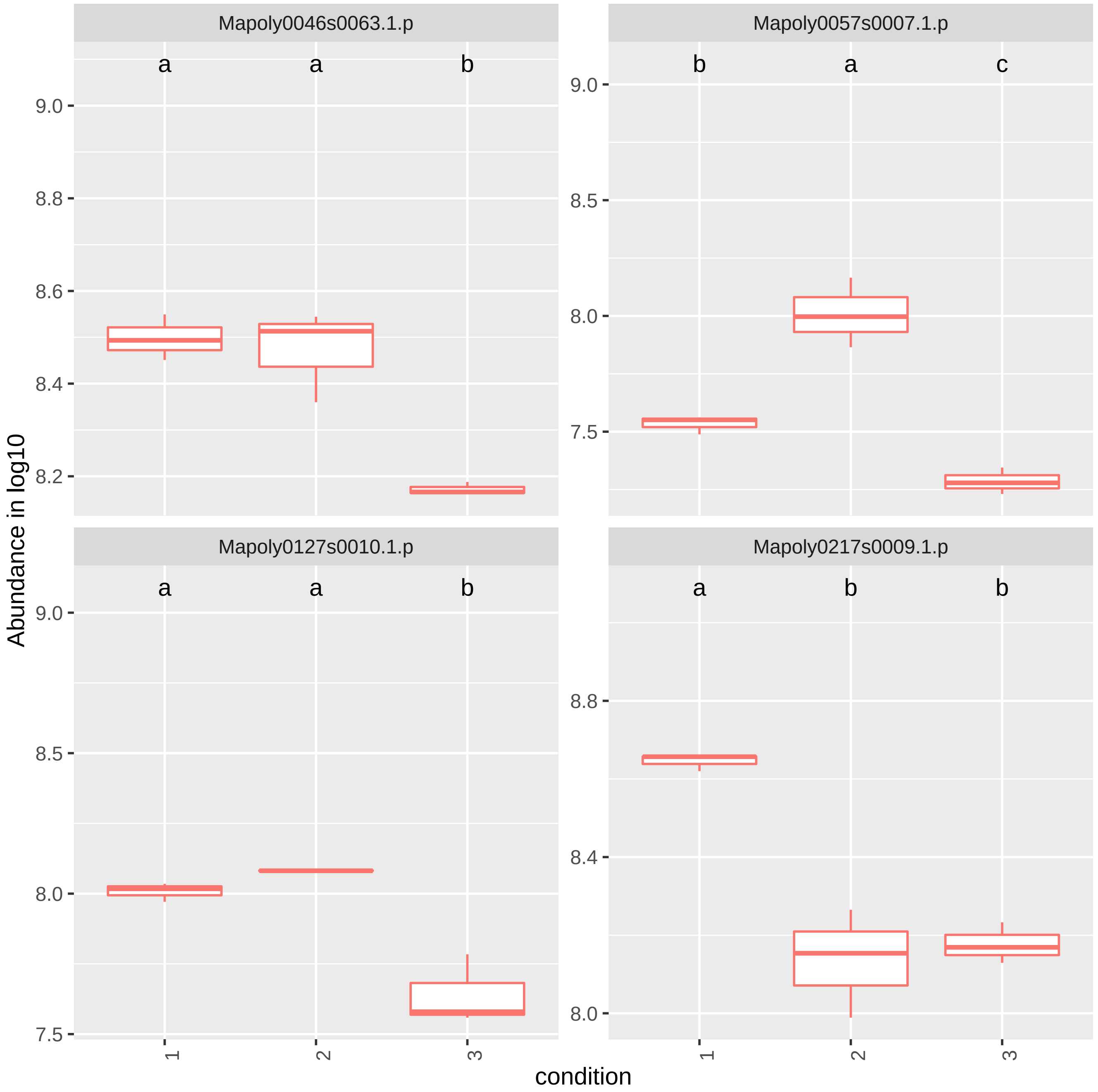

Mapoly0046s0063.1.p
Mapoly0057s0007.1.p
Mapoly0127s0010.1.p
Mapoly0217s0009.1.p
a
a
b
b
a
c
a
a
b
a
b
b
9.0
8.8
8.6
8.4
8.2
8.5
8.0
7.5
Abundance in log10
1
2
3
condition

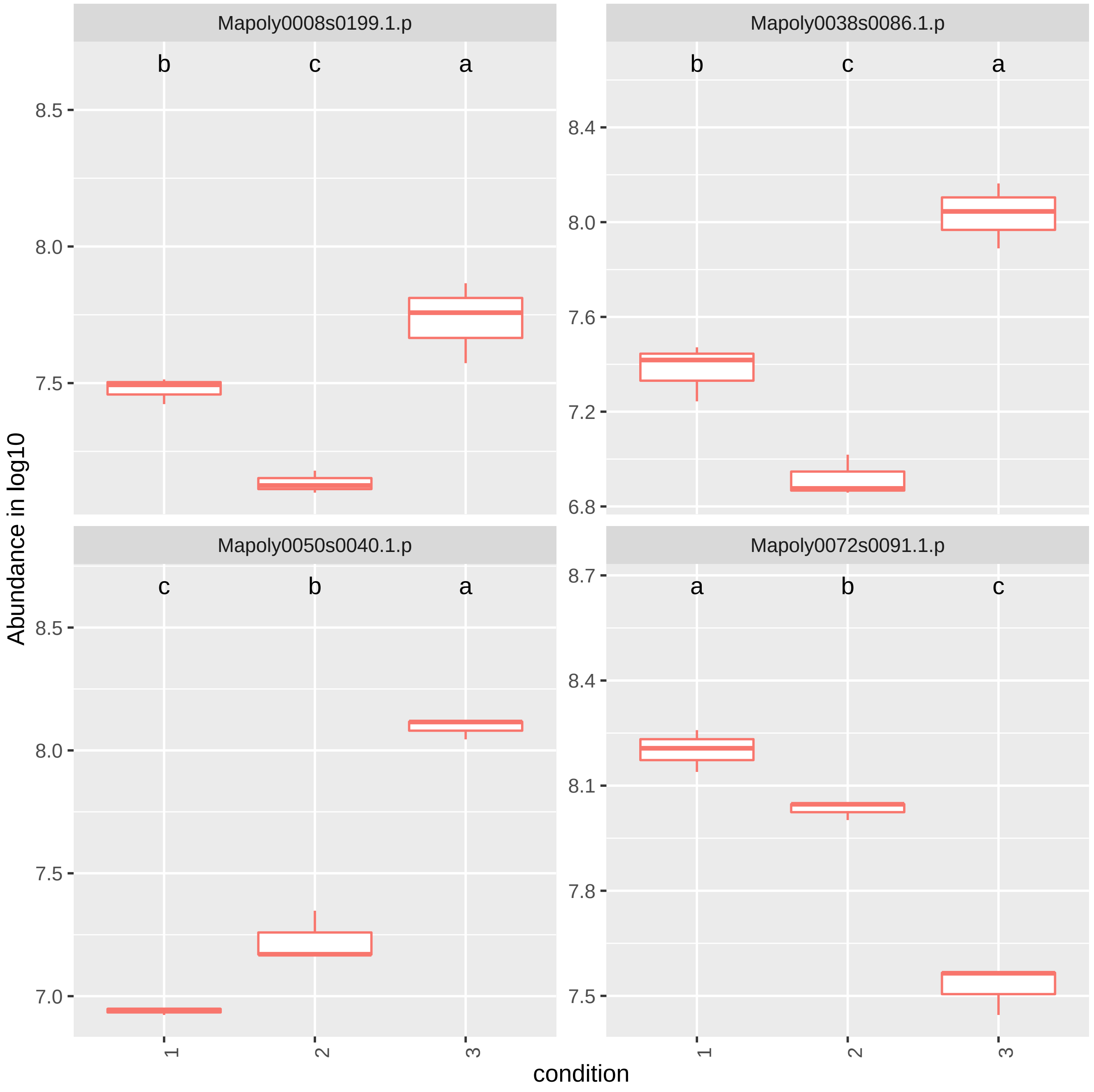

Mapoly0008s0199.1.p
Mapoly0038s0086.1.p
Mapoly0050s0040.1.p
Mapoly0072s0091.1.p
b c a
b c a
c b a
a b c
8.5
8.0
7.5
8.4
8.0
7.6
7.2
6.8
8.5
8.0
7.5
7.0
8.7
8.4
8.1
7.8
7.5
1 2 3
1 2 3
Abundance in log10
condition

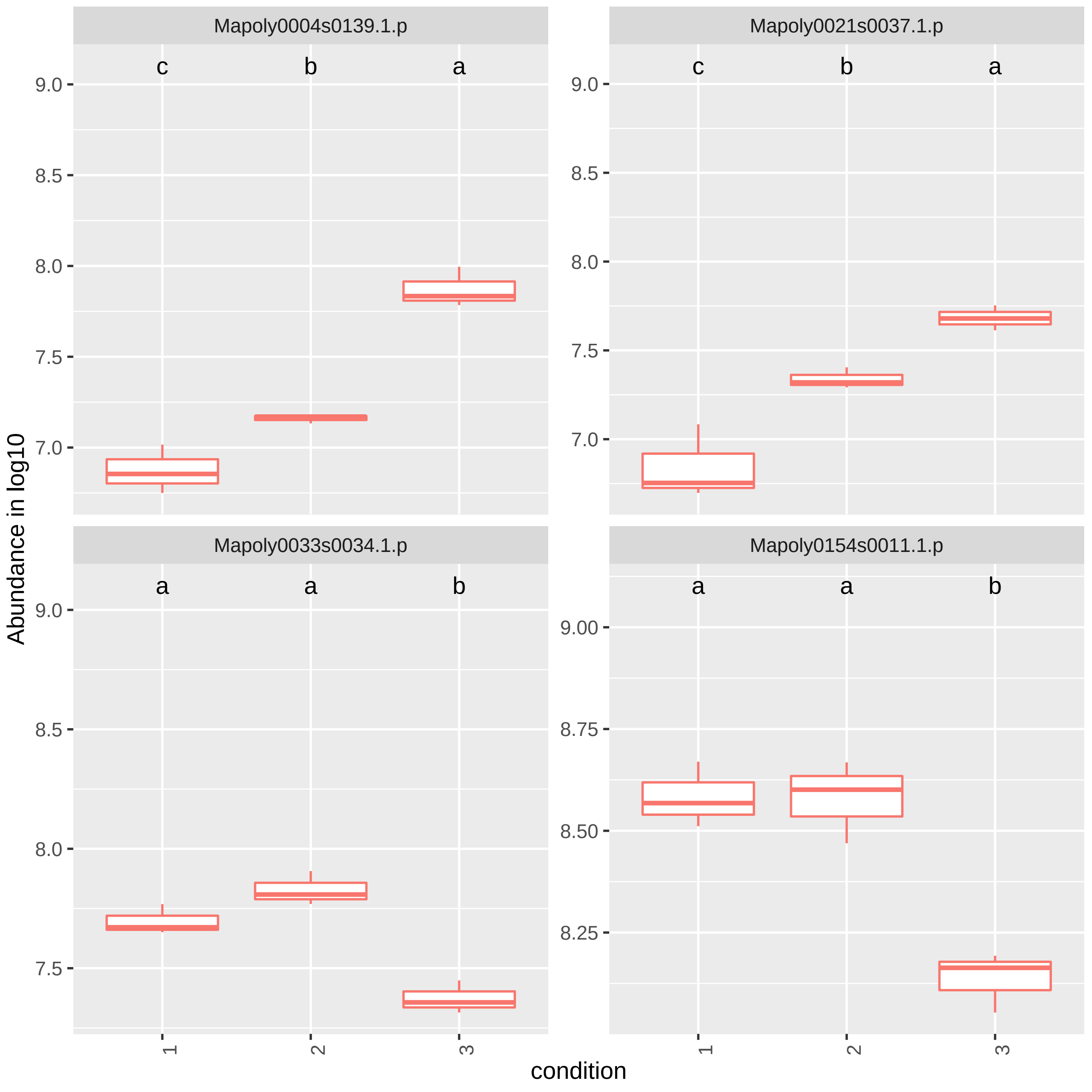

Mapoly0004s0139.1.p
Mapoly0021s0037.1.p
Mapoly0033s0034.1.p
Mapoly0154s0011.1.p
c
b
a
c
b
a
a
a
b
a
a
b
9.0
8.5
8.0
7.5
7.0
9.00
8.75
8.50
8.25
1
2
3
Abundance in log10
condition

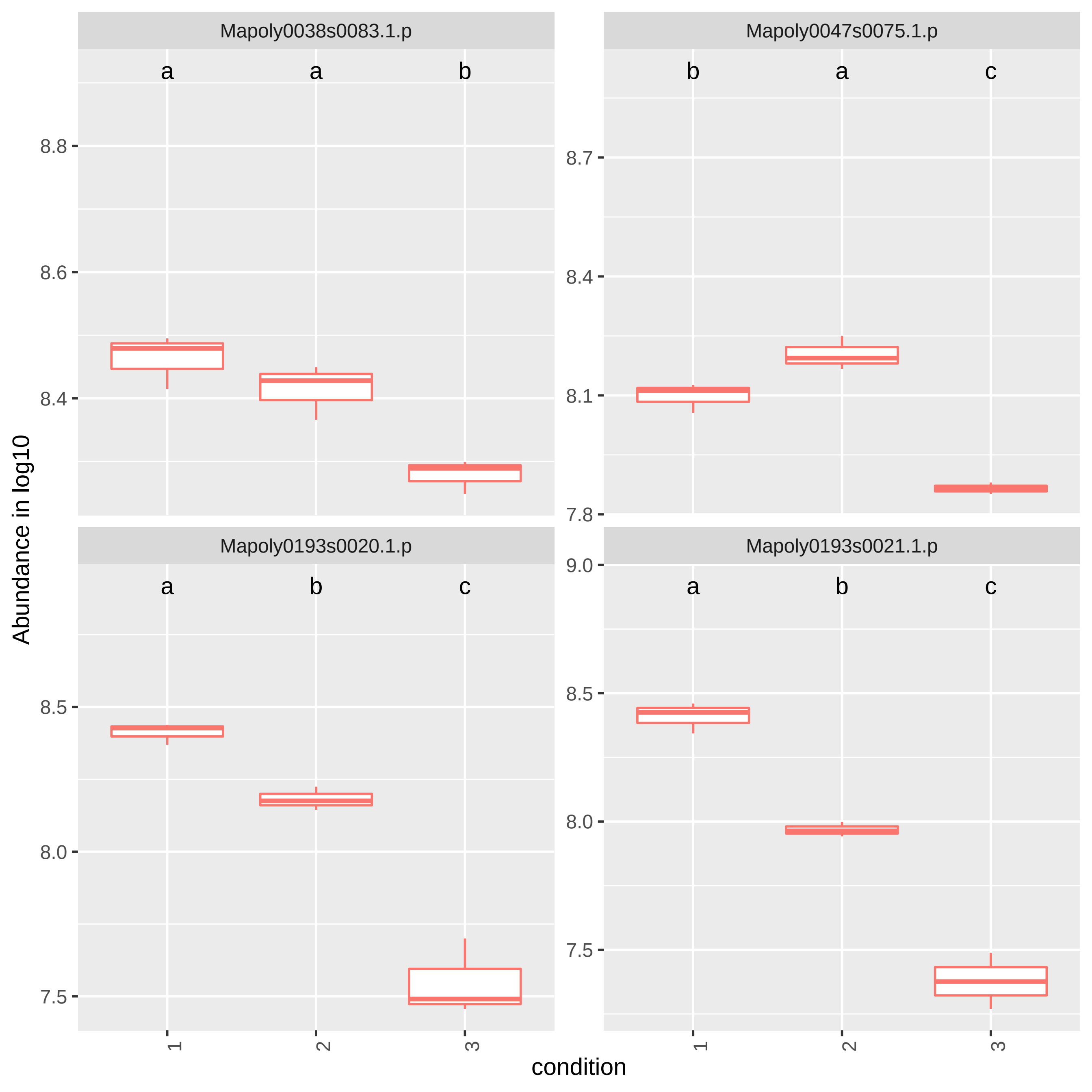

Mapoly0038s0083.1.p
Mapoly0047s0075.1.p
Mapoly0193s0020.1.p
Mapoly0193s0021.1.p
a a b
b a c
a b c
a b c
8.8
8.6
8.4
8.7
8.4
8.1
7.8
8.5
8.0
7.5
9.0
8.5
8.0
7.5
1 2 3
1 2 3
Abundance in log10
condition

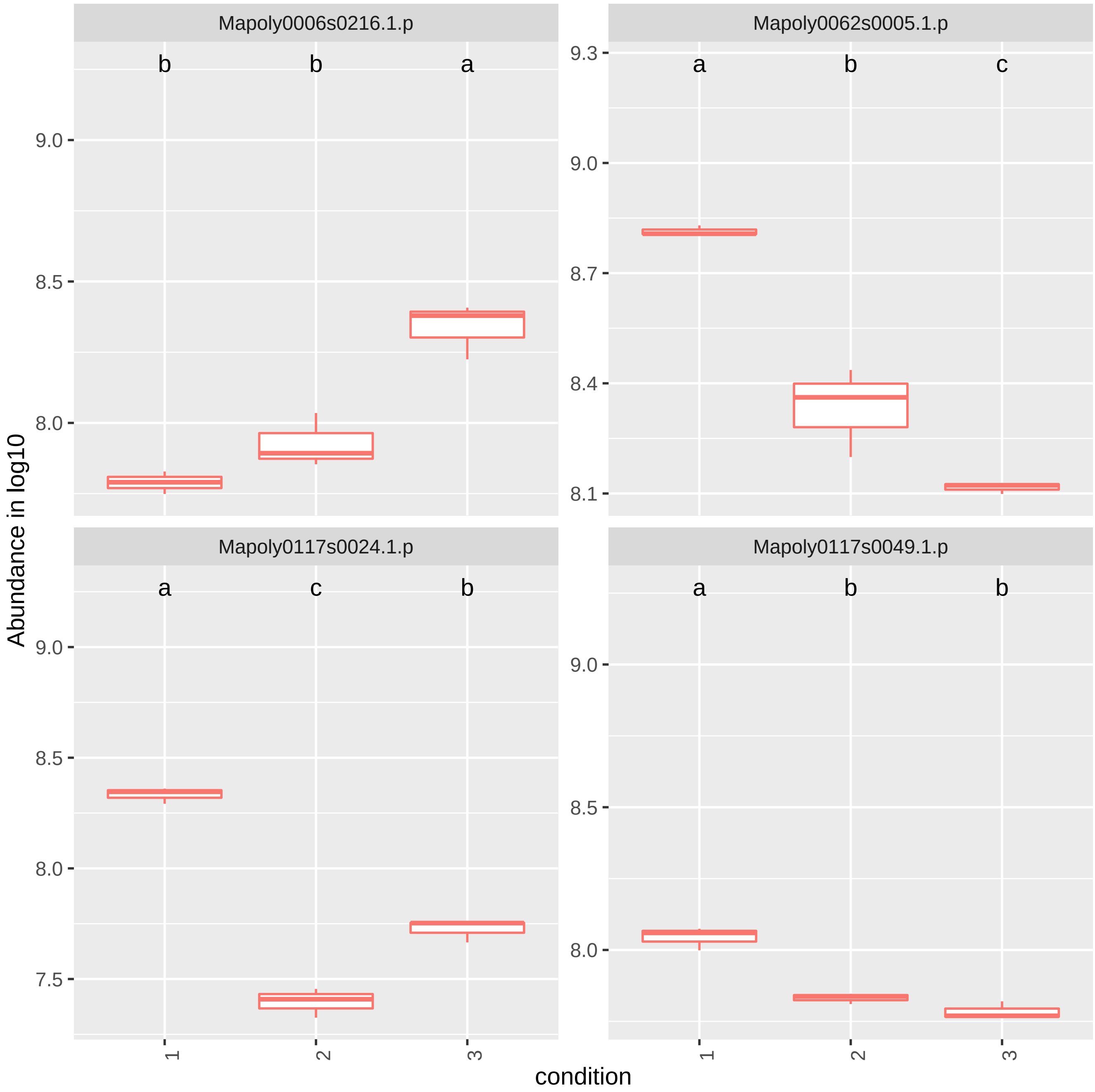

Mapoly0006s0216.1.p
Mapoly0062s0005.1.p
Mapoly0117s0024.1.p
Mapoly0117s0049.1.p
b
b
a
a
b
c
a
c
b
a
b
b
9.0
8.5
8.0
9.3
9.0
8.7
8.4
8.1
9.0
8.5
8.0
7.5
9.0
8.5
8.0
1
2
3
1
2
3
Abundance in log10
condition

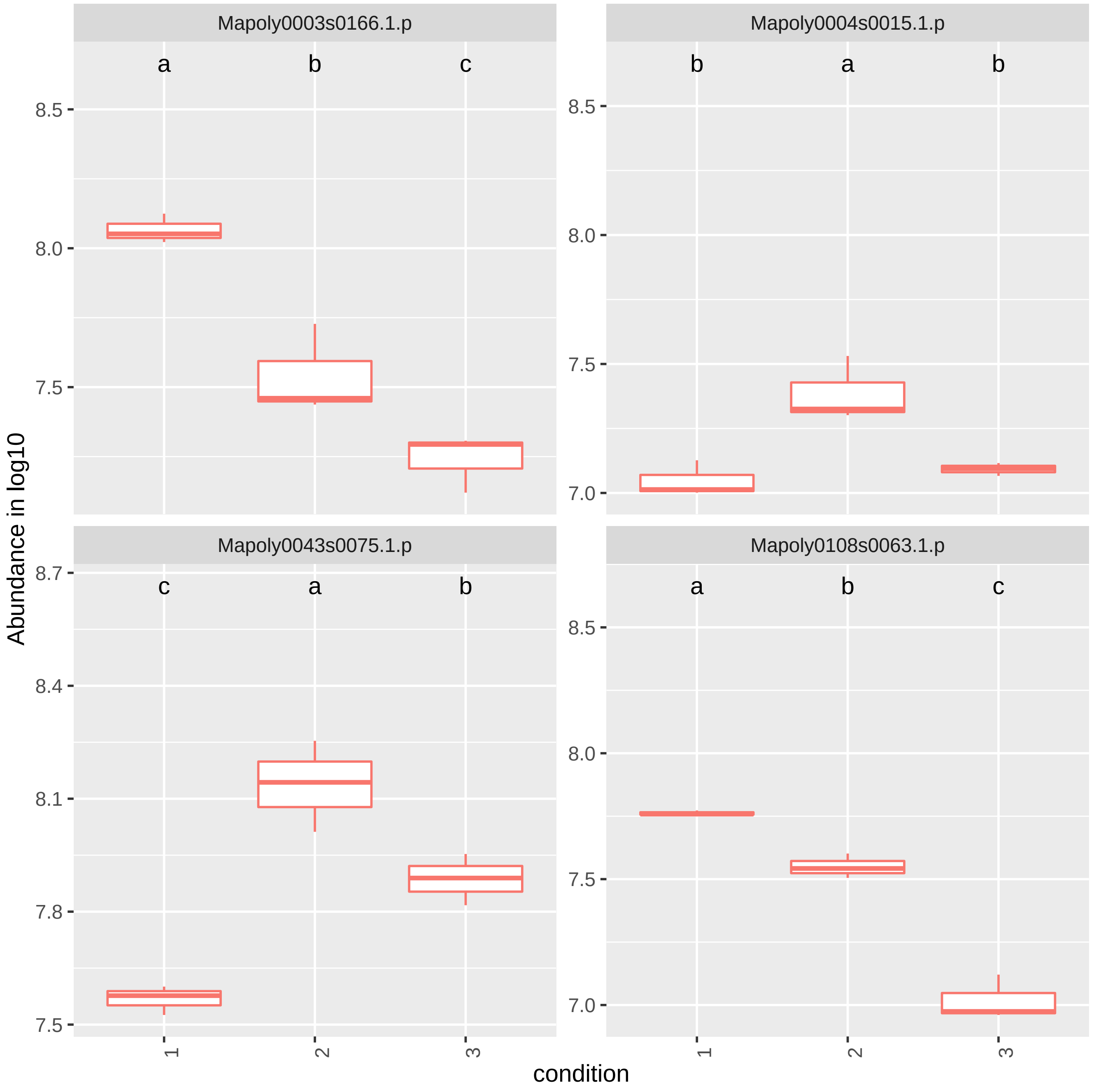

Mapoly0003s0166.1.p
Mapoly0004s0015.1.p
Mapoly0043s0075.1.p
Mapoly0108s0063.1.p
a b c
b a b
c a b
a b c
8.5
8.0
7.5
7.0
8.7
8.4
8.1
7.8
1
2
3
Abundance in log10
condition

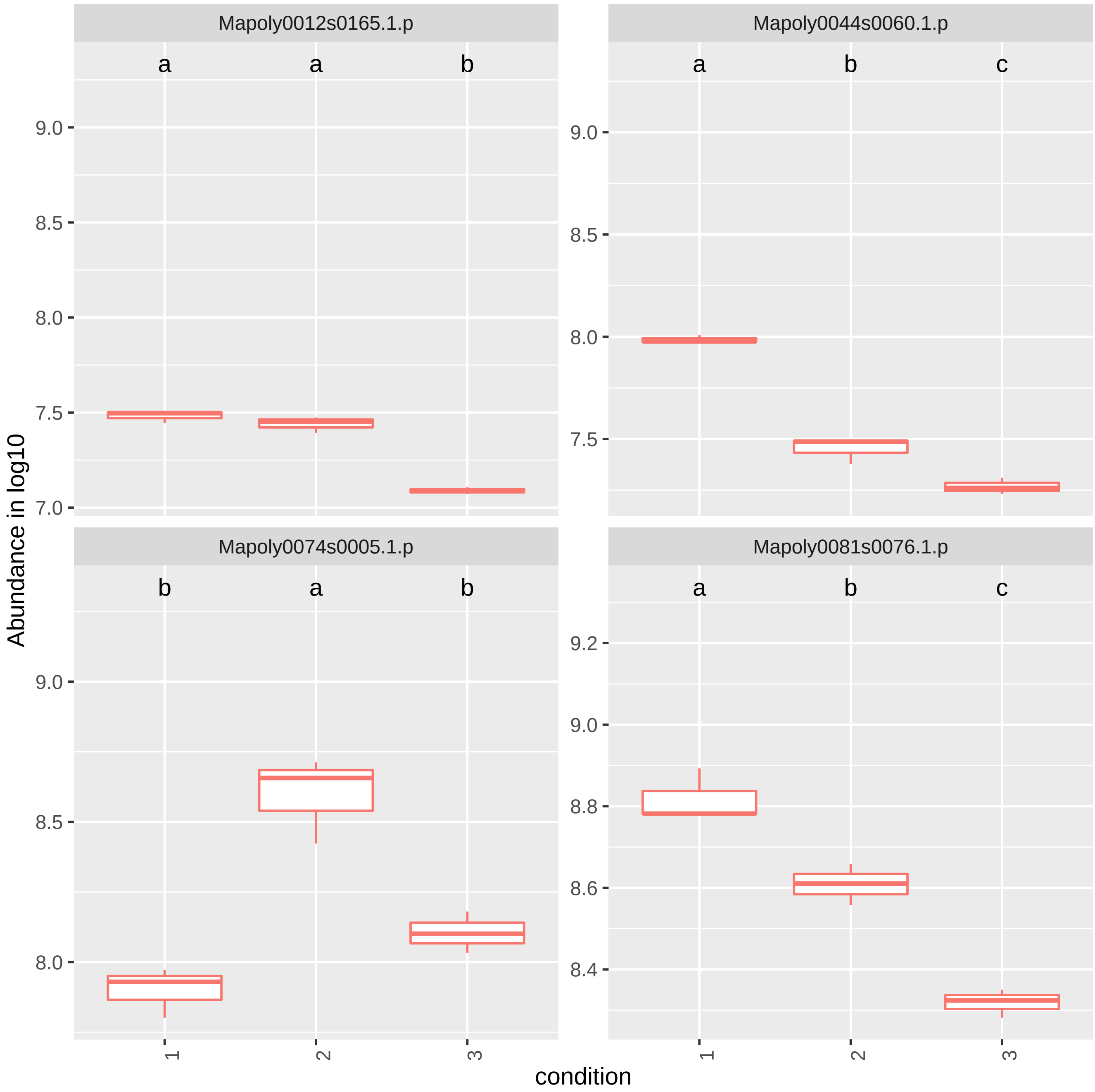

Mapoly0012s0165.1.p
Mapoly0044s0060.1.p
Mapoly0074s0005.1.p
Mapoly0081s0076.1.p
a a b
a b c
b a b
a b c
9.0
8.5
8.0
7.5
7.0
9.2
8.8
8.6
8.4
Abundance in log10
1 2 3
condition

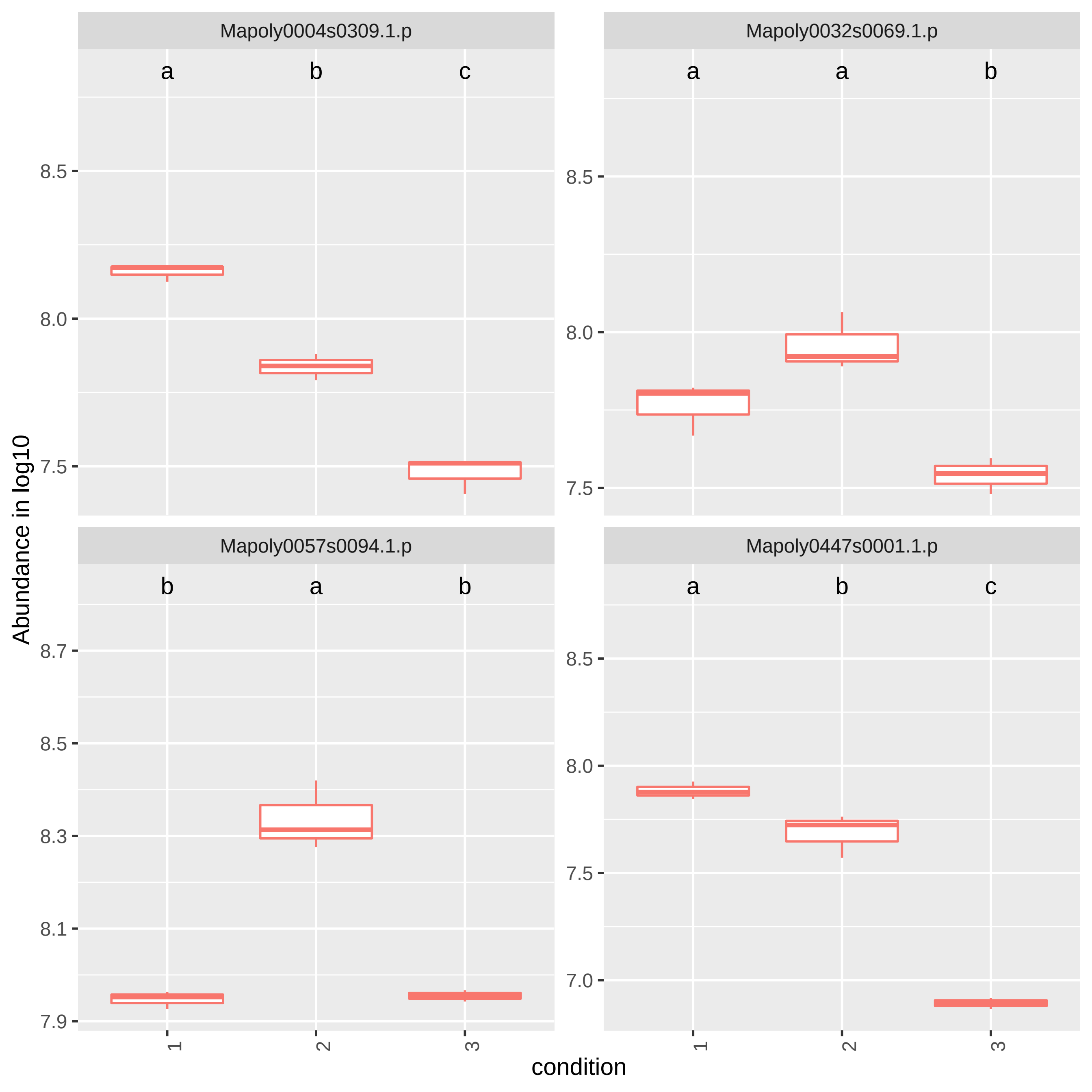
Mapoly0004s0309.1.p
Mapoly0032s0069.1.p
Mapoly0057s0094.1.p
Mapoly0447s0001.1.p
a
b
c
a
a
b
b
a
b
a
b
c
8.5
8.0
7.5
8.7
8.5
8.3
8.1
7.9
7.0
1
2
3
Abundance in log10
condition

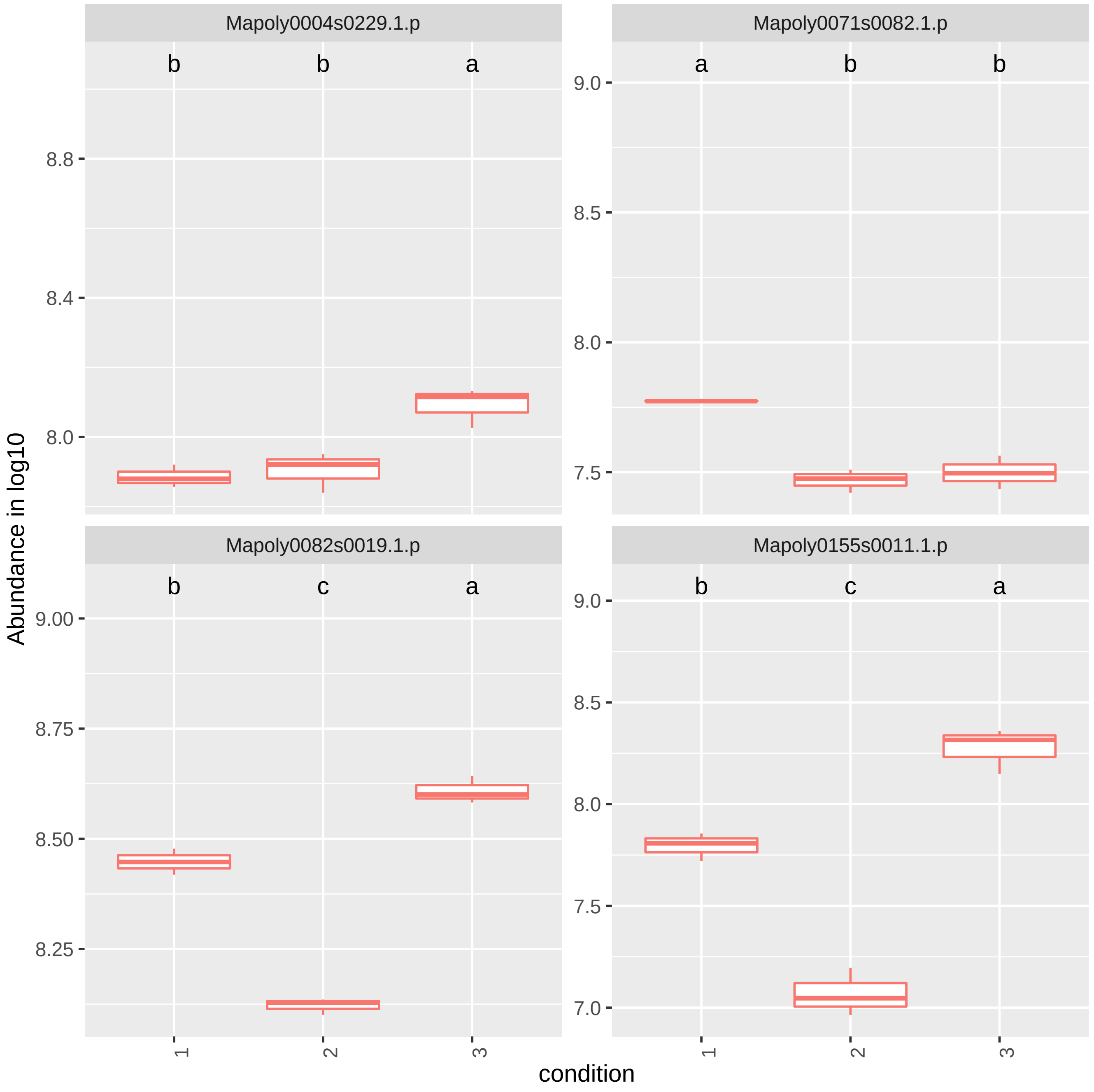
Mapoly0004s0229.1.p
Mapoly0071s0082.1.p
Mapoly0082s0019.1.p
Mapoly0155s0011.1.p
b b a
a b b
b c a
b c a
8.8
8.4
8.0
9.0
8.5
8.0
7.5
9.00
8.75
8.50
8.25
9.0
8.5
8.0
7.5
7.0
1 2 3
1 2 3
Abundance in log10
condition

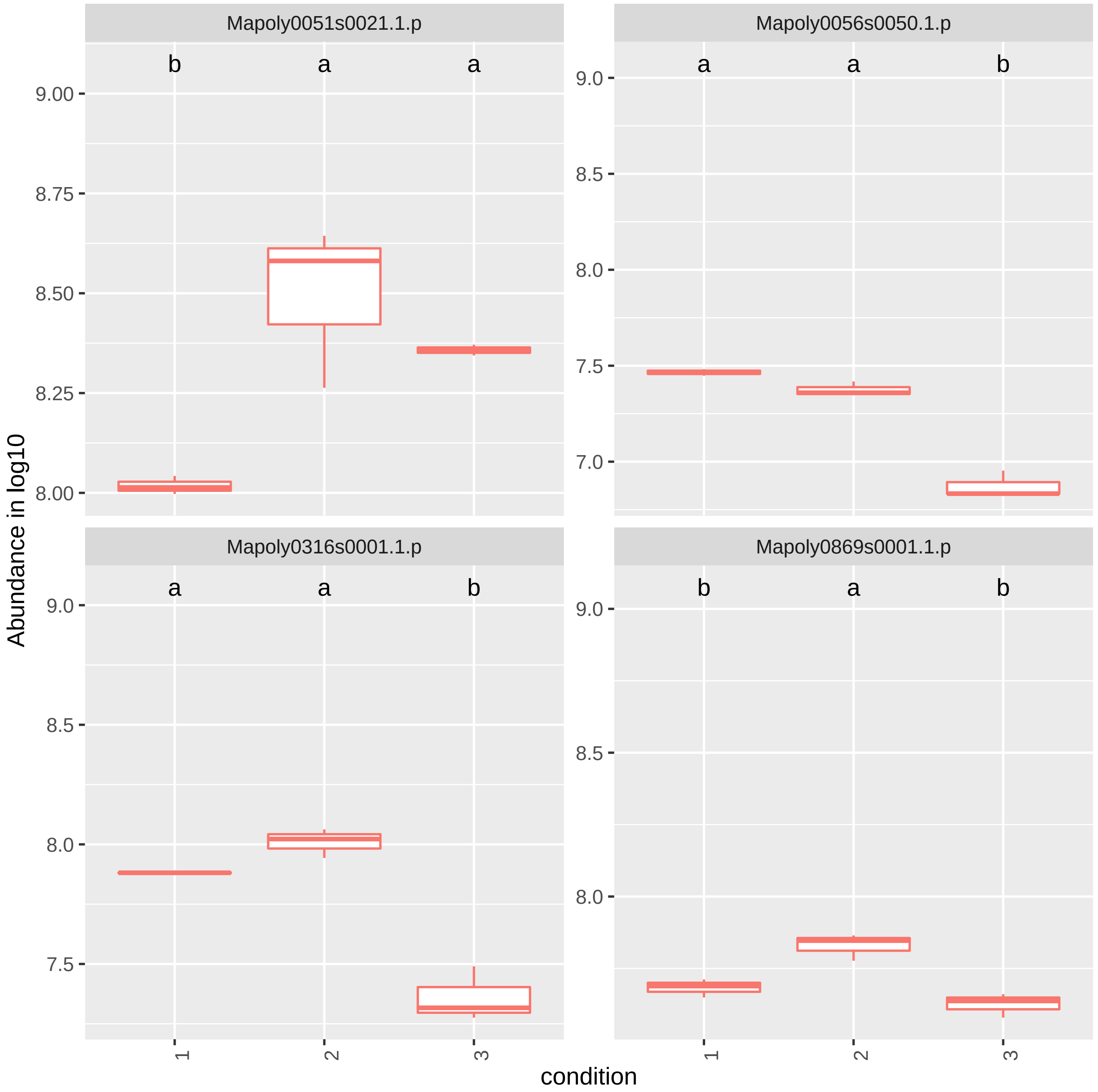

Mapoly0051s0021.1.p
Mapoly0056s0050.1.p
Mapoly0316s0001.1.p
Mapoly0869s0001.1.p
b a a
a a b
a a b
b a b
9.00
8.75
8.50
8.25
8.00
9.0
8.5
8.0
7.5
7.0
1 2 3
Abundance in log10
condition

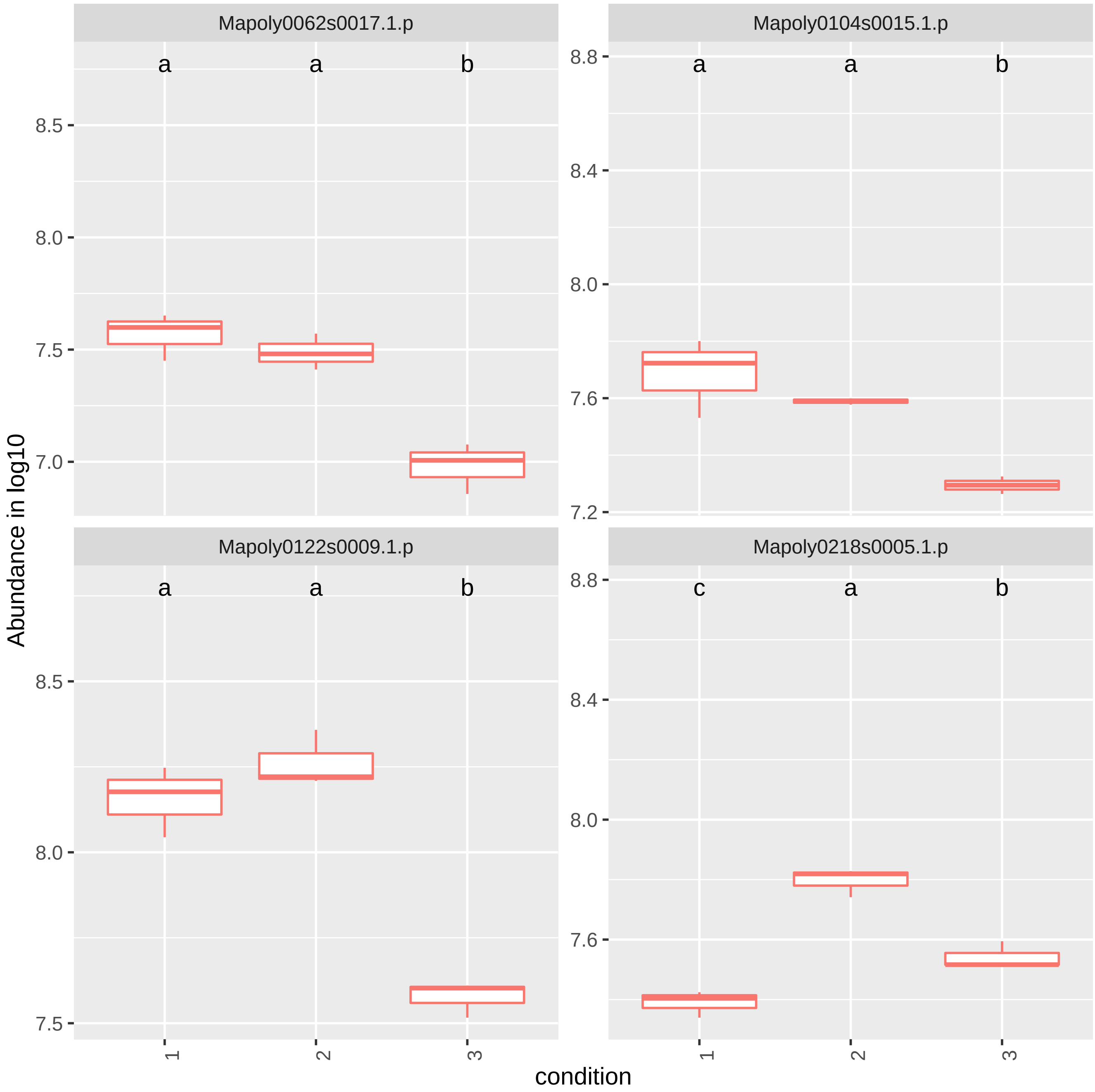

Mapoly0062s0017.1.p
Mapoly0104s0015.1.p
Mapoly0122s0009.1.p
Mapoly0218s0005.1.p
a
a
b
a
a
b
a
a
b
c
a
b
8.5
8.0
7.5
7.0
8.8
8.4
8.0
7.6
7.2
Abundance in log10
condition
1
2
3

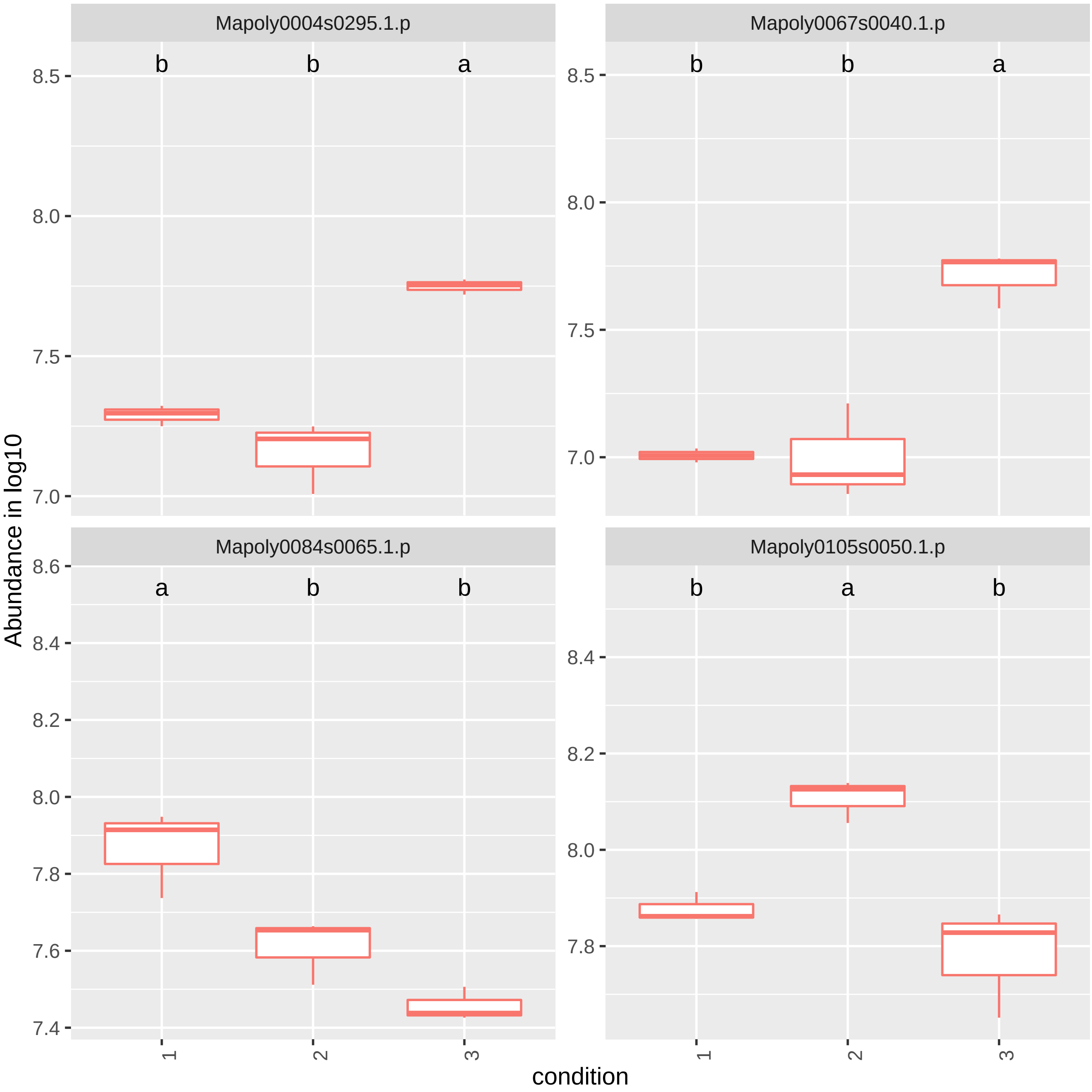

Mapoly0004s0295.1.p
Mapoly0067s0040.1.p
Mapoly0084s0065.1.p
Mapoly0105s0050.1.p
b b a
b b a
a b b
b a b
8.5
8.0
7.5
7.0
8.6
8.4
8.2
7.8
7.6
7.4
Abundance in log10
1
2
3
condition

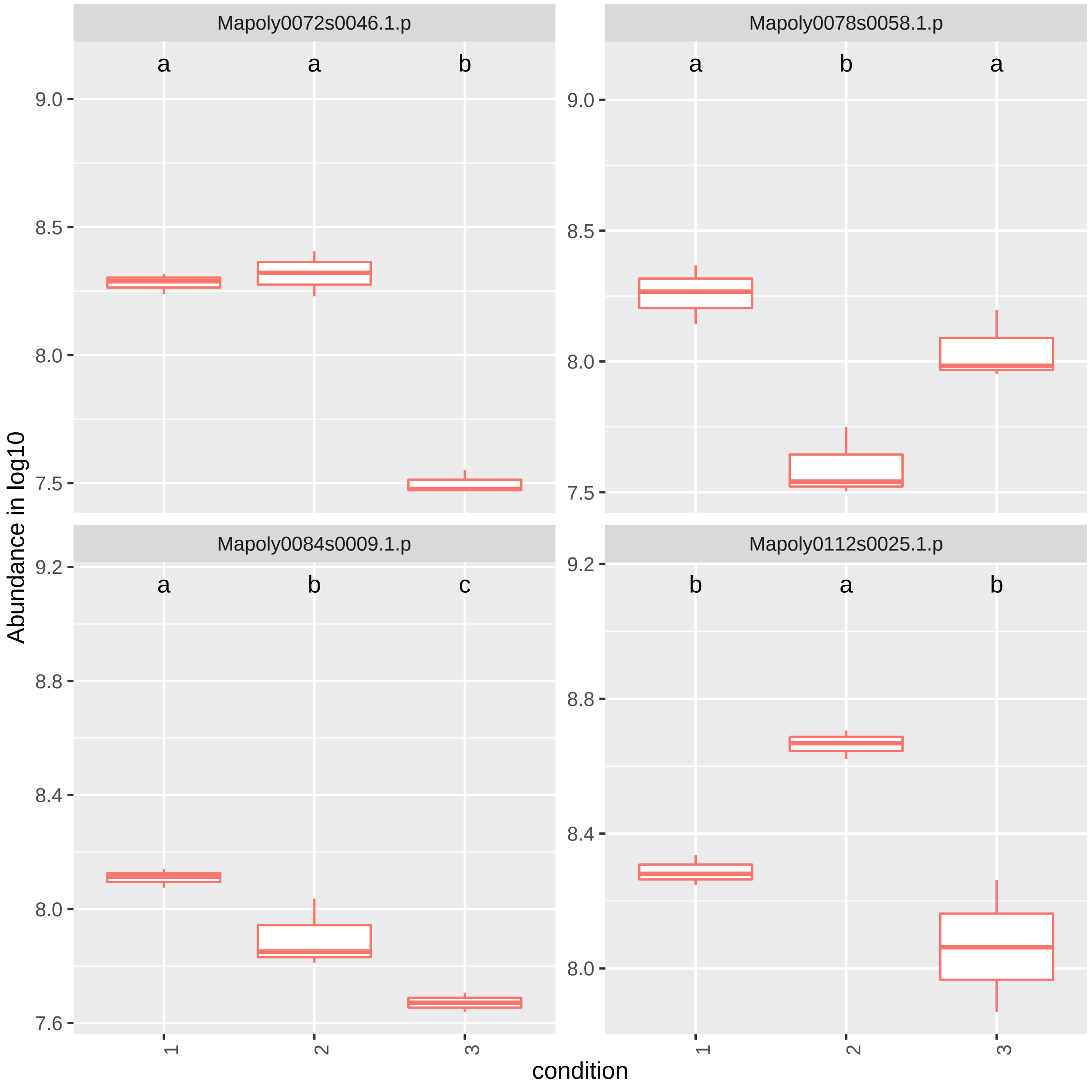

Mapoly0072s0046.1.p
Mapoly0078s0058.1.p
Mapoly0084s0009.1.p
Mapoly0112s0025.1.p
a
a
b
a
b
a
a
b
c
b
a
b
9.0
8.5
8.0
7.5
9.2
8.8
8.4
7.6
Abundance in log10
1
2
3
condition

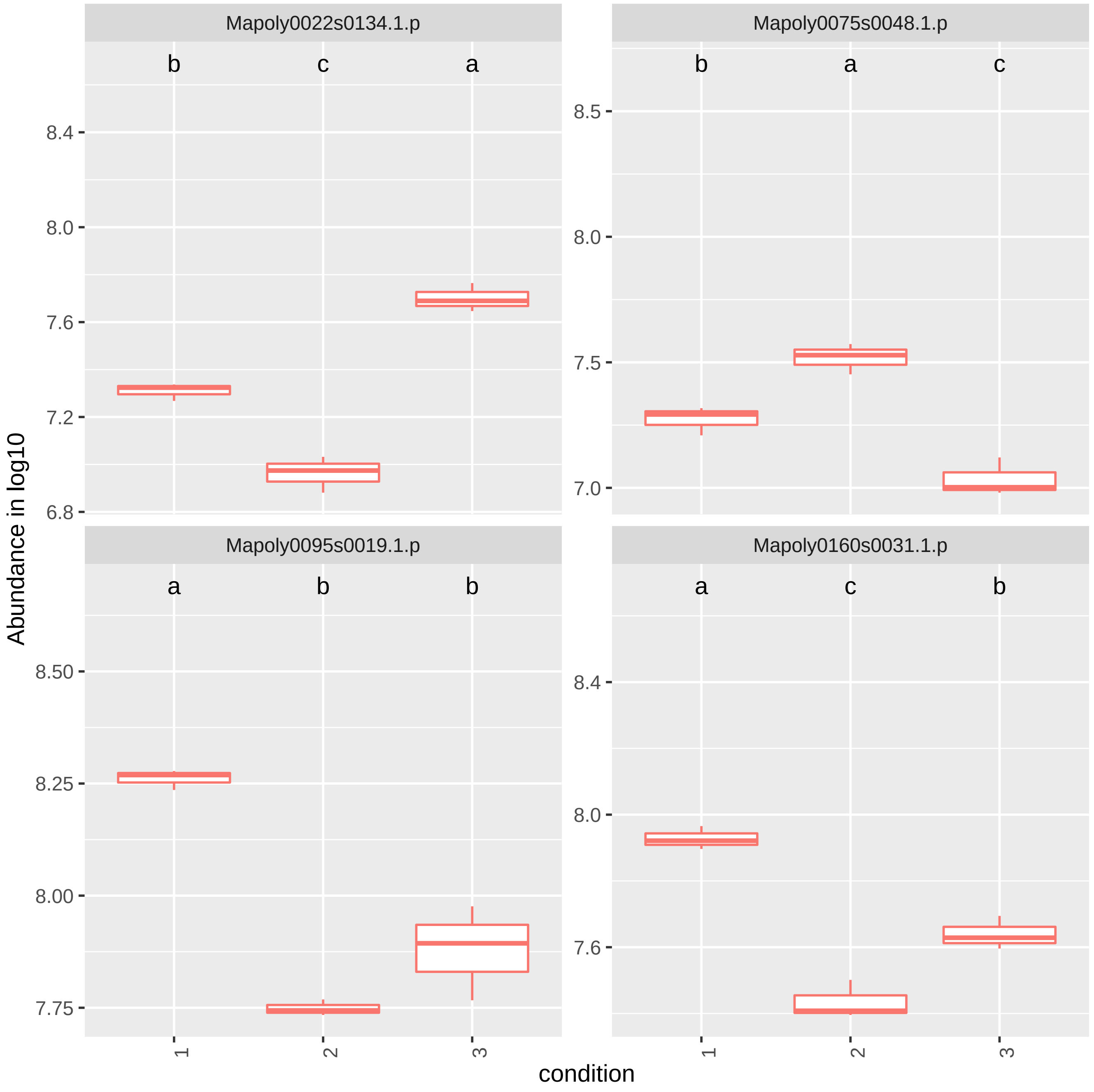

Mapoly0022s0134.1.p
Mapoly0075s0048.1.p
Mapoly0095s0019.1.p
Mapoly0160s0031.1.p
b c a
b a c
a b b
a c b
8.4
8.0
7.6
7.2
6.8
8.5
8.0
7.5
7.0
8.50
8.25
8.00
7.75
8.4
8.0
7.6
Abundance in log10
1 2 3
1 2 3
condition

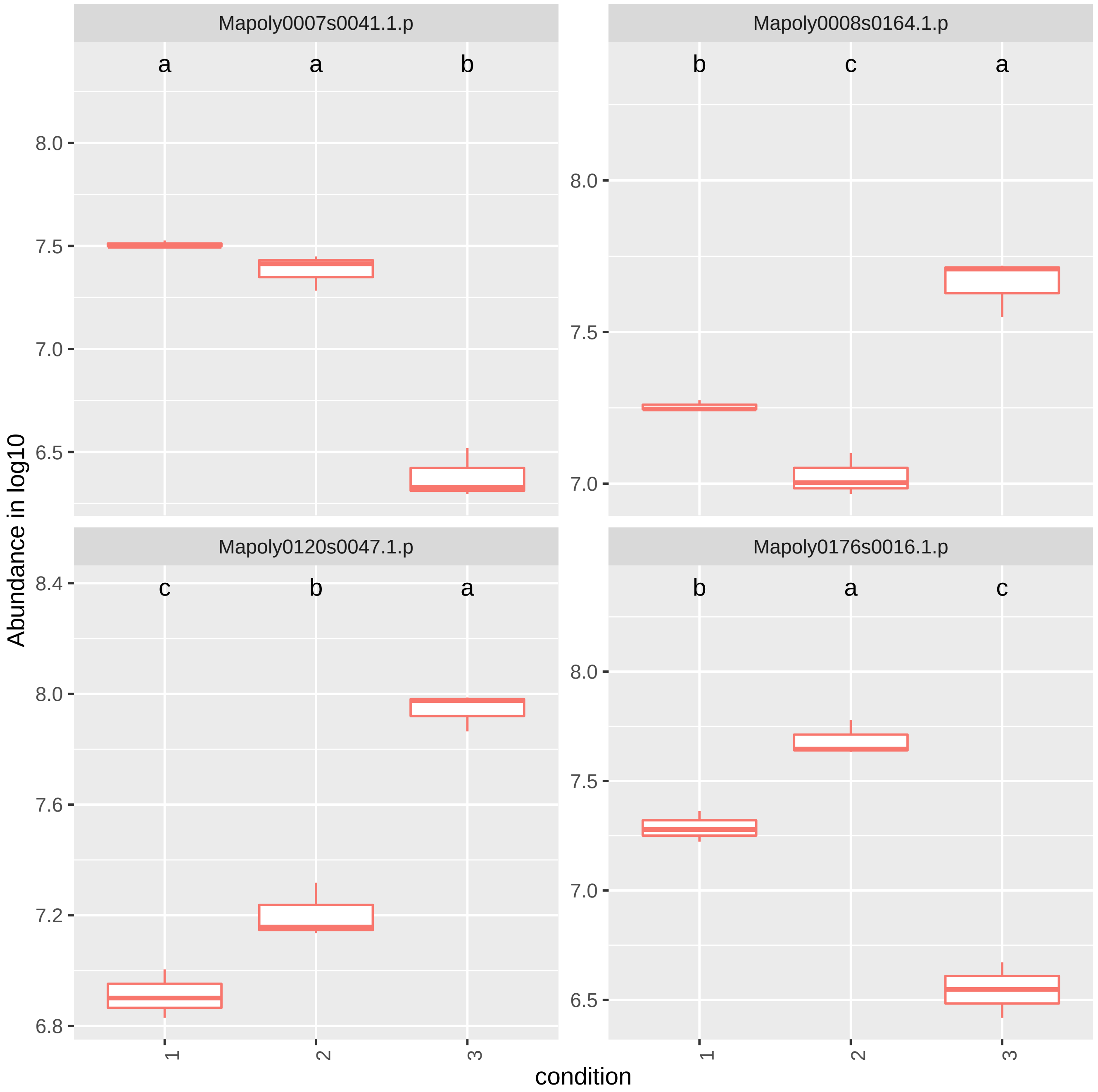

Mapoly0007s0041.1.p
Mapoly0008s0164.1.p
Mapoly0120s0047.1.p
Mapoly0176s0016.1.p
a
a
b
b
c
a
c
b
a
b
a
c
8.0
7.5
7.0
6.5
8.0
7.5
7.0
8.4
8.0
7.6
7.2
6.8
8.0
7.5
7.0
6.5
1
2
3
1
2
3
Abundance in log10
condition

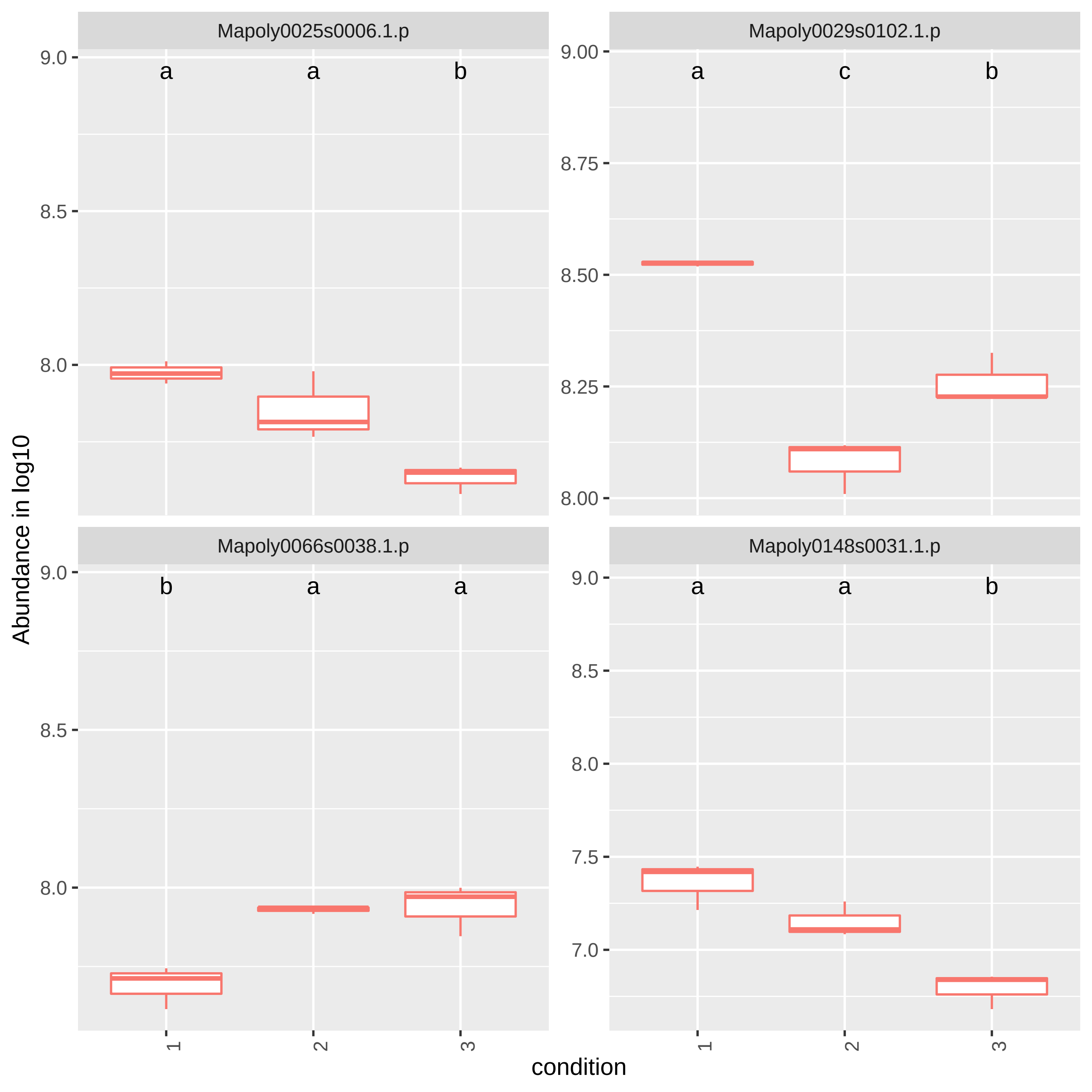

Mapoly0025s0006.1.p
Mapoly0029s0102.1.p
Mapoly0066s0038.1.p
Mapoly0148s0031.1.p
a a b
a c b
b a a
a a b
9.0
8.5
8.0
9.00
8.75
8.50
8.25
8.00
7.5
7.0
1 2 3
Abundance in log10
condition

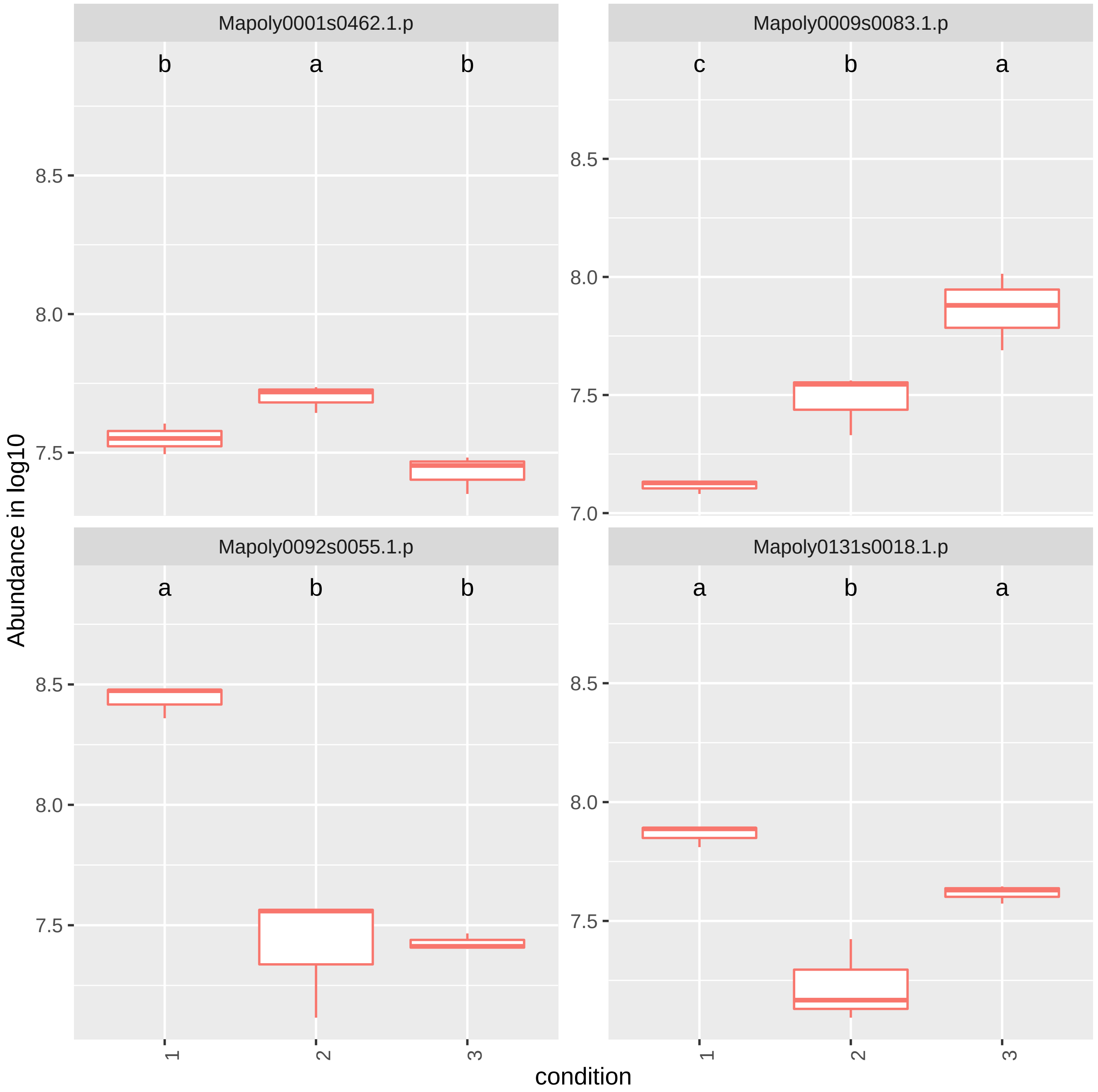
Mapoly0001s0462.1.p
b
a
b
Mapoly0009s0083.1.p
c
b
a
Mapoly0092s0055.1.p
a
b
b
Mapoly0131s0018.1.p
a
b
a
8.5
8.0
7.5
7.0
1
2
3
Abundance in log10
condition

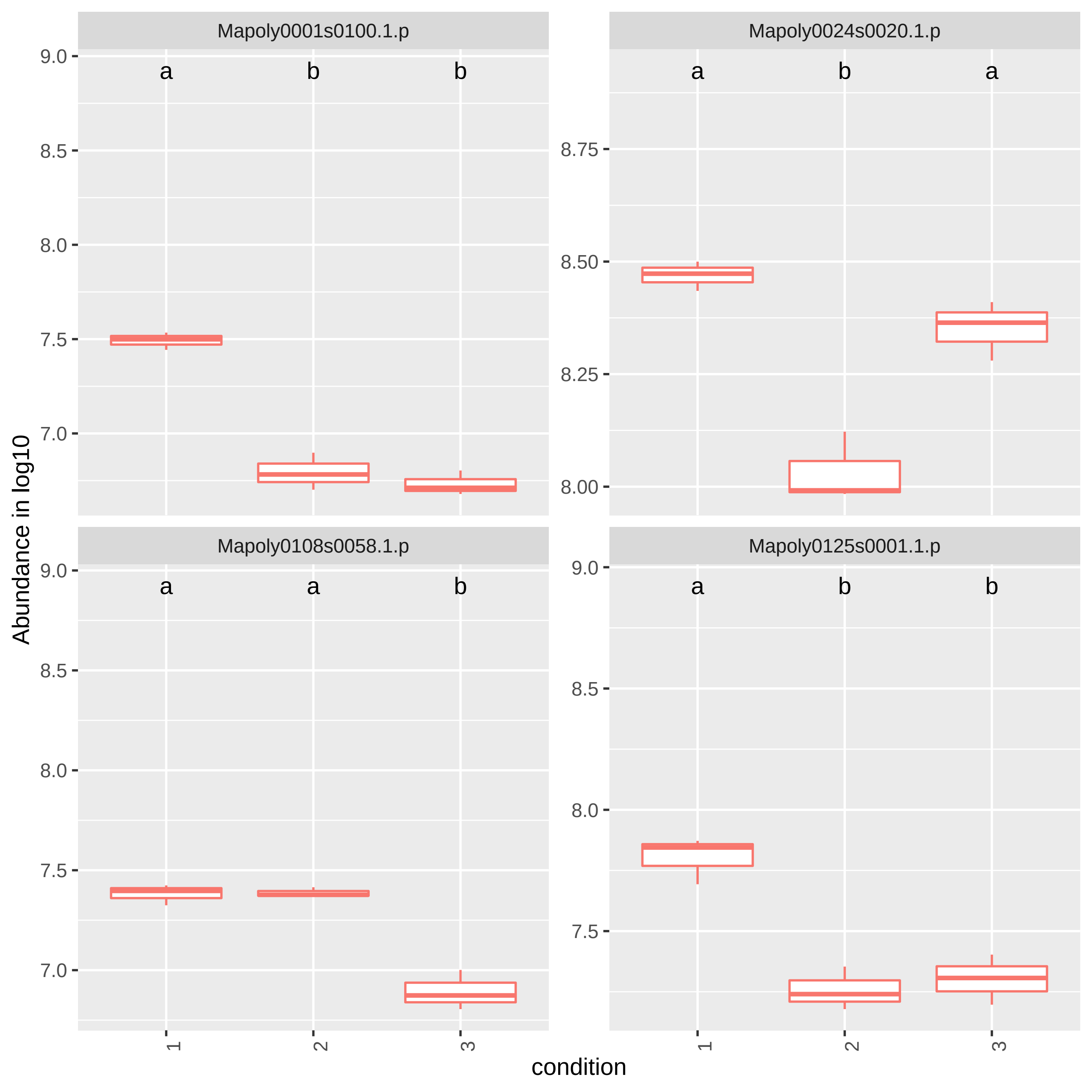
Mapoly0001s0100.1.p
Mapoly0024s0020.1.p
Mapoly0108s0058.1.p
Mapoly0125s0001.1.p
a
b
b
a
b
a
a
a
b
a
b
b
9.0
8.5
8.0
7.5
7.0
8.75
8.50
8.25
8.00
Abundance in log10
1
2
3
condition

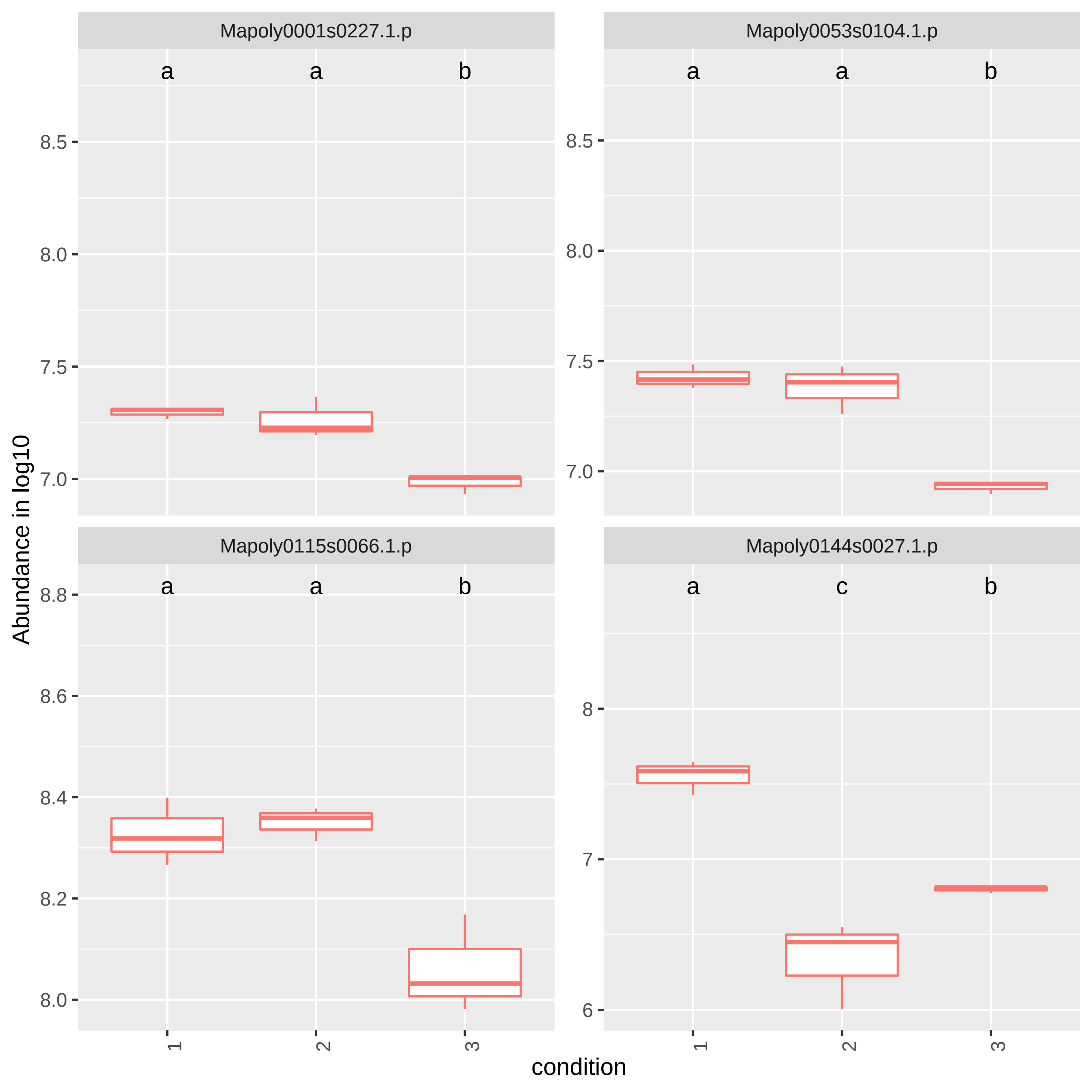
Mapoly0001s0227.1.p
Mapoly0053s0104.1.p
Mapoly0115s0066.1.p
Mapoly0144s0027.1.p
a
a
b
a
a
b
a
a
b
a
c
b
8.5
8.0
7.5
7.0
8.5
8.0
7.5
7.0
8.8
8.6
8.4
8.2
8.0
8
7
6
1
2
3
1
2
3
Abundance in log10
condition

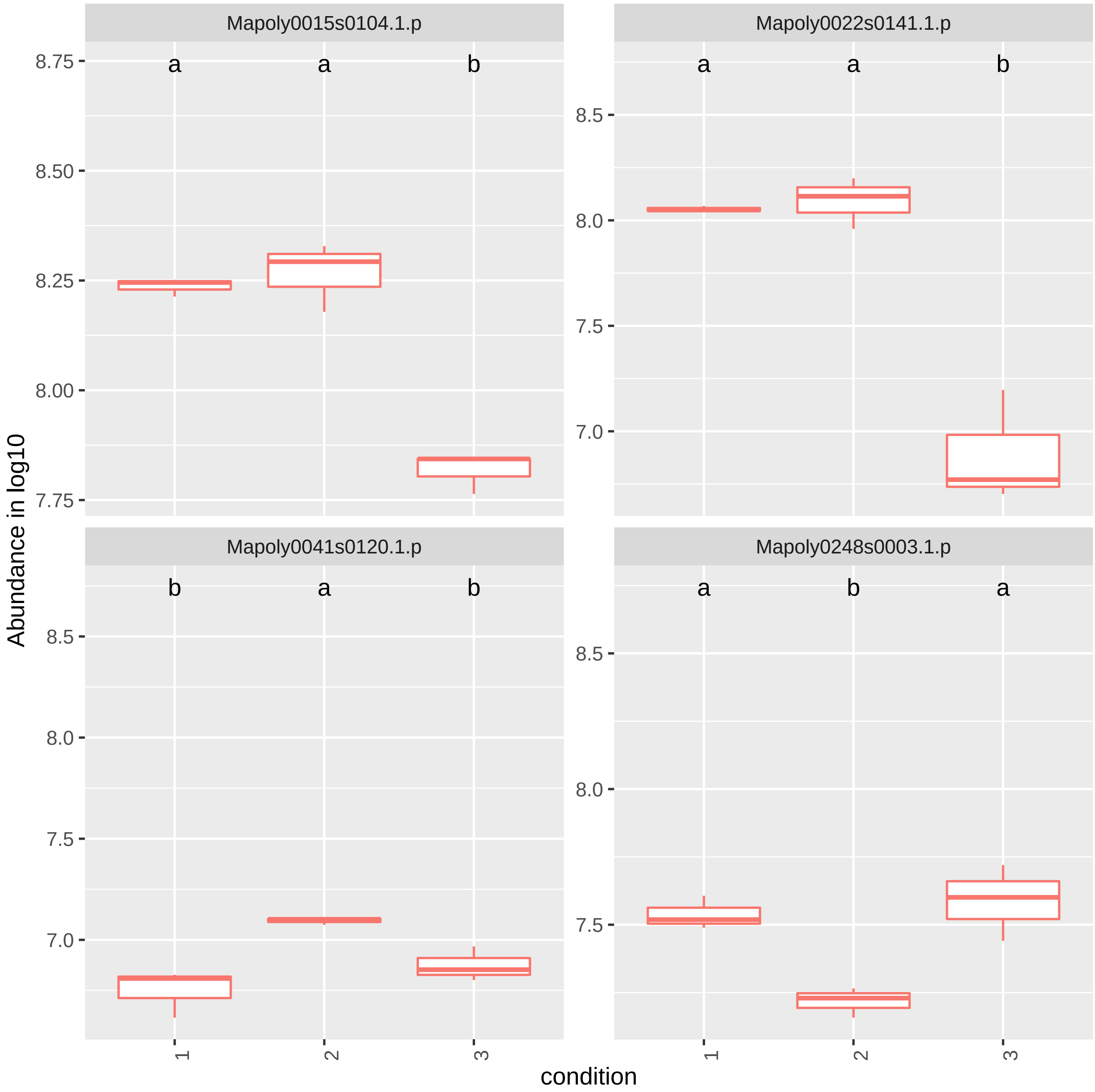

Mapoly0015s0104.1.p
Mapoly0022s0141.1.p
Mapoly0041s0120.1.p
Mapoly0248s0003.1.p
a a b
a a b
b a b
a b a
8.75
8.50
8.25
8.00
7.75
8.5
8.0
7.5
7.0
Abundance in log10
1
2
3
condition

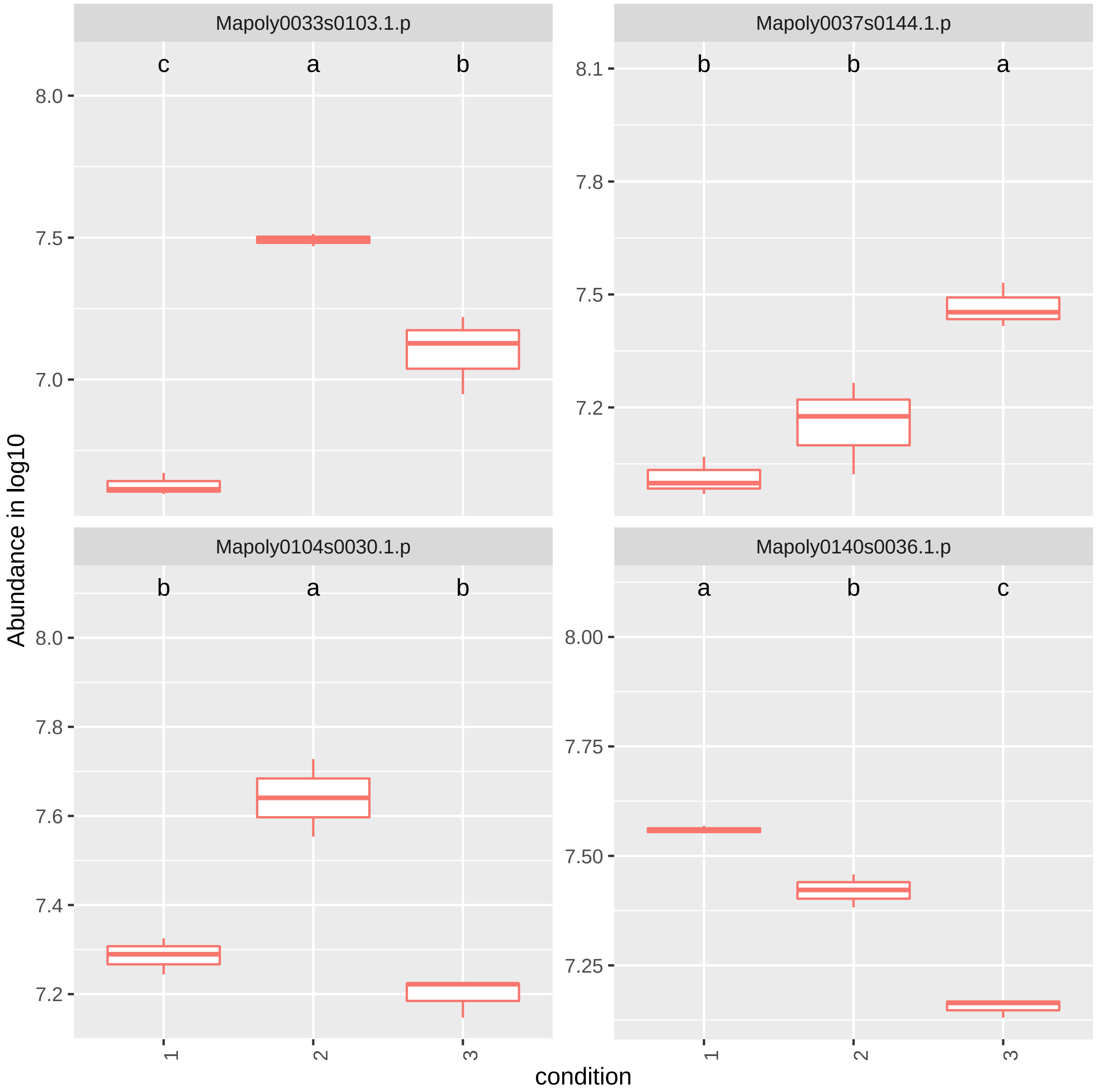

Mapoly0033s0103.1.p
Mapoly0037s0144.1.p
Mapoly0104s0030.1.p
Mapoly0140s0036.1.p
c
a
b
b
b
a
b
a
b
a
b
c
8.0
7.5
7.0
8.1
7.8
7.5
7.2
8.0
7.8
7.6
7.4
7.2
8.00
7.75
7.50
7.25
1
2
3
Abundance in log10
condition

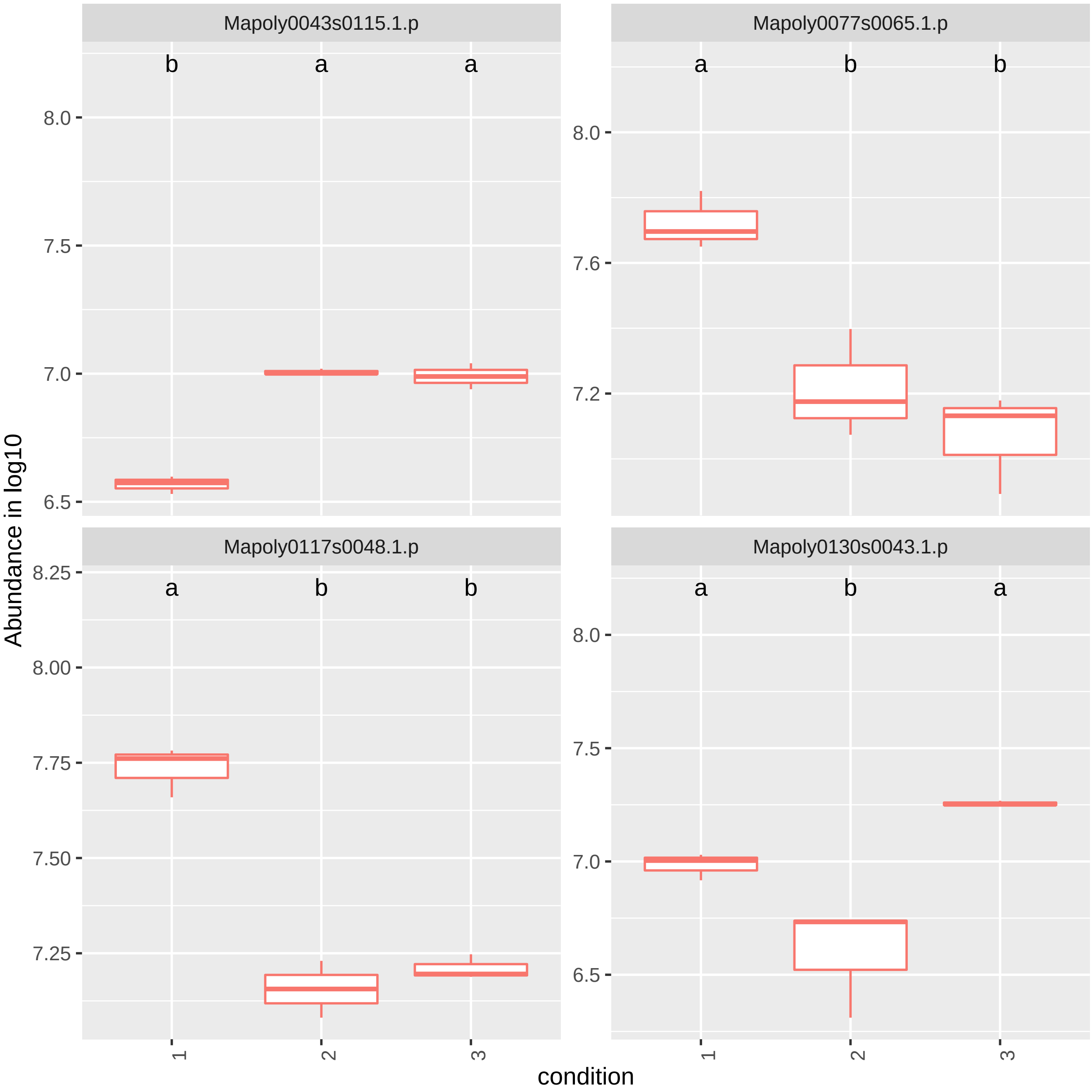
Mapoly0043s0115.1.p
b a a
8.0
7.5
7.0
6.5
Mapoly0077s0065.1.p
a b b
8.0
7.6
7.2
Mapoly0117s0048.1.p
a b b
8.25
8.00
7.75
7.50
7.25
Mapoly0130s0043.1.p
a b a
8.0
7.5
7.0
6.5
1
2
3
1
2
3
Abundance in log10
condition

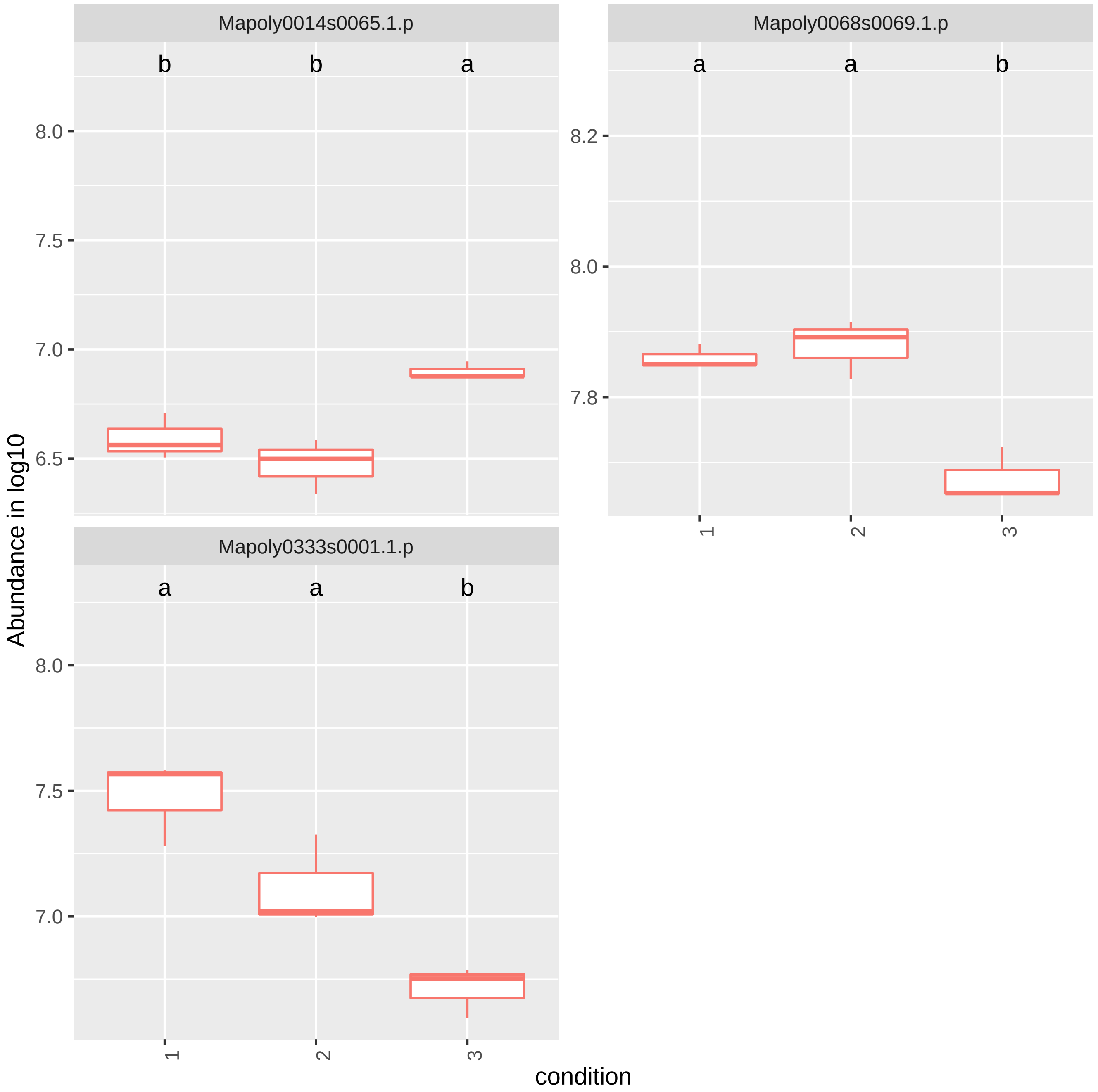
Mapoly0014s0065.1.p
Mapoly0068s0069.1.p
Mapoly0333s0001.1.p
b
b
a
a
a
b
a
a
b
8.0
7.5
7.0
6.5
8.2
8.0
7.8
8.0
7.5
7.0
1
2
3
1
2
3
Abundance in log10
condition

Quantification of the CWPs proteins using extracted ion chromatograms (XICs)
1 stands for 2 week-old thalli / **2** stands for 3 week-old thalli / **3** stands for 5 week-old thalli

accession	ratio 2/1	ratio 1/2	accession	ratio 3/2	ratio 2/3	accession	ratio 3/1	ratio 1/3
Mapoly1035s0001	2,19	0,46	Mapoly1035s0001	15,43	0,06	Mapoly1035s0001	33,83	0,03
Mapoly0342s0001	1,26	0,79	Mapoly0342s0001	19,79	0,05	Mapoly0342s0001	24,95	0,04
Mapoly0121s0006	2,58	0,39	Mapoly0121s0006	7,37	0,14	Mapoly0121s0006	19,00	0,05
Mapoly0008s0232	1,07	0,93	Mapoly0008s0232	17,14	0,06	Mapoly0008s0232	18,39	0,05
Mapoly0130s0001	2,84	0,35	Mapoly0130s0001	6,10	0,16	Mapoly0130s0001	17,32	0,06
Mapoly0121s0010	2,98	0,34	Mapoly0121s0010	5,07	0,20	Mapoly0121s0010	15,12	0,07
Mapoly0160s0003	0,68	1,46	Mapoly0160s0003	21,46	0,05	Mapoly0160s0003	14,68	0,07
Mapoly0050s0040	1,98	0,50	Mapoly0050s0040	7,19	0,14	Mapoly0050s0040	14,26	0,07
Mapoly0121s0008	3,44	0,29	Mapoly0121s0008	3,74	0,27	Mapoly0121s0008	12,87	0,08
Mapoly0120s0047	1,97	0,51	Mapoly0120s0047	5,42	0,18	Mapoly0120s0047	10,67	0,09
Mapoly0121s0009	3,26	0,31	Mapoly0121s0009	3,21	0,31	Mapoly0121s0009	10,46	0,10
Mapoly0048s0071	0,41	2,47	Mapoly0048s0071	25,79	0,04	Mapoly0048s0071	10,45	0,10
Mapoly0004s0139	1,87	0,53	Mapoly0004s0139	5,25	0,19	Mapoly0004s0139	9,85	0,10
Mapoly0088s0001	4,45	0,22	Mapoly0088s0001	1,63	0,62	Mapoly0088s0001	7,24	0,14
Mapoly0021s0037	2,89	0,35	Mapoly0021s0037	2,21	0,45	Mapoly0021s0037	6,39	0,16
Mapoly0009s0083	2,37	0,42	Mapoly0009s0083	2,45	0,41	Mapoly0009s0083	5,82	0,17
Mapoly0048s0056	1,18	0,85	Mapoly0048s0056	4,88	0,20	Mapoly0048s0056	5,78	0,17
Mapoly0212s0012	1,94	0,51	Mapoly0212s0012	2,79	0,36	Mapoly0212s0012	5,43	0,18
Mapoly0067s0040	1,05	0,95	Mapoly0067s0040	4,91	0,20	Mapoly0067s0040	5,14	0,19
Mapoly0044s0030	0,66	1,51	Mapoly0044s0030	7,32	0,14	Mapoly0044s0030	4,83	0,21
Mapoly0048s0002	1,51	0,66	Mapoly0048s0002	3,04	0,33	Mapoly0048s0002	4,61	0,22
Mapoly0038s0086	0,34	2,91	Mapoly0038s0086	13,28	0,08	Mapoly0038s0086	4,56	0,22
Mapoly0064s0029	2,62	0,38	Mapoly0064s0029	1,59	0,63	Mapoly0064s0029	4,16	0,24
Mapoly0037s0132	1,32	0,76	Mapoly0037s0132	3,14	0,32	Mapoly0037s0132	4,13	0,24
Mapoly0075s0023	3,38	0,30	Mapoly0075s0023	1,13	0,89	Mapoly0075s0023	3,81	0,26
Mapoly0006s0216	1,39	0,72	Mapoly0006s0216	2,57	0,39	Mapoly0006s0216	3,58	0,28
Mapoly0027s0087	2,38	0,42	Mapoly0027s0087	1,44	0,69	Mapoly0027s0087	3,44	0,29
Mapoly0002s0292	2,18	0,46	Mapoly0002s0292	1,48	0,67	Mapoly0002s0292	3,24	0,31
Mapoly0155s0011	0,19	5,23	Mapoly0155s0011	16,02	0,06	Mapoly0155s0011	3,06	0,33
Mapoly0033s0103	7,31	0,14	Mapoly0033s0103	0,42	2,40	Mapoly0033s0103	3,05	0,33
Mapoly0004s0295	0,75	1,33	Mapoly0004s0295	3,84	0,26	Mapoly0004s0295	2,88	0,35
Mapoly0037s0144	1,42	0,71	Mapoly0037s0144	2,01	0,50	Mapoly0037s0144	2,85	0,35
Mapoly0043s0115	2,74	0,37	Mapoly0043s0115	0,97	1,03	Mapoly0043s0115	2,65	0,38
Mapoly0008s0164	0,59	1,69	Mapoly0008s0164	4,34	0,23	Mapoly0008s0164	2,57	0,39
Mapoly0022s0134	0,45	2,21	Mapoly0022s0134	5,45	0,18	Mapoly0022s0134	2,47	0,41
Mapoly0093s0009	2,52	0,40	Mapoly0093s0009	0,98	1,03	Mapoly0093s0009	2,45	0,41
Mapoly0196s0008	0,54	1,86	Mapoly0196s0008	4,53	0,22	Mapoly0196s0008	2,44	0,41
Mapoly0009s0184	2,87	0,35	Mapoly0009s0184	0,84	1,19	Mapoly0009s0184	2,42	0,41
Mapoly0212s0013	3,40	0,29	Mapoly0212s0013	0,71	1,41	Mapoly0212s0013	2,41	0,42
Mapoly0048s0057	3,28	0,31	Mapoly0048s0057	0,72	1,39	Mapoly0048s0057	2,36	0,42
Mapoly0080s0015	0,49	2,05	Mapoly0080s0015	4,82	0,21	Mapoly0080s0015	2,36	0,42
Mapoly0043s0116	1,02	0,98	Mapoly0043s0116	2,28	0,44	Mapoly0043s0116	2,32	0,43

Mapoly0048s0003	0,25	4,05	Mapoly0048s0003	9,22	0,11	Mapoly0048s0003	2,28	0,44
Mapoly0008s0198	0,95	1,06	Mapoly0008s0198	2,36	0,42	Mapoly0008s0198	2,23	0,45
Mapoly0051s0021	3,21	0,31	Mapoly0051s0021	0,68	1,47	Mapoly0051s0021	2,19	0,46
Mapoly0071s0078	2,61	0,38	Mapoly0071s0078	0,83	1,20	Mapoly0071s0078	2,17	0,46
Mapoly0043s0075	3,79	0,26	Mapoly0043s0075	0,55	1,81	Mapoly0043s0075	2,10	0,48
Mapoly0014s0065	0,77	1,31	Mapoly0014s0065	2,60	0,38	Mapoly0014s0065	1,99	0,50
Mapoly0014s0109	2,05	0,49	Mapoly0014s0109	0,92	1,09	Mapoly0014s0109	1,89	0,53
Mapoly0161s0004	0,65	1,54	Mapoly0161s0004	2,87	0,35	Mapoly0161s0004	1,86	0,54
Mapoly0008s0199	0,45	2,20	Mapoly0008s0199	4,10	0,24	Mapoly0008s0199	1,86	0,54
Mapoly0130s0043	0,45	2,24	Mapoly0130s0043	4,15	0,24	Mapoly0130s0043	1,86	0,54
Mapoly0098s0022	1,78	0,56	Mapoly0098s0022	1,04	0,96	Mapoly0098s0022	1,85	0,54
Mapoly0087s0010	0,87	1,15	Mapoly0087s0010	2,09	0,48	Mapoly0087s0010	1,82	0,55
Mapoly0014s0035	1,03	0,97	Mapoly0014s0035	1,75	0,57	Mapoly0014s0035	1,80	0,56
Mapoly0066s0038	1,72	0,58	Mapoly0066s0038	1,03	0,97	Mapoly0066s0038	1,78	0,56
Mapoly0180s0004	1,33	0,75	Mapoly0180s0004	1,31	0,77	Mapoly0180s0004	1,74	0,58
Mapoly0004s0229	1,05	0,96	Mapoly0004s0229	1,54	0,65	Mapoly0004s0229	1,61	0,62
Mapoly0074s0005	5,10	0,20	Mapoly0074s0005	0,31	3,20	Mapoly0074s0005	1,59	0,63
Mapoly0083s0026	0,58	1,74	Mapoly0083s0026	2,69	0,37	Mapoly0083s0026	1,55	0,65
Mapoly0073s0071	0,49	2,03	Mapoly0073s0071	3,02	0,33	Mapoly0073s0071	1,49	0,67
Mapoly0082s0019	0,47	2,12	Mapoly0082s0019	3,07	0,33	Mapoly0082s0019	1,45	0,69
Mapoly0117s0015	0,38	2,66	Mapoly0117s0015	3,78	0,26	Mapoly0117s0015	1,43	0,70
Mapoly0218s0005	2,55	0,39	Mapoly0218s0005	0,56	1,79	Mapoly0218s0005	1,42	0,70
Mapoly0069s0092	0,63	1,58	Mapoly0069s0092	2,23	0,45	Mapoly0069s0092	1,41	0,71
Mapoly0049s0102	1,62	0,62	Mapoly0049s0102	0,83	1,21	Mapoly0049s0102	1,34	0,75
Mapoly0041s0120	2,16	0,46	Mapoly0041s0120	0,61	1,64	Mapoly0041s0120	1,32	0,76
Mapoly0206s0001	4,01	0,25	Mapoly0206s0001	0,30	3,30	Mapoly0206s0001	1,22	0,82
Mapoly0160s0014	0,65	1,55	Mapoly0160s0014	1,86	0,54	Mapoly0160s0014	1,20	0,83
Mapoly0248s0003	0,48	2,10	Mapoly0248s0003	2,41	0,41	Mapoly0248s0003	1,15	0,87
Mapoly0008s0265	4,33	0,23	Mapoly0008s0265	0,26	3,79	Mapoly0008s0265	1,14	0,88
Mapoly0026s0014	0,28	3,60	Mapoly0026s0014	4,10	0,24	Mapoly0026s0014	1,14	0,88
Mapoly0004s0015	2,23	0,45	Mapoly0004s0015	0,49	2,02	Mapoly0004s0015	1,10	0,91
Mapoly0003s0265	0,43	2,31	Mapoly0003s0265	2,49	0,40	Mapoly0003s0265	1,08	0,93
Mapoly0032s0137	2,86	0,35	Mapoly0032s0137	0,38	2,66	Mapoly0032s0137	1,08	0,93
Mapoly0057s0094	2,47	0,40	Mapoly0057s0094	0,41	2,43	Mapoly0057s0094	1,02	0,98
Mapoly0064s0027	0,64	1,56	Mapoly0064s0027	1,57	0,64	Mapoly0064s0027	1,01	0,99
Mapoly0074s0006	2,39	0,42	Mapoly0074s0006	0,41	2,42	Mapoly0074s0006	0,99	1,01
Mapoly0869s0001	1,40	0,71	Mapoly0869s0001	0,62	1,60	Mapoly0869s0001	0,88	1,14
Mapoly0177s0021	0,48	2,08	Mapoly0177s0021	1,75	0,57	Mapoly0177s0021	0,84	1,19
Mapoly0105s0050	1,70	0,59	Mapoly0105s0050	0,48	2,07	Mapoly0105s0050	0,82	1,22
Mapoly0117s0017	0,46	2,15	Mapoly0117s0017	1,76	0,57	Mapoly0117s0017	0,82	1,22
Mapoly0104s0030	2,28	0,44	Mapoly0104s0030	0,36	2,80	Mapoly0104s0030	0,82	1,23
Mapoly0141s0002	1,53	0,65	Mapoly0141s0002	0,53	1,89	Mapoly0141s0002	0,81	1,23
Mapoly0024s0020	0,37	2,71	Mapoly0024s0020	2,08	0,48	Mapoly0024s0020	0,77	1,30
Mapoly0001s0462	1,41	0,71	Mapoly0001s0462	0,54	1,86	Mapoly0001s0462	0,76	1,32
Mapoly0005s0101	0,48	2,10	Mapoly0005s0101	1,54	0,65	Mapoly0005s0101	0,73	1,36
Mapoly0010s0214	1,55	0,64	Mapoly0010s0214	0,44	2,25	Mapoly0010s0214	0,69	1,45

Mapoly0068s0069	1,05	0,96	Mapoly0068s0069	0,63	1,59	Mapoly0068s0069	0,66	1,52
Mapoly0038s0083	0,89	1,12	Mapoly0038s0083	0,73	1,37	Mapoly0038s0083	0,65	1,53
Mapoly0112s0025	2,38	0,42	Mapoly0112s0025	0,27	3,74	Mapoly0112s0025	0,64	1,57
Mapoly0015s0198	0,43	2,31	Mapoly0015s0198	1,44	0,70	Mapoly0015s0198	0,62	1,61
Mapoly0020s0097	2,81	0,36	Mapoly0020s0097	0,22	4,55	Mapoly0020s0097	0,62	1,62
Mapoly0078s0058	0,22	4,53	Mapoly0078s0058	2,79	0,36	Mapoly0078s0058	0,62	1,62
Mapoly0122s0035	1,43	0,70	Mapoly0122s0035	0,42	2,35	Mapoly0122s0035	0,61	1,65
Mapoly0032s0069	1,57	0,64	Mapoly0032s0069	0,38	2,65	Mapoly0032s0069	0,59	1,68
Mapoly0155s0023	1,11	0,90	Mapoly0155s0023	0,53	1,90	Mapoly0155s0023	0,59	1,71
Mapoly0047s0075	1,28	0,78	Mapoly0047s0075	0,46	2,19	Mapoly0047s0075	0,58	1,71
Mapoly0075s0048	1,76	0,57	Mapoly0075s0048	0,33	3,03	Mapoly0075s0048	0,58	1,72
Mapoly0057s0007	3,10	0,32	Mapoly0057s0007	0,18	5,48	Mapoly0057s0007	0,57	1,77
Mapoly0006s0004	0,74	1,36	Mapoly0006s0004	0,77	1,30	Mapoly0006s0004	0,57	1,77
Mapoly0131s0018	0,24	4,11	Mapoly0131s0018	2,32	0,43	Mapoly0131s0018	0,56	1,77
Mapoly0117s0049	0,61	1,64	Mapoly0117s0049	0,90	1,11	Mapoly0117s0049	0,55	1,82
Mapoly0115s0066	1,05	0,96	Mapoly0115s0066	0,52	1,92	Mapoly0115s0066	0,55	1,83
Mapoly0029s0102	0,36	2,78	Mapoly0029s0102	1,51	0,66	Mapoly0029s0102	0,55	1,83
Mapoly0116s0046	1,25	0,80	Mapoly0116s0046	0,43	2,34	Mapoly0116s0046	0,53	1,87
Mapoly0071s0082	0,50	2,01	Mapoly0071s0082	1,07	0,93	Mapoly0071s0082	0,53	1,87
Mapoly0028s0010	1,26	0,79	Mapoly0028s0010	0,42	2,38	Mapoly0028s0010	0,53	1,89
Mapoly0076s0091	0,86	1,17	Mapoly0076s0091	0,61	1,64	Mapoly0076s0091	0,52	1,91
Mapoly0160s0031	0,32	3,10	Mapoly0160s0031	1,60	0,63	Mapoly0160s0031	0,52	1,94
Mapoly0016s0174	0,81	1,24	Mapoly0016s0174	0,62	1,61	Mapoly0016s0174	0,50	2,00
Mapoly0002s0336	0,79	1,27	Mapoly0002s0336	0,63	1,58	Mapoly0002s0336	0,50	2,01
Mapoly0001s0227	0,94	1,06	Mapoly0001s0227	0,52	1,93	Mapoly0001s0227	0,49	2,05
Mapoly0053s0099	0,57	1,75	Mapoly0053s0099	0,85	1,17	Mapoly0053s0099	0,49	2,06
Mapoly0136s0006	0,58	1,71	Mapoly0136s0006	0,82	1,21	Mapoly0136s0006	0,48	2,08
Mapoly0033s0034	1,36	0,74	Mapoly0033s0034	0,35	2,85	Mapoly0033s0034	0,48	2,10
Mapoly0046s0063	0,95	1,05	Mapoly0046s0063	0,49	2,03	Mapoly0046s0063	0,47	2,13
Mapoly0082s0058	0,76	1,31	Mapoly0082s0058	0,61	1,63	Mapoly0082s0058	0,47	2,14
Mapoly0025s0006	0,77	1,29	Mapoly0025s0006	0,59	1,70	Mapoly0025s0006	0,46	2,20
Mapoly0088s0036	0,89	1,12	Mapoly0088s0036	0,50	2,01	Mapoly0088s0036	0,44	2,26
Mapoly0032s0076	0,95	1,06	Mapoly0032s0076	0,47	2,14	Mapoly0032s0076	0,44	2,26
Mapoly0127s0010	1,18	0,84	Mapoly0127s0010	0,37	2,68	Mapoly0127s0010	0,44	2,27
Mapoly0117s0013	0,20	4,90	Mapoly0117s0013	2,11	0,47	Mapoly0117s0013	0,43	2,32
Mapoly0095s0019	0,31	3,25	Mapoly0095s0019	1,37	0,73	Mapoly0095s0019	0,42	2,37
Mapoly0063s0074	0,70	1,42	Mapoly0063s0074	0,59	1,70	Mapoly0063s0074	0,41	2,42
Mapoly0012s0165	0,91	1,10	Mapoly0012s0165	0,44	2,25	Mapoly0012s0165	0,40	2,48
Mapoly0024s0073	0,31	3,25	Mapoly0024s0073	1,30	0,77	Mapoly0024s0073	0,40	2,51
Mapoly0084s0086	2,65	0,38	Mapoly0084s0086	0,15	6,71	Mapoly0084s0086	0,40	2,53
Mapoly0104s0015	0,78	1,29	Mapoly0104s0015	0,51	1,97	Mapoly0104s0015	0,39	2,53
Mapoly0140s0036	0,73	1,37	Mapoly0140s0036	0,54	1,85	Mapoly0140s0036	0,39	2,53
Mapoly0116s0048	0,71	1,41	Mapoly0116s0048	0,55	1,82	Mapoly0116s0048	0,39	2,56
Mapoly0196s0013	0,52	1,93	Mapoly0196s0013	0,75	1,33	Mapoly0196s0013	0,39	2,57
Mapoly0084s0065	0,55	1,82	Mapoly0084s0065	0,70	1,43	Mapoly0084s0065	0,38	2,62
Mapoly0015s0104	1,08	0,92	Mapoly0015s0104	0,35	2,83	Mapoly0015s0104	0,38	2,62

Mapoly0003s0101	0,99	1,01	Mapoly0003s0101	0,38	2,61	Mapoly0003s0101	0,38	2,63
Mapoly0069s0095	0,46	2,15	Mapoly0069s0095	0,80	1,25	Mapoly0069s0095	0,37	2,70
Mapoly0084s0009	0,63	1,58	Mapoly0084s0009	0,58	1,73	Mapoly0084s0009	0,37	2,74
Mapoly0002s0096	1,16	0,86	Mapoly0002s0096	0,31	3,24	Mapoly0002s0096	0,36	2,79
Mapoly0154s0011	1,00	1,00	Mapoly0154s0011	0,36	2,80	Mapoly0154s0011	0,36	2,80
Mapoly0059s0076	0,48	2,10	Mapoly0059s0076	0,73	1,37	Mapoly0059s0076	0,35	2,88
Mapoly0028s0038	0,70	1,42	Mapoly0028s0038	0,49	2,05	Mapoly0028s0038	0,34	2,91
Mapoly0217s0009	0,32	3,13	Mapoly0217s0009	1,07	0,94	Mapoly0217s0009	0,34	2,93
Mapoly0090s0078	0,34	2,91	Mapoly0090s0078	0,99	1,01	Mapoly0090s0078	0,34	2,93
Mapoly0274s0001	0,17	5,78	Mapoly0274s0001	1,94	0,52	Mapoly0274s0001	0,34	2,98
Mapoly0108s0058	1,01	0,99	Mapoly0108s0058	0,33	3,06	Mapoly0108s0058	0,33	3,04
Mapoly0053s0104	0,91	1,10	Mapoly0053s0104	0,35	2,87	Mapoly0053s0104	0,32	3,15
Mapoly0125s0001	0,28	3,52	Mapoly0125s0001	1,11	0,90	Mapoly0125s0001	0,32	3,16
Mapoly0081s0076	0,62	1,62	Mapoly0081s0076	0,51	1,95	Mapoly0081s0076	0,32	3,17
Mapoly0009s0072	0,66	1,53	Mapoly0009s0072	0,48	2,09	Mapoly0009s0072	0,31	3,19
Mapoly0316s0001	1,35	0,74	Mapoly0316s0001	0,23	4,38	Mapoly0316s0001	0,31	3,24
Mapoly0117s0048	0,26	3,78	Mapoly0117s0048	1,12	0,89	Mapoly0117s0048	0,30	3,36
Mapoly0070s0014	0,84	1,19	Mapoly0070s0014	0,34	2,93	Mapoly0070s0014	0,29	3,49
Mapoly0004s0012	0,26	3,87	Mapoly0004s0012	1,09	0,92	Mapoly0004s0012	0,28	3,55
Mapoly0193s0003	0,13	7,44	Mapoly0193s0003	2,09	0,48	Mapoly0193s0003	0,28	3,56
Mapoly0135s0042	0,29	3,43	Mapoly0135s0042	0,96	1,04	Mapoly0135s0042	0,28	3,57
Mapoly0062s0101	0,27	3,73	Mapoly0062s0101	1,00	1,00	Mapoly0062s0101	0,27	3,73
Mapoly0148s0031	0,61	1,63	Mapoly0148s0031	0,44	2,29	Mapoly0148s0031	0,27	3,74
Mapoly0062s0017	0,83	1,21	Mapoly0062s0017	0,31	3,19	Mapoly0062s0017	0,26	3,85
Mapoly0122s0009	1,27	0,79	Mapoly0122s0009	0,20	4,90	Mapoly0122s0009	0,26	3,85
Mapoly0056s0050	0,81	1,23	Mapoly0056s0050	0,32	3,15	Mapoly0056s0050	0,26	3,88
Mapoly0117s0024	0,12	8,59	Mapoly0117s0024	2,12	0,47	Mapoly0117s0024	0,25	4,05
Mapoly0009s0065	0,54	1,85	Mapoly0009s0065	0,44	2,26	Mapoly0009s0065	0,24	4,18
Mapoly0077s0065	0,32	3,10	Mapoly0077s0065	0,70	1,42	Mapoly0077s0065	0,23	4,40
Mapoly0043s0030	0,68	1,46	Mapoly0043s0030	0,32	3,08	Mapoly0043s0030	0,22	4,51
Mapoly0042s0095	0,29	3,42	Mapoly0042s0095	0,75	1,33	Mapoly0042s0095	0,22	4,55
Mapoly0008s0109	0,60	1,67	Mapoly0008s0109	0,36	2,80	Mapoly0008s0109	0,21	4,67
Mapoly0072s0091	0,67	1,48	Mapoly0072s0091	0,31	3,19	Mapoly0072s0091	0,21	4,73
Mapoly0004s0309	0,48	2,09	Mapoly0004s0309	0,44	2,28	Mapoly0004s0309	0,21	4,77
Mapoly0062s0005	0,34	2,96	Mapoly0062s0005	0,59	1,69	Mapoly0062s0005	0,20	4,99
Mapoly0044s0060	0,30	3,38	Mapoly0044s0060	0,65	1,54	Mapoly0044s0060	0,19	5,21
Mapoly0032s0077	1,19	0,84	Mapoly0032s0077	0,16	6,41	Mapoly0032s0077	0,19	5,40
Mapoly0176s0016	2,51	0,40	Mapoly0176s0016	0,07	13,59	Mapoly0176s0016	0,18	5,42
Mapoly0108s0063	0,62	1,62	Mapoly0108s0063	0,30	3,36	Mapoly0108s0063	0,18	5,46
Mapoly0078s0015	1,15	0,87	Mapoly0078s0015	0,16	6,36	Mapoly0078s0015	0,18	5,51
Mapoly0196s0011	0,37	2,72	Mapoly0196s0011	0,49	2,05	Mapoly0196s0011	0,18	5,56
Mapoly0050s0013	1,15	0,87	Mapoly0050s0013	0,15	6,52	Mapoly0050s0013	0,18	5,66
Mapoly0001s0100	0,20	4,92	Mapoly0001s0100	0,86	1,17	Mapoly0001s0100	0,17	5,74
Mapoly0144s0027	0,07	14,85	Mapoly0144s0027	2,57	0,39	Mapoly0144s0027	0,17	5,78
Mapoly0333s0001	0,44	2,26	Mapoly0333s0001	0,38	2,64	Mapoly0333s0001	0,17	5,98
Mapoly0072s0046	1,10	0,91	Mapoly0072s0046	0,15	6,67	Mapoly0072s0046	0,16	6,06

Mapoly0193s0011	1,01	0,99	Mapoly0193s0011	0,16	6,34	Mapoly0193s0011	0,16	6,29
Mapoly0003s0166	0,31	3,20	Mapoly0003s0166	0,48	2,06	Mapoly0003s0166	0,15	6,61
Mapoly0193s0020	0,59	1,70	Mapoly0193s0020	0,24	4,17	Mapoly0193s0020	0,14	7,08
Mapoly0098s0030	0,20	4,90	Mapoly0098s0030	0,69	1,45	Mapoly0098s0030	0,14	7,10
Mapoly0006s0189	0,64	1,57	Mapoly0006s0189	0,21	4,80	Mapoly0006s0189	0,13	7,52
Mapoly0023s0008	0,36	2,78	Mapoly0023s0008	0,37	2,72	Mapoly0023s0008	0,13	7,55
Mapoly0447s0001	0,64	1,55	Mapoly0447s0001	0,16	6,31	Mapoly0447s0001	0,10	9,80
Mapoly0092s0055	0,10	9,60	Mapoly0092s0055	0,93	1,08	Mapoly0092s0055	0,10	10,33
Mapoly0193s0021	0,36	2,78	Mapoly0193s0021	0,26	3,82	Mapoly0193s0021	0,09	10,59
Mapoly0022s0141	1,12	0,89	Mapoly0022s0141	0,07	14,24	Mapoly0022s0141	0,08	12,71
Mapoly0007s0041	0,76	1,32	Mapoly0007s0041	0,10	9,88	Mapoly0007s0041	0,08	13,03
